# Light-XAI: a CADx for explainable cervical cancer detection via attention-based lightweight convolutional neural networks and layer-wise feature fusion

**DOI:** 10.1186/s13040-026-00540-6

**Published:** 2026-04-10

**Authors:** Omneya Attallah

**Affiliations:** 1 Department of Electronics and Communications Engineering, College of Engineering and Technology, Arab Academy for Science, Technology, and Maritime Transport, Alexandria, 21937 Egypt; 2 Wearables, Biosensing, and Biosignal Processing Laboratory, Arab Academy for Science, Technology, and Maritime Transport, Alexandria, 21937 Egypt

**Keywords:** Cervical cancer, Computer-aided diagnosis, Self-attention, Convolutional neural networks, Deep learning, Feature fusion, Explainable artificial intelligence

## Abstract

Cervical cancer, the fourth most common cancer, requires prompt identification for treatment. The Pap smear test is the standard for cervical cancer detection and diagnosis, but it is complicated and laborious, and its efficacy depends on medical professionals. Computer-aided detection and diagnosis (CADx) systems employ deep learning, especially convolutional neural networks (CNN), to improve the precision and efficiency of cervical cancer diagnosis. Nevertheless, conventional CNNs do not inherently utilise instinctive methods of evaluating the relevance of features, as a qualified medical professional would, and instead assess features more broadly. In addition, CNNs are extremely efficient at extracting spatially invariant local features. Self-attention mechanisms allow CNNs to prioritise semantically significant clinical feature regions, gather relevant information with long-distance connections, and could efficiently model global-level features. To this end, this study integrates the self-attention mechanism into a CNN architecture and presents “Light-XAI”, a comprehensive lightweight explainable AI (XAI) CADx system for cervical cancer classification from Pap smear images. The system employs combinations of lightweight CNNs and applies the local gradient-weighted class activation mapping (Grad-CAM) technique for the explainability of decisions made. Furthermore, deep features from each CNN trained with Pap smear images are derived from two deep layers opposing current CADxs which either obtain features from a single layer or perform end-to-end CNN classification. In addition, features extracted from both deep layers of self-attention CNN are combined and decreased using a feature reduction method. Furthermore, the attributes of the three self-attention CNN models are fused, and subsequently a feature selection method is employed to choose the most impactful features. The results demonstrate that Light-XAI has achieved an accuracy of 97.27% and 99.82% for the SIPaKMeD and Mendeley liquid-based cytology (LBC) datasets, respectively, suppressing most of the present CADx. This superior performance allows Light-XAI to serve as a powerful tool to categorise cervical cancer. In addition, it shows the potential for improving diagnostic precision, allowing for the adoption of advanced methods in the field of medical image analysis.

**Supplementary information:**

The online version contains supplementary material available at 10.1186/s13040-026-00540-6.

## Introduction

Cervical cancer is a [1] malignancy that specifically targets the cervix in women [1]. It ranks as the fourth largest contributor to cancer-related mortality in women globally [2]. However, cervical cancer has emerged as the next most prevalent form of cancer in women, particularly in developing nations, after breast cancer. It claims more than 700 lives each day and is projected to cause approximately 400,000 deaths yearly by 2030, with almost ninety percent of those fatalities occurring in nations with poor infrastructure [3]. It is estimated that around 13,960 women in the United States will be diagnosed with aggressive cervical cancer in 2023, according to estimates. Approximately 4310 fatalities are expected to occur in the United States as a result of this condition within that specific period [4].

Cervical cancer is an extremely curable form of cancer when detected in its early stages. Vaccination, particularly in young girls, can successfully prevent cervical cancer by protecting against human papillomavirus (HPV). This sexually transmitted disease is responsible for more than 95% of cases of cervical cancer. The most effective way for older sexually promiscuous women to be examined for cervical cancer [3]. Several examination methods have been used and are well documented for cervical cancer diagnosis. Methods used for this purpose include cytology, also known as the Pap smear procedure [5], cervigram scanning [6], and colposcopy screening [7]. However, particular approaches, such as cervigram scanning and colposcopy screening, require a significant financial commitment due to the need for advanced facilities and supplies.

Pap smear examination is the most common screening method conducted in developing nations due to its widespread recognition, easy accessibility, and cost-effectiveness. During this examination, samples are obtained by employing a brush or spatula to collect smears. The medical professional stains the smear and examines it under a microscope to identify abnormal cells. The Pap test is crucial in reducing mortality rates associated with cervical cancer. However, this procedure has typically been performed manually by skilled professionals and the outcome is entirely dependent on the pathologist [8]. Pathologists can observe only a maximum of 50 to sixty slides in one working day. The laborious nature of this procedure imposes a substantial workload on professionals and clinicians due to the immense number of scans that require analysis. Consequently, finding a proficient cytotechnologist to analyse Pap smear slides and perform screenings continues to be difficult, which leads to the necessity to create a computerised system capable of automatically and quickly examining Pap slides [9]. Consequently, this will decrease the workload of pathologists and allow them to enhance the quantity and quality of their diagnostic evaluations [10].

In this context, an evolution in medical diagnosis is being heralded by the emergence of artificial intelligence (AI) techniques, particularly deep learning and machine learning in the field of medical image processing. The combination of these technological breakthroughs makes it possible to overcome the restrictions of conventional screening methods, leading to a new era of precision and effectiveness in medical diagnosis. In recent years, computer-aided detection and diagnosis (CADx) systems incorporating deep learning techniques have demonstrated remarkably high rates of success in diagnosing various diseases, including breast cancer [11–13], eye infections [14], brain abnormalities [15, 16], colorectal diseases [17, 18], leukemia [19], and skin cancer [20–22]. CADx can greatly aid professional clinicians and pathologists by allowing an independent and swift diagnosis of cervical cancer. CADx imaging systems not only help detect and monitor cervical cancer, but also serve as a tool that allows the pathologist to quickly analyse samples and generate a comprehensive report that assists in their decision-making process [23]. According to several review articles [24–26], convolutional neural networks (CNNs) are one of the most commonly used deep learning structures for image analysis. The analysis of medical photos using CNNs has been widely applied to the segmentation, detection, and diagnosis of different types of illness [18, 27, 28]. The efficacy of CNNs can be primarily attributed to the convolution operation and the depth of the layers they possess. The deeper the CNN, the greater the percentage of hyperparameters that comprise it, but the greater the chance of overfitting and greater complexity [29].

Recent research has widely used CNNs in CADx systems for cervical cancer. Numerous limitations have been found in the literature. For example, several current CADx systems depend on an individual CNN approach, which is used for end-to-end classification or feature extraction; however, employing CNN ensembles usually improves performance [30]. In addition, the features obtained from these CNNs are frequently of high dimensionality, which raises the complexity of classification. Furthermore, current CADx systems frequently count on features obtained from simply one deep layer, even though various levels of information are revealed through distinct layers. However, improved classification performance can be achieved by integrating features from several layers [31, 32]. Furthermore, the lack of transparency in the training procedure and feature extraction hinders the use of existing CADx systems by clinicians and patients. Insufficient explainability in deep learning models poses challenges for clinicians in terms of trust and interpretation of outcomes. Another limitation found in the literature is that a significant number of current CNN architectures for cervical cancer require a thorough hyperparameter tuning, which can result in overfitting due to the substantial parameter count [33]. As a solution to this issue, it is possible to mitigate overfitting by decreasing the number of features and employing compact deep learning topologies [34]. Additionally, conventional CNNs, although successful in capturing local features, may have difficulties in dynamically analysing and comprehending the associated connections among various parts of an image [35]. In contrast, the self-attention mechanism has the ability to encapsulate information from the complete input sequence, representing global-level characteristics [36]. In order to prevent overfitting, self-attention can improve visibility by highlighting significant characteristics and eliminating unnecessary ones [36, 37].

Motivated by the limitations of existing cervical cancer classification systems, this study proposes Light-XAI, a novel explainable artificial intelligence (XAI) CADx system. It is derived from the successes of self-attention mechanisms in the fields of machine translation and computer vision [38, 39]. Light-XAI integrates self-attention modules into a lightweight CNN structure. Light-XAI incorporates three pretrained compact CNN models, each enhanced with a self-attention module. Both the self-attention layer and the final pooling layer are deep layers in each CNN from which features are retrieved instead of relying on features from a single layer. Subsequently, the aforementioned characteristics are combined, condensed, and merged using the discrete wavelet transform (DWT) to generate an exhaustive representation of the pap smear photo. At last, a feature selection procedure determines the most significant features for classification to avoid overfitting and lower classification complexity. In order to improve the clarity of explanation, Light-XAI utilises the local Gradient-weighted Class Activation Mapping (Grad-CAM) XAI technique to visually represent the specific areas of the image that have the greatest impact on the model’s decisions.

The main contributions of this study reside in these particular aspects.

- First, this study will thoroughly explore the integration of self-attention mechanisms to compact convolutional neural network (CNN) architectures in diagnosing cervical cancer from pap smear images. The use of self-attention enables the model to extract local as well as global features to further improve the models’ ability to identify complex relationships and patterns that exist within the data.
- Secondly, unlike many current methods that depend on sophisticated CNNs with a large number of deep layers and parameters, Light-XAI employs lightweight CNNs named EfficientNetB0, MobileNet, and ResNet-18. This increases the speed of detection and decreases computational complexity, making Light-XAI more useful for practical applications.
- Third, most of the existing CAD did not interpret how decisions were made; By integrating Grad-CAM, the proposed model achieves a distinctive degree of explainability. Through visualisation of the specific areas of the image that have the greatest impact on classification, Light-XAI provides clinicians with a valuable understanding of the decision-making process of the model. This, in turn, promotes trust and probably enhances patient outcomes.
- Fourth, Light-XAI incorporates a DWT-based feature fusion technique that merges features obtained from multiple layers of each CNN, rather than just one layer, as is common in current CADx systems.
- Fifth, it merges deep features of the three self-attention CNNs. This approach integrates the advanced capabilities of the three lightweight structures, thus reducing the reliance on a single CNN.
- Finally, it decreases the dimensionality of the features by choosing the features that are most informative. These improvements boost the model’s capacity to differentiate between various forms of cancer.

The subsequent sections of the article are organised as follows. The second section presents pertinent investigations of the CADx tools developed for the diagnosis of cervical cancer. The third section will provide an overview of the techniques used to develop Light-XAI and will explain the specific steps involved in the process. Following that, the fourth section outlines the experimental design and process, which discloses the particular values of the hyperparameters employed for the self-attention CNNs, as well as the performance indicators deployed to assess Light-XAI. Subsequently, the fifth section presents the findings of the study. Subsequently, the sixth section explains the study’s findings and its limitations. Furthermore, Sect. ;Discussions contains a comprehensive evaluation that compares different existing CADx. The seventh section contains the conclusion.

## Literature survey

The literature contains multiple studies showing the significant advances made by traditional machine learning and deep learning techniques to successfully identifying different types of cancer [40–43]. Deep learning has demonstrated significant potential in the analysis and diagnosis of cervical cancer using Pap smear photos, regularly attaining a high degree of correctness in its results [44–46]. Deep learning techniques, particularly CNN-based CADx models, have been widely used in research to detect cervical cancer from pap smear scans. The use of such modern algorithms has substantially increased the accuracy and effectiveness of the diagnosis of cervical cancer, resulting in better medical findings in this field. This section will discuss the latest CADxs that employ traditional machine learning and deep learning models for the classification of cervical cancer. First, it will discuss those CADx that employed traditional machine learning models. After that, it will present CADx based on individual CNN models. Afterward, it will demonstrate CADx based on CNNs for feature extraction combined with traditional classifiers. Next, the section will illustrate the existing CADx that uses CNN ensembles. Following that, it will present the current CADx that uses vision transformer (ViT) approaches. Later, it presents CADx based on multiple instance learning (MIL). Finally, it will discuss research gaps and motivation.

### CADx based on traditional machine learning models

Earlier CAD workflows used a traditional machine learning methodology that relied on the extraction of manually constructed characteristics from pap smear photos. The study [47] employed C-means clustering to separate cervical cells and subsequently extracted shape and textural information, including the binary histogram Fourier technique (BHF). The quantum-based grasshopper computing algorithm (QGH) is employed to choose characteristics, which are then used as inputs for classifiers. In a separate work [48], the researchers used clustering of C-means to segment cervical cells and subsequently collected textural information, including GLCM, along with geometrical descriptors from these cells. Following that, they employed principal component analysis (PCA) to reduce the dimensionality of the characteristics. Subsequently, KNN is used to categorize cervical cells, achieving an accuracy of 94.86%. The research [49] utilised discrete cosine and wavelet transforms for feature extraction. The fractional coefficients method is employed to diminish the dimensionality of the integrated features. Ultimately, these diminished features are input into seven machine learning classifiers to distinguish between various subgroups of cervical cancer, resulting in an accuracy of 81.11%. The paper [50] introduced a CAD with two steps. The objective of the very first step is to acquire textural variables from both the cytoplasm and nucleolus concurrently. The pap smear slides were divided with a thresholding technique. A texture attribute known as modified uniform local ternary patterns (MULTP) is presented to characterise the local textural characteristics. Subsequently, these characteristics were input into a classical neural network, and its settings were refined by a genetic process, achieving an accuracy of 98.9%.

### CADx based on individual CNN models

Several studies employed individual deep-learning models. The study [23] used a 4-phase optimisation procedure to develop a personalised deep learning XAI classifier capable of accurately differentiating between 4 severity classes of cervical cancer using liquid-cytology images. The ultimate classifier achieves an accuracy exceeding 97% for a total of 4 classes. The authors of the study [51] proposed a CADx system that uses pre-trained CNNs to automatically analyse Pap smear images and classify them into seven different classes. Researchers conducted a performance comparison of 13 distinct pretrained CNN models and determined that DenseNet-201 was the most efficient. It showed encouraging results in accurately classifying cervical cancer from Pap smear photos. CytoBrain is an CADx system that was first presented in a publication by Chen et al. in 2021 [52]. The system consists of three primary modules: cell segmentation, classification, and visualised human-aided diagnostics. To obtain an individual cell picture, the pictures were first divided into segments using the Speeded Up Robust Features (SURF) and Otsu thresholding methods. Subsequently, these pictures were sent to a VGG CNN. The human-aided diagnostic component performed the assessment by combining all the results of the classification of cell images and presenting the outcomes visually reaching an accuracy of 97.80% and 94,81% for the SIPaKMeD and Herlev databases.

The paper [53] presented a new strategy for categorising cervical cancer by employing a blend of approaches. The system employed a bilinear CNN for extracting features and used random projections to lower their dimension. This has the potential to allow for more effective processing and evaluation, while also ensuring precision in the classification of malignant and normal cervical tissue types. Significantly, an accuracy of 99.83% was attained for binary categorization, while an accuracy of 95.30% was attained in the setting of the classification of multiple classes, which included seven different categories. The study [54] introduced a method for classifying cervical cells using a customised CNN. After segmenting the Pap smear images, the enhanced photos of cervical cells were subjected to a deep CNN with 4 layers of convolution. A CNN with a straightforward design is proposed, which achieves an accuracy of 0.9113%. While article [55] employed deep learning in two distinct manners. The first method utilised pre-trained CNN models as feature extractors and various machine learning techniques for classification. Additionally, transfer learning was adopted using pre-trained CNNs for identifying images of cervical cancer. By employing the initial approach of using a pre-trained CNN for feature extraction, ResNet-50 attains the utmost classification success rate of 92.03%. By employing the second method of refining preexisting models, GoogleNet has achieved an exceptional rate of classification of 96.01%.

### CADx based on CNNs for feature extraction combined with traditional classifiers

In the study conducted in reference [56], the researchers resized photos of the Pap smear datasets to various dimensions. Then those cropped photos were utilised to separately learn DarkNet-19 CNN and DarkNet-53 CNN individually. These attributes obtained from such models are significant in size. Consequently, the features were subsequently individually fed into the neighbourhood component analysis (NCA) to reduce their dimensions. Ultimately, the decreased attributes were used to construct a support vector machine (SVM), resulting in accuracy levels of 98.26% and 99.47% for the SIPaKMeD and Mendeley LBC databases, respectively. The article [57] presented a tool that utilised deep features to classify cervical cancer cases based on Pap smear cytology images. The tool consisted of ResNet18 and GoogleNet CNNs and a feature selection approach known as an Opposition-based Harmony Search Algorithm (O-bHSA). The classification of these features was performed using conventional classifiers such as SVM, multilayer perceptron (MLP), and k-nearest neighbours (KNN).

The study [58] involved extracting deep attributes from ShuffleNet and a customised model known as Cervical Net. These models were developed using the SIPaKMeD dataset. Subsequently, the authors employed canonical component analysis (CCA) to merge attributes and achieve a reduction in features, resulting in a total of 544 features. The extracted attributes were subsequently inputted into multiple classifiers, resulting in a high accuracy rate of 99.1%. The reference study [59] introduced a CADx system that used a two-step feature selection method to decrease the number of variables acquired from different CNN models. Afterward, the diminished attributes are exploited to feed the SVM to produce the final predictions using the Mendeley LBC, Herlev, and SIPaKMeD databases. However, the study [31] merged deep features from three lightweight CNNs, including DarkNet19, ResNet18, and MobileNet, and then employed a feature selection approach to reduce features. In contrast, the paper [60] integrated manually designed attributes with the deep attributes of various CNNs using the Mendeley LBC dataset.

### CADx based on CNN ensembles

Additional research has suggested CADx models that rely on various convolutional CNNs. In the study [61], the authors integrated deep learning with the Genetic Algorithm (GA), a naturalistic metaheuristic technique, to classify cervical cells. GoogLeNet and ResNet-18 CNNs were used to compensate for the limited amount of data and retrieve complex features from the photos. The features that were obtained are enhanced by using a GA for selecting the most relevant features. This is combined with the SVM classifier to perform the last classification step. The accuracy reached 98.94% in the SIPaKMeD dataset and 99.07% in the LBC dataset. Whereas the authors of reference [62] extracted features from MobileNet and DenseNet121. These characteristics were concatenated and then the authors used long- and short-term memory (LSTM) to perform classification, reaching 95.80%.

Furthermore, the article [63] introduced a collection of advanced models, called Ensemble Networks Based on Mean and Standard Deviation (MSENet), designed to identify cervical cancer. The combined model comprises three CNNs: Xception, Inception V3, and VGG-16. The prediction capabilities of these models were enhanced by adopting a new probability improvement technique that considers the mean and standard deviation of confidence values. This approach allowed the general structure to incorporate additional information provided by the foundational classifiers and is specifically designed to suit the attributes of deep learners. Ultimately, the product rule was employed to combine the acquired results and generate conclusive predictions reaching 97.21% and 99.76% accuracy using the SIPaKMeD dataset and the LBC dataset, respectively. The study [64] collected confidence ratings using Inception, MobileNet, and InceptionResNet CNNs. Such results were combined using a fuzzy distance-based aggregation strategy that involved three distance metrics. Compared to the study conducted by [65] created a CADx employing an ensemble approach that combined Inception v3, Xception, and DenseNet-169. The suggested composite approach developed a fuzzy rank-based fusion technique to combine the results of multiple classifiers, specifically CNNs. This technique of fusion incorporated two non-linear functions to enhance the accuracy of the composition. The suggested framework was tested using the SIPaKMeD and Mendeley LBC datasets. Table 1 shows a summary of the recent literature on CAD systems for cervical cancer diagnosis.

**Table 1:** A summary of related works for cervical cancer diagnosis using deep learning-based CADx

Reference	Methodology	Feature Selection	Dataset	Accuracy	Limitations
[69]	Morphological Segmentation + DCWT+ResNet-18	No	Herlev 623 images. 4 classes	97.98%	Utilise the segmentation technique, which is associated with increased complexity. Utilise a single CNN model. Didn’t employ XAI to interpret results. Did not perform feature selection
[58]	Median Filter + Histogram Equalization + Cervical Net + ShuffleNet + CCA	Yes	SIPaKMeD 4049 5 classes	99.1%	Utilise the segmentation technique, which is associated with increased complexity. Utilised a single dataset for model validation. Derived deep attributes from a CNN’s only one layer Didn’t employ XAI to interpret results
[56]	Splitting of each image DarkNet-19 + NCC DarkNet-53 + NCC	Yes	SIPaKMeD 4049 5 classes Mendeley LBC 963 images. 4 classes	98.26% 99.47%	Each image is extensively divided into multiple levels. Large dimensionality of features. Derived deep attributes from a CNN’s only one layer Didn’t employ XAI to interpret results.
[64]	MobileNet+Incetion + InceptionResNet	No	SIPaKMeD 4049 images. 5 classes Mendeley LBC 963 images. 4 classes Herlev 918 images. 2 classes	96.96% 99.68% 98.56%	Derived deep attributes from a CNN’s only one layer. Implement two CNNs that demand substantial computational capacity and resources. Didn’t employ XAI to interpret results. Did not perform feature selection
[62]	ViT+MobileNet	No	SIPaKMeD	97.60%	Didn’t employ XAI to interpret results. Utilised a single dataset for model validation. Did not perform feature selection
[70]	Bilateral filtering + Kapur entropy-based image segmentation + EfficientNet + Stacked Sparse Denoising Autoencoder	No	Herlev 918 images. 7 classes	99.66%	Derived deep attributes from a CNN’s only one layer. Used a single CNN for obtaining attributes. Utilised a single dataset for model validation. Did not perform feature selection
[71]	Median Filter + Histogram Equalization VGG16 + Gradient-weighted Class Activation Mapping (Grad-CAM)	No	Herlev 918 images. 2 classes	91.94%	Used a single CNN for obtaining attributes. Utilised a single dataset for model validation. Performed only binary classification. Low performance. Did not perform feature selection
[68]	Xception + Visual Transformer	Yes	Combined (CRIC 4789 images. 6 classes + SIPaKMeD 4049 images. 5 classes)	91.72%	Used a single CNN for obtaining attributes. Use models that demand a lot of resources and processing power. Large dimensionality of features. Didn’t employ XAI to interpret results.
[72]	Zero Padding AlexNet	No	SIPaKMeD 4049 images. 5 classes)	87.32%	Use models that demand a lot of resources and processing power. Depended on only on4 CNN Utilised a single dataset for model validation. Low performance Didn’t employ XAI to interpret results. Did not perform feature selection
[73]	Median Filter + Histogram Equalization Xception+ Grad-CAM+ Grad-CAM++ core-CAM, Eigen-CAM, and LayerCAM	No	Herlev 918 images. 2 classes	89.0%	Used a single CNN for obtaining attributes. Utilised a single dataset for model validation. Performed only binary classification. Low performance.
[65]	Inception+Xception+DenseNet-169 + Fuzzy rank	No	SIPaKMeD 4049 images. 5 classes) Mendeley LBC 963 images. 4 classes	95.43% 99.23%	Use models that demand a lot of resources and processing power Implement a padding phase. Didn’t employ XAI to interpret results. Did not perform feature selection
[59]	VGG-16 + Inception + DenseNet-121 + ResNet-50 + PCA + GWO	Yes	SIPaKMeD 4049 images. 5 classes Mendeley LBC 963 images. 4 classes Herlev 918 images. 7 classes	97.87% 99.47% 98.32%	Use models that demand a lot of resources and processing power The suggested CAD is complicated. Derived deep attributes from a CNN’s only one layer. Didn’t employ XAI to interpret results.
[74]	ResNet152 + logistic regression	No	SIPaKMeD 4049 images. 2 classes)	98.08%	Used a single CNN for obtaining attributes. Utilised a single dataset for model validation. Performed only binary classification. Low performance. Didn’t employ XAI to interpret results Did not perform feature selection
[52]	Segmentation via SURF and Otsu threshold Compact VGG	No	SIPaKMeD 4049 images. 5 classes) Herlev 918 images. 7 classes	98.43% 94.81%	Utilise the segmentation technique, which is associated with increased complexity. Used a single CNN for obtaining attributes. Didn’t employ XAI to interpret results. Did not perform feature selection
[75]	MobileNet + Gazelle Optimizer Algorithm + stacked extreme learning machine	No	Herlev 918 images. 7 classes	99.38%	Used a single CNN for obtaining attributes. Utilised a single dataset for model validation. Low performance. Didn’t employ XAI to interpret results Did not perform feature selection
[76]	Normalization ResNet50	No	Herlev 918 images. 2 classes	97.0%	Used a single CNN for obtaining attributes. Utilised a single dataset for model validation. Performed only binary classification. Low performance. Didn’t employ XAI to interpret results Did not perform feature selection

### CADx based on ViT models

Other studies investigated employing a ViT deep learning model to perform cervical cancer classification. The authors of the study [66] implemented a CADx that consists of a model that has an input layer that accepts cervical photographs, next to a 3D convolution block that retrieves attributes from the photos. The resultant feature maps undergo down-sampling using a max-pooling block to remove unnecessary details yet retain crucial features. Four ViT models are used to extract effective feature maps with varying levels of complexity. The result of each ViT design is a compact collection of feature maps that represent both spatial and temporal information at a particular level of complexity. The feature maps produced by the ViT models are subsequently fed to the 3D feature pyramid network (FPN) unit for the purpose of concatenating features. The 3D squeeze-and-excitation (SE) block is used to generate effective feature maps by adjusting the attribute outputs of the model according to the connections among various feature maps. This improves the model’s ability to distinguish between different classes. Finally, the feature maps are reduced in size using a 3D average pooling layer. Subsequently, the output is input into a kernel classifier to classify the categories of cervical cancer. The superior performance of the suggested approach is established through simulation findings, demonstrating an accuracy of 98.6%. In the study [67], the authors used a complex structural CADx called the Multi-Axis Vision Transformer (MaxViT). By applying the MaxViT model to the Pap smear data, a streamlined framework that provides exceptional precision and faster data analysis was achieved. To enhance the performance of the suggested model, the authors replaced the MBConv blocks in the MaxViT architecture with ConvNeXtv2 blocks and the MLP blocks with GRN-based MLPs. This change improved the model’s capacity for generalisation while also lowering the number of parameters. The suggested technique exhibited remarkable precision, outperforming previous research, reaching an accuracy rate of 99.02% in the SIPaKMeD dataset and 99.48% on the LBC dataset. In a recent study [68], the authors integrated the attributes of an Xception module and a ViT component using a neural network. This approach achieved an impressive accuracy of 91.72% using an integrated collection of CRIC and SIPaKMeD datasets.

### CADx based on multiple instance learning (MIL) for microscopy

With the increased use of microscopy-based medical image analysis in recent years and its subsequent need for new techniques, such as those found within the multiple instance learning (MIL) framework, many researchers have turned to MIL methods as an alternative solution to dealing with large numbers of whole-slide images (WSI), in particular weakly annotated WSI where only slide-level labels are available. Image patches within each WSI can collectively be called bags. Therefore, the focus of the learning process becomes the identification of discriminative instances in each bag that support or lead to the ultimate classification of the entire bag. Zhou et al. [77] developed an iterative MIL framework in which instance selection is progressively refined and demonstrated increased robustness to weakly annotated WSI classification. Subsequently, Zhou and Lu [78] provided a method to explicitly model critical instances to improve the reliability of the WSI classification. Additionally, hierarchical MIL architectures have been developed that aggregate multiple levels of representations from both local cellular patterns and global tissue context found within large histological images to create more comprehensive representations [79]. Multilevel task-specific MIL methods have been developed that match feature extraction with the morphological characteristics that are specific to the associated task [80]. Each of these MIL-based approaches has shown excellent results in terms of cancer detection and grading at the WSI level with weak supervision. Although this study focuses on cell/image level Pap smear classification using collected public cytology databases, MIL-based WSI systems offer a pertinent additional approach for cervical screening and provide a promising avenue for future research on large-scale slides with limited annotations.

### Research gaps and motivation

After examining the literature, it is evident that numerous CNN-based CADx platforms have been widely employed to classify cervical cancer cell categories. Several CADx tools relied on deep learning structures that demanded significant computational capabilities, thereby complicating the classification procedures. Besides, having huge parameters and deep layers can negatively impact the network’s ability to generalise and result in overfitting. Furthermore, some of these models relied on extracting features from a vast number of dimensions for classification without using a feature reduction or selection technique, thereby increasing the complexity of the learning procedure and prolonging the learning duration. In addition, multiple techniques used a singular CNN to carry out the task of classifying cervical cancer by obtaining deep features. However, the use of numerous deep learning models to extract attributes and classify images would undoubtedly enhance the outcomes. Furthermore, certain CADx platforms require segmentation or image enhancement procedures for diagnostic purposes, thus increasing the intricacy of the CADx. Moreover, most of the existing CADx tools did not employ XAI techniques to explain how predictions were made by a deep learning model. The lack of explanation in the deep learning framework hinders clinicians from making accurate decisions. Additionally, the present CADx systems rely on conventional CNNs that do not natively employ natural techniques to evaluate the significance of attributes, as an experienced physician would. Rather, these CNNs evaluate variables in a more general manner. On the other hand, the majority of current CADx systems do not employ self-attention mechanisms, which enable CNNs to focus on important global areas or gather pertinent information through long-range connections. Using self-attention mechanisms, the resiliency of medical imaging evaluation systems can be improved by giving priority to the locations of essential diagnostic features and can effectively model global features.

To overcome these challenges, this study introduces a novel XAI CADx system named “Light-XAI” to classify cervical cancer. The study integrates the self-attention mechanism into three lightweight CNN architectures. The system uses ensembles of lightweight self-attention CNNs. The suggested CADx system takes advantage of the Grad-CAM approach for interpreting the decision-making process. Furthermore, deep feature extraction is performed from the self-attention layer and the pooling layer of each modified CNN model. Moreover, the dual-layer deep features are fused and diminished using a feature reduction technique. Furthermore, the attributes of the three self-attention CNN models are combined, and a feature selection technique is used to choose the most valuable features. In addition, the Grad-CAM XAI technique is used to elucidate the decision-making process of each CNN and to highlight specific areas in the pap smear image that significantly influence this decision.

Each of the distinct building blocks used in this work (lightweight CNNs, attention mechanisms, feature fusion, traditional classifiers, GradCAM) have been studied in previous research; however, the novelty of this work does not lie in the isolated algorithmic components, rather it lies in the architectural design, the integrated methodology, and the application-driven development of an explainable cervical cytology CADx system.

More specifically, the current work provides an integrated, highly constrained design that addresses the practical limitations identified in current cervical cancer CADx systems. For example, while attention mechanisms are common in CADx systems and used frequently, most systems are built on top of large, complex, transformer-type or end-to-end architectures. The current study introduces lightweight self-attention modules that are placed in semantically significant locations in compact CNNs. This design allows the modelling of nonlocal spatial dependencies relevant to cytological morphology while maintaining computational efficiency, an area that until now has received minimal focus in cervical cytology research.

Currently, nearly all existing CADx systems utilize a single-layer representation of the features (or a single backbone feature set), but the framework proposed in this project will extract features from multiple pooling and self-attention layers in three distinct lightweight CNNs. The framework captures complementary local texture, global context, and attention-related semantics through an explicit combination of the feature extraction layers of multiple CNNs. Note that this work’s innovation is not in the idea of fusing features, but rather in how we systematically fuse deep heterogeneous features between layers and architectures based on the inherent characteristics of cytology data.

The third methodological contribution of this research is the integration of discrete wavelet transforms (DWT) in deep feature space. Instead of applying wavelets at the image level, this approach embodies the mathematical basis for using DWT to fuse and compress multidimensional deep features obtained from different deep layers and corresponding to distinct levels of abstraction. DWT allows for the separation of coarse discriminative features and redundant activations, presenting a more systematic option than simply concatenating or averaging them together, as has been done traditionally in CADx pipelines.

The fourth contribution is employing a statistical method (ANOVA-based feature selection) to rank and select key discriminative features based on interclass variance. This represents a more systematic and statistically sound methodology than the more recent practices of removing features at the end-to-end level or applying heuristically derived dimension reductions. This method improves interpretation and robustness in low-data conditions seen frequently in medical imaging.

The fifth and final contribution to the Light-XAI framework emphasises explainability along with efficacy and medical diagnostic plausibility, rather than simply focusing on measurement accuracy. The combination of using Grad-CAM analysis, utilizing lightweight architecture choices, and measuring computational complexity allows transparent and deployable CADx systems. This is a unique contribution compared to recently conducted studies, many of which allow a trade-off between interpretability or practicality and minimal increases in accuracy.

## Methodology

### Datasets

#### SIPaKMeD dataset

**Fig. 1:**
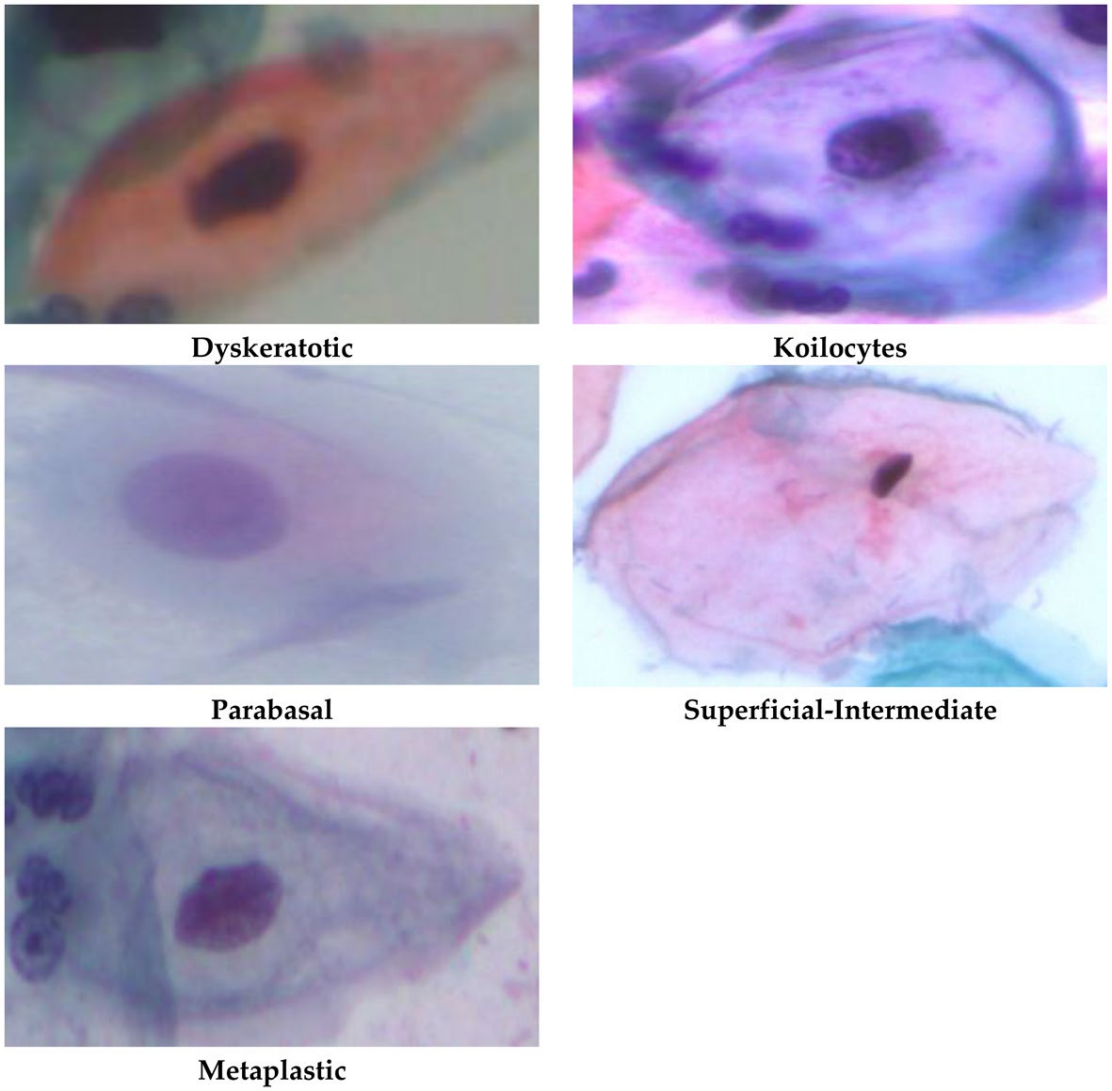
Instances of pictures in the SIPaKMeD dataset

The SIPaKMeD dataset [81] is a useful tool to study the detection of cervical cancer. The dataset comprises 4049 single-cell photos that have been labelled and classified into five categories according to cell morphology by experienced cytopathologists. The different categories consist of two kinds of noncancerous cells (superficial/intermediate and parabasal), two groups of abnormal but nonmalignant cells (koilocytes and dyskeratotic), and one category of non-cancerous metaplastic cells. The allocation of photos in every category is as follows: 813 for dyskeratotic, 825 for Koilocytes, 793 for metaplastic, 787 for Parabasal, and 813 for superficial intermediate. Figure 1 shows instances of photos belonging to the categories that are present in the datas

#### Mendeley liquid-based cytology (LBC)

**Fig. 2:**
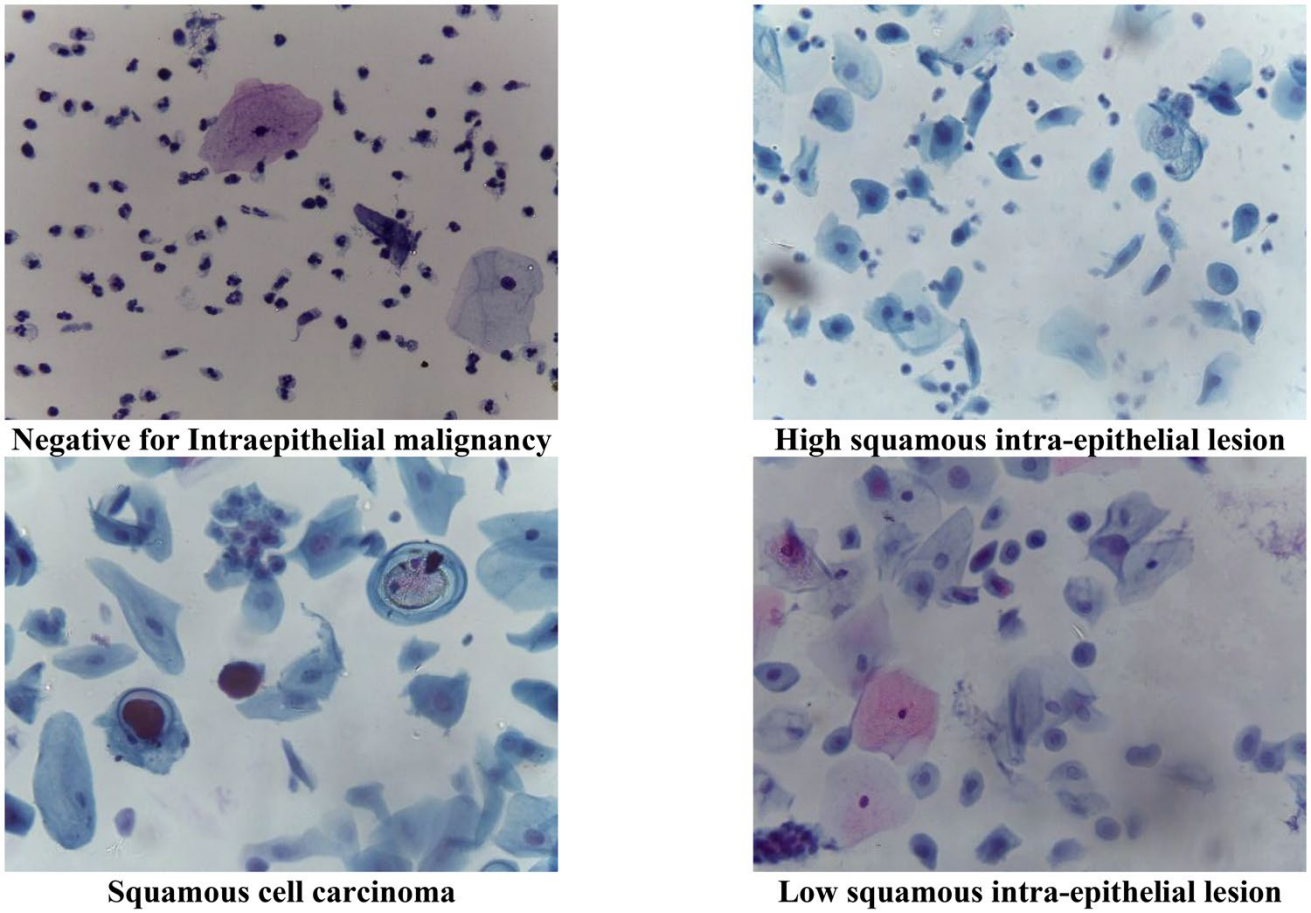
Instances of pictures in the Mendeley LBC dataset

The Mendeley Liquid-Based Cytology (LBC) dataset [82] provides a publicly accessible tool for training and evaluating methods in the field of cervical cancer diagnosis. The database comprises 963 pictures that are categorised into 4 different categories related to pre-cancerous and cancerous tumours: negative for intraepithelial malignancy, low-grade squamous intraepithelial lesion, high-grade squamous intraepithelial lesion, and squamous cell carcinoma. The picture count for every group is as follows: 613 for no intraepithelial malignancy, 113 for high squamous intraepithelial lesion, 163 for low squamous intraepithelial lesion, and 74 for squamous cell carcinoma. Figure 2 displays several instances of pictures in the dataset.

The SIPaKMeD dataset comprises separated single-cell Pap smear photos, while the Mendeley LBC dataset features multicell cytology photos with intricate backgrounds; thus, the natural disparities in resolution, colour tone, and visual appeal are indicative of dataset-specific acquisition and annotation traits rather than preprocessing discrepancies.

### Proposed Light-XAI

The introduced Light-XAI consists of five stages, which are Pap smear image preparation, lightweight self-attention CNNs establishment, dual deep layers feature extraction and integration, self-attention CNN feature fusion and selection, and finally classification. Initially, during the Pap smear image preprocessing stage, in order to maintain uniformity and suitability for the deep learning models, the Pap smear images acquired from both the SIPaKMeD and Mendeley LBC datasets are adjusted to a standard resolution. Furthermore, to enhance the diversity of the training data and enhance the generalisation of the model, data augmentation methods, including random rotation, flipping, and cropping, are implemented. Afterward, during the lightweight self-attention CNN establishment stage, three pretrained CNNs, including EfficientNetB0, MobileNet, and ResNet-18, are chosen as the foundation for Light-XAI. The selection of these models is based on their high efficiency and performance in tasks related to image classification. Each CNN is provided with a self-attention module to improve the extraction of features and preserve long-range dependencies in the pap smear photos. Self-attention systems help the model acquire complex patterns by allowing it to focus on specific areas of the picture that are most important for classification. Deep features are then acquired from two separate layers of each self-attention CNN: the final pooling layer and the self-attention layer in the dual deep layer feature extraction and integration stage. While the layer of self-attention focuses on specific areas, the last pooling layer gathers the global picture information. After that, data from several layers of the network are combined using the DWT method, covering both time-frequency representations.

**Fig. 3:**
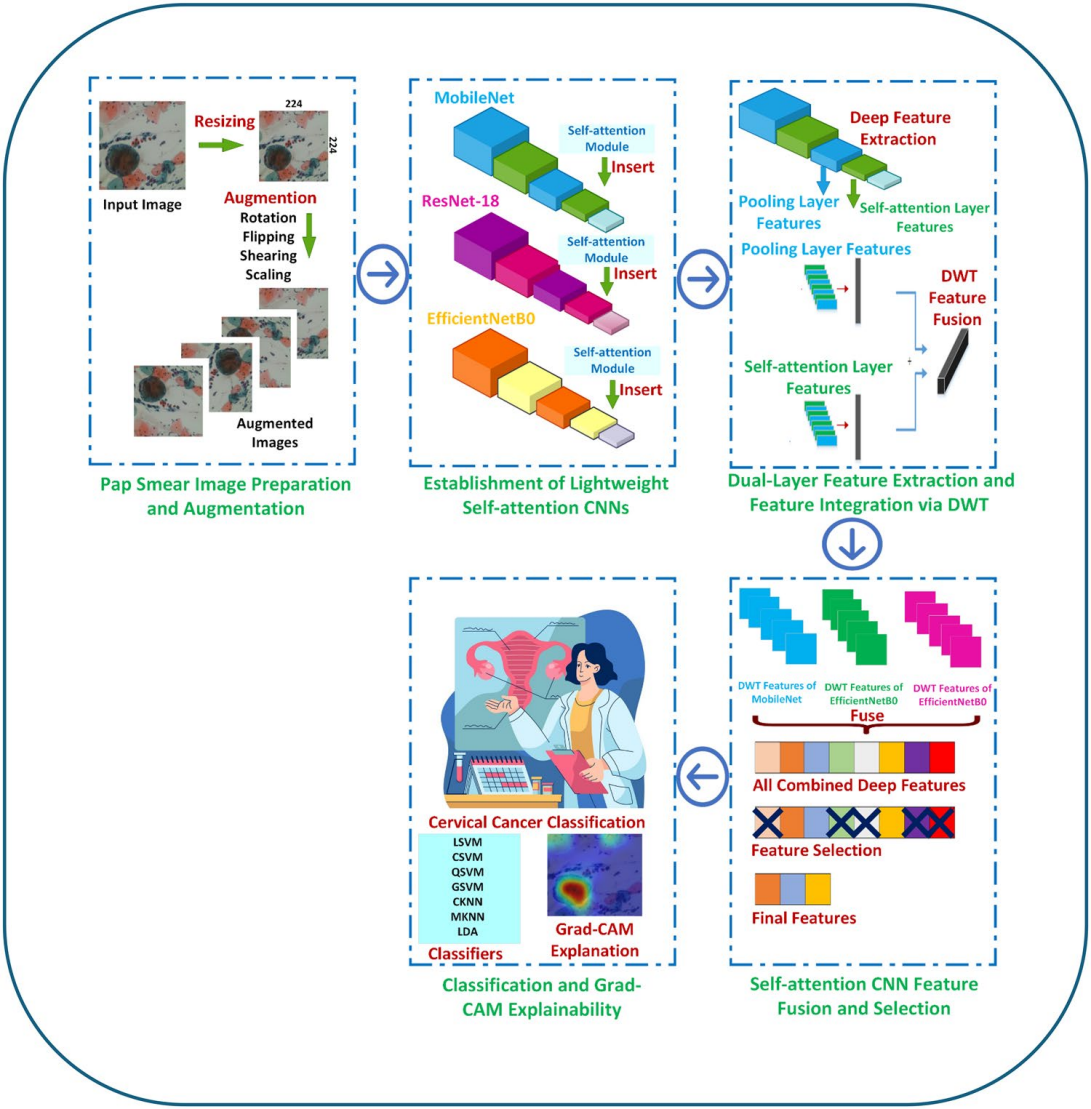
Summary of the steps of the proposed Light-XAI CADx

The three self-attention CNNs’ features are then aggregated to produce a whole representation of the images during the self-attention CNN feature fusion and selection phase. Using the strengths of every CNN, this fusion stage could improve the classification performance. Following an analysis of variance (ANOVA) feature selection technique, the best discriminative ability among the fused features is found. Reducing the dimensionality of the feature space and choosing the most instructive features will help to improve the generalisation of the model. Finally, various machine learning classifiers, including support vector machines (SVMs) with several kernels, k-nearest neighbours (KNN), and linear discriminant analysis (LDA), are employed to classify pap smear images depending on the selected criteria throughout the classification stage. The choice is based on their ability to adapt and its effectiveness in handling data with many dimensions. A summary of the steps of the proposed CADx Light-XAI is shown in Fig. 3.

#### Pap smear image preparation

The pap smear photographs from the two sets of data have been modified to conform to the dimensions necessary by the input phase of the three CNNs, which are 224 by 224 by 3. This is done to guarantee that all of the pictures are acceptable on CNN. After that, both datasets are separated into training and testing sets, with 70% of the data being used for training and 30% being used for testing. The training data are substantially enlarged through the use of a variety of augmentation approaches to alleviate the problem of overfitting and to enhance the training process. The above data augmentation methods are discussed in Table 2. The augmentation ranges presented in Table 2 were deliberately determined to represent the inherent stochasticity observed in actual cytology practice. In Pap smear imaging, cell orientation is intrinsically random due to slide preparation, smear application, and microscopic acquisition. Thus, rotations of up to ±90° do not create unrealistic structures but instead replicate natural orientation differences frequently encountered in clinical cytology. Likewise, modest reflection and translation techniques were employed to improve resilience to positional variability while preserving cellular morphology and diagnostic characteristics, including nuclear shape, size, and chromatin texture.

**Table 2:** Augmentation procedures operated and their scale values

Augmenting Methodology	Ranges
Scaling	0.6 to 2.6
Shearing at 90 degrees	−59 to 59
Reverse in ×and y orientations	−46 to 46
Spinning in ×and y orientations	−90 to 90

Furthermore, the augmentation parameters were chosen according to established methodologies in the processing of cytopathological images, where robust geometric augmentation is commonly utilised to enhance generalization in scenarios with little data. Significantly, all enhancements were exclusively geometric (rotation, reflection, translation) and excluded colour jittering, elastic deformation, or intensity warping that can damage cytological structures. This design decision protects the natural integrity of the nuclei and cytoplasm while enhancing sample variety.

In addition, data augmentation was implemented stochastically during the training of deep learning models via MATLAB’s default randomised augmentation process. Augmentations were carried out on-the-fly to the training data exclusively, with an arbitrary choice for each mini-batch, guaranteeing that no augmented examples were shared across the training and validation/test sets. The sequence and variability of changes adhered to MATLAB’s internal augmentation protocol, which is consistent for a specific software version and commonly utilised in reproducible deep learning practices. The augmentation settings were constant throughout all models, facilitating equitable assessment and mitigating augmentation-induced bias among approaches.

It is worth mentioning that the class imbalance within Mendeley LBC data was addressed using stratified splitting and training augmentation only targeted at the training dataset, while evaluation was performed on unbalanced test datasets and assessed using robust imbalance handling metrics only.

#### Lightweight self-attention CNNs establishment

In this stage, three pre-trained lightweight CNNs are modified, including EfficientNetB0, MobileNet, and ResNet-18. Initially, for each CNN, the fully connected (FC) layer, the softmax layer, and the classification layer are removed. Afterward, the self-attention module is inserted into each CNN. The insertion of the self-attention module is because cervical cytology diagnoses often rely on clinically relevant long-range spatial dependencies; therefore, the conformity of CNN architectures to local receptive fields limits their effectiveness in detecting such dependencies. In the case of Pap smears, essential diagnostic indicators—including nuclear enlargement, chromatin distribution, and nucleus-to-cytoplasm ratios—are likely to be separated spatially, and therefore, when using just stacked convolution and pooling layers, these indicators cannot be accurately represented.

In order to overcome this limitation of CNNs, the addition of a lightweight self-attention mechanism at each CNN backbone allows for the explicit modeling of non-local relationships amongst spatial features. Unlike transformers and fully attention-based networks, which introduce a profoundly increased computational load, the presented attention block works with high-level feature maps and allows for global contextual understanding and incorporation of information at a time when the additional cost will be minimal. Such a configuration permits the network to emphasise those areas that contain diagnostically relevant information whilst suppressing other nondiagnostic areas.

**Fig. 4:**
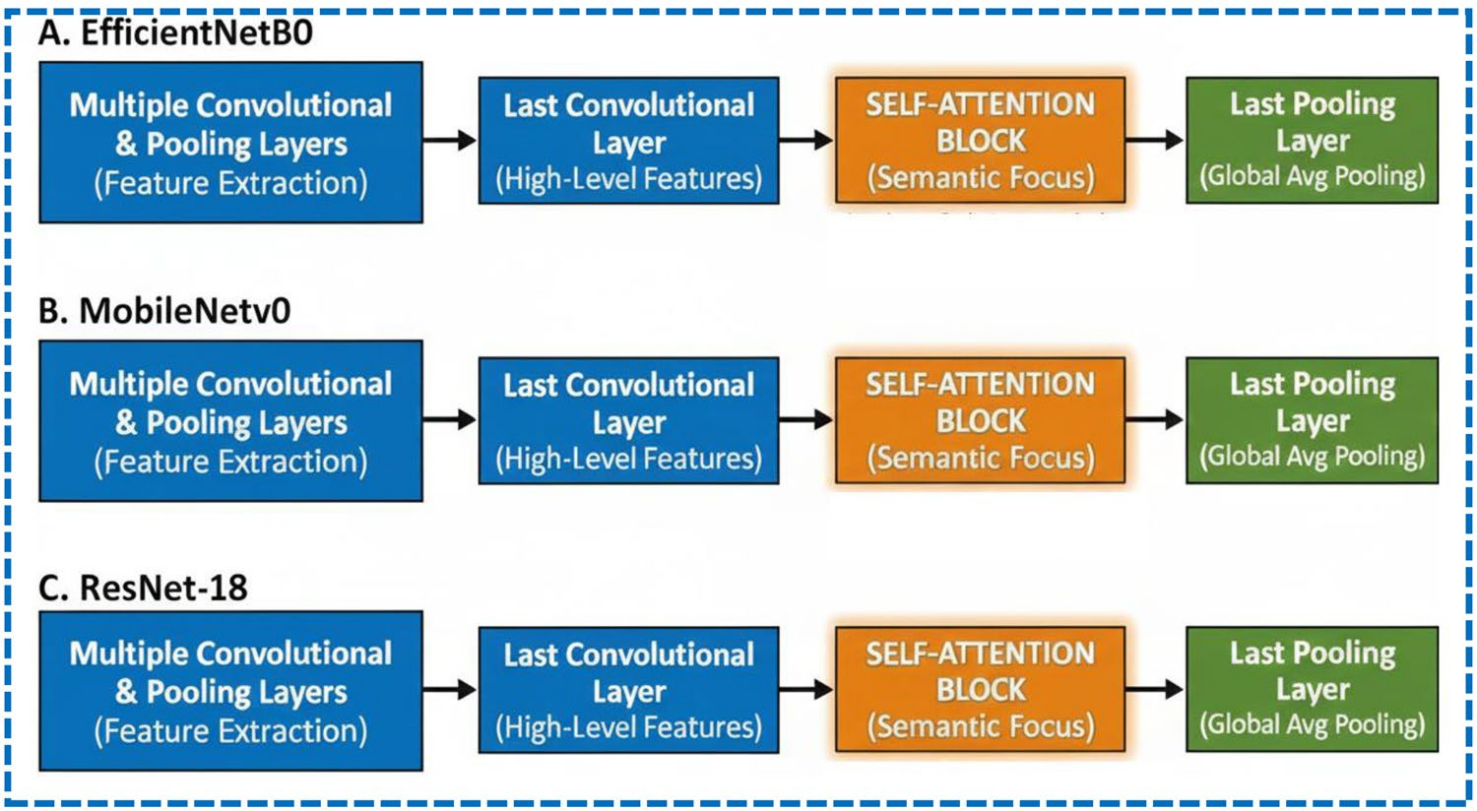
Placement of the self-attention module in each CNN

As shown in Fig. 4, the placement of the self-attention module is specifically between the last convolutional layer and the last pooling layer of each CNN, allowing it to work on the high-level semantic feature maps while minimising the additional computational load.

**Fig. 5:**
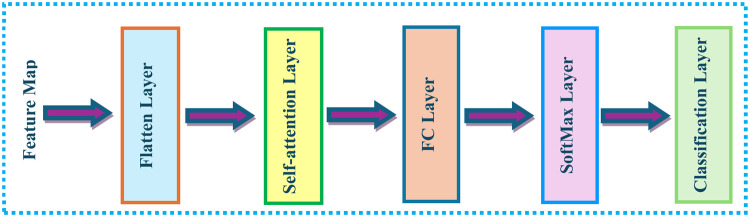
Self-attention module

The self-attention module consists of a flatten layer followed by a self-attention layer, a FC layer, a softmax layer, and finally a classification layer. Figure 5 shows the structure of the self-attention module. The number of heads for the self-attention layer is 8, while the number of channels for MobileNet and EfficientNetB0 is 1280 and 512 for ResNet-18. Next, the flatten layer is connected to the last pooling layer. Lastly, pre-processed Pap smear images are employed to train self-attention CNNs.

Given an intermediate feature map $F \in {\mathbb{R}^{H \times W \times C}}$, where *H* represents the spatial height, *W* defines the spatial width of the feature map, and *C* signifies the total amount of feature channels. *F* is reshaped to create a sequence $X \in {\mathbb{R}^{H \times C}}$, in which the length of the sequence is defined as $N= H\, W$, where each row corresponds to a spatial location in the original feature map.

The self-attention mechanism utilises three trainable linear projections:

- ${W_Q} \in {\mathbb{R}^{C \times d}}$: query projection matrix
- ${W_K} \in {\mathbb{R}^{C \times d}}$: key projection matrix
- ${W_V} \in {\mathbb{R}^{C \times d}}$: value projection matrix

The projected representations are computed as follows.

1 $$Q = X{W_Q},$$

2 $$K = X{W_K},$$

3 $$V = X{W_V},$$

where $$Q,K,V \in {\mathbb{R}^{N \times d}} $$.

For multi-head attention, the embedding dimension $is$ divided across $h$ attention heads, such that:

4 $$d= h \times d_{K},$$

Where:

- $h$ is the number of attention heads,
- ${d_k}$ is the dimensionality of each head.

For each head $i \in \left\{ {1, \ldots ,h} \right\}$, scaled dot-product attention is computed as:

5 $${\mathrm{Attentio}}{{\mathrm{n}}_i}\left( {{Q_i},{K_i},{V_i}} \right) = {\mathrm{softmax}}\,\left( {\frac{{{Q_i}K_i^ \top }}{{\sqrt {{d_k}} }}} \right){V_i}$$

The final output of the multi-head attention block is created by concatenating the multiple heads together and performing a linear projection. ${W_O} \in {\mathbb{R}^{d \times C}}$, where ${W_O}$ is the output projection matrix, yielding the final attention-enhanced representation:

6 $${\rm{SA}}\left( X \right) = {\rm{Concat}}\left( {{\rm{Attentio}}{{\rm{n}}_1}, \ldots ,{\rm{Attentio}}{{\rm{n}}_h}} \right){W_O}.$$

To achieve both representational expressiveness and computational efficiency, a principled design decision was made for the proposed Light-XAI framework with regard to how many attention heads and what dimension to use as the embedding for attention. The design choices for the attention were made to allow for enough expressiveness for discriminative cytological patterns in each attention head’s representation while limiting the memory and computational latency that arise from the increased number of heads with larger multi-head attentional architectures (e.g., those used in transformers). Because of the compactness of the feature maps generated by CNNs in this framework (versus the long sequences of tokens used in transformers), an excessive number of heads is not required, and the design focuses on improving already informative representations rather than modeling the entirety of the information globally.

Additionally, the particular embeddings chosen for the attention also correspond to the channel dimensions of the backbone feature maps, serving to make the attention blocks function as a lightweight contextual re-weighting mechanism as opposed to being the major computational component. This design preserves spatial information, allows for better performance due to the limited sample sizes typically associated with Pap smear datasets, and reduces the risk of overfitting.

The use of lightweight attention mechanisms with fewer heads has become common practice in hybrid CNN-Attention approaches to analysing medical images. Because attention is used in these instances to highlight areas within the image that are important for diagnosis (rather than modelling long-range dependences), any increase in headcounts or embeddings provides limited return and raises extended computational complexity and memory costs. This goes against the goal of lightweight architecture.

Accordingly, the configuration of attention used in this study represents a conscious and cost-effective decision, indicating that its intended role in the architecture is to enhance the focus of discrimination and enhance explainability while not sacrificing the model’s size or generalisation capacity.

The proposed Light-XAI system integrates EfficientNetB0, MobileNet, and ResNet-18 to take advantage of their mutual strengths and design benefits, as each model provides particular benefits that enhance feature extraction and classification in the diagnosis of cervical cancer. EfficientNetB0 is notably beneficial due to its compound scaling technique, which balances the network’s depth, width, and resolution to obtain excellent accuracy with a minimal number of parameters, leading to improved performance compared to arbitrarily scaling [83]. This efficient construction enables the capture of intricate and multiscale attributes within the input data, which is particularly advantageous for medical imaging applications requiring precise identification of detailed patterns. EfficientNetB0 enhances Light-XAI’s objective of achieving high accuracy while reducing processing requirements through efficiency optimisation without compromising performance.

In contrast, MobileNet is recognised for its computational effectiveness and is tailored for applications necessitating minimal latency and lightweight structures. It employs depth-wise separable convolutions that substantially decrease the computational cost and model dimension while preserving good accuracy. Separable deep-wise convolutions decompose the ordinary convolution into a deep-wise convolution and a point-wise convolution, thus lowering computational requirements [84]. By integrating MobileNet, Light-XAI can deliver a solution that is both precise and suitable for resource-limited environments, such as mobile or embedded medical equipment.

Ultimately, ResNet-18 offers the benefit of residual connections, alleviating the problems associated with vanishing gradients in deeper networks [85]. This feature enables ResNet-18 to retain superior performance in capturing spatial information, an important feature for medical image analysis, when differentiating minor morphological characteristics is critical. The residual architecture of ResNet-18 helps to maintain and highlight vital features throughout the network layers, therefore facilitating the classification of complex cell patterns found in images of cervical cancer. The model’s superficial construction renders it especially appropriate for medical image processing tasks with potentially constrained dataset sizes.

The reason these three architectures are combined instead of relying on a single backbone is that cervical cytology images have a variety of different visual characteristics that cannot be captured using just one network. EfficientNetB0 focuses on aggregating multiscale features; MobileNet uses a texture-efficient method for representing textures; and ResNet-18 maintains spatial details by using a residual learning concept. Light-XAI fuses these complementary types of representations to reduce the architecture’s inherent biases and increase the system’s robustness to variations in staining, morphological diversity, and acquisition differences, which are frequently seen in Pap smear images. Therefore, this method of integrating these three architectures serves to enhance the ability to generalize across a wide range of cervical cytological images while still having low computational requirements.

#### Dual deep-layer feature extraction and integration

In this stage, after completion of the training phase of each CNN, transfer learning is adopted to obtain deep features from each CNN. The purpose of the stage is to extract deep features from the two distinct layers of each CNN of self-attention, as opposed to obtaining deep variables from a single layer of a self-attention CNN. The deep features are acquired from the last pooling layer and the self-attention layer of each self-attention CNN. The rationale behind using both of these layers to extract features is that they provide complementary and non-redundant information. The features obtained from the pooling layers will provide a compactly aggregated representation of the most important morphological characteristics and high-level structural information within the Pap smear images, including the overall shape of the cells and the distribution of coarse textures. Whereas the features obtained from the self-attention layers will contain contextual and relational information about the images, highlighting areas of high discriminative power and long-distance relationships between the nuclear and cytoplasmic borders of cells.

The proposed model combines these two types of representations or features to allow for a combination of global morphological information (taken from pooling layers) with local, attention-weighted discriminative information (taken from self-attention layers). This combination is a key aspect of the proposed methodology for cervical cytology, as many cells show patterns that span multiple spatial dimensions and cannot be effectively represented by either type of feature alone.

Within the proposed Light-XAI framework, deep features extracted from the two-layer (pooling and self-attention) of each CNN are concatenated to generate one deep feature representation for each of the three networks, which are then input to the DWT. Using the concatenated feature vectors, the DWT is applied through two decomposition levels. The DWT breaks down the input data into a collection of wavelet coefficients by subjecting it to high-pass and low-pass filters at various scales. Upon entering DWT, the input data are initially separated into approximation (CA) and detail coefficients (CD) at the lowest level of resolution. The CA represents the broader pattern of the data, while the CD contains specific information about the high-frequency components. Subsequently, this procedure is repeated on the CA coefficients, breaking them down into smaller scales with increasingly intricate data in the detail coefficients. This hierarchical decomposition enables analysis of both the frequency content and the temporal occurrence of the signal [86].

A two-level decomposition design is chosen in order to capture the multiresolution characteristics of the fused deep features without introducing additional redundancy. One level would not provide enough abstracted representation of the fused deep feature representation, while three or more would provide excessive oversmoothing and increased computation costs, with no relative benefits, especially when representing already abstracted deep features. This selectivity aims to balance representational richness with computational efficiency; it is not a random decision.

In order to maintain a compact and robust representation of features, only the CA coefficients are kept from each decomposition level. These coefficients denote the low-frequency, information-dense elements of the integrated deep feature space and are used to create the final reduced set of features, which will be used for feature selection/classification in a later step. The reason for retaining only the CA coefficients is that they capture the structural and statistical characteristics of the overall geometry of the combined deep feature space while effectively eliminating relatively large amounts of high-frequency components (noise) or low-level variations between deep features that create redundancy. Given the fact that the DWT will utilise high-level features (deep features) rather than raw pixel intensity values as input, the CD will typically, when included, provide little incremental benefit from a discriminative standpoint and will only increase the dimensionality and sensitivity to noise. By retaining only the CA coefficients, Light-XAI ultimately has a smooth, packed, and stable representation of a feature space, leading to a greater potential for generalisation, reducing overfitting effects, and helping to better mitigate the typical data sizes used in cervical cytology studies.

The DWT provides accurate time resolution because its wavelet basis functions are concentrated in both the time and frequency domains. This enables the capture of temporary characteristics and the identification of time points at which specific frequencies occur within the signal [87]. Moreover, the DWT can be employed to reduce feature length. Through the examination of the energy distribution among the wavelet coefficients, it is possible to identify coefficients that possess a minimal amount of information. These coefficients can be safely discarded without significantly impacting the overall signal representation. This process efficiently decreases the number of dimensions in the data, while retaining the most informative elements [88]. In this study, the ‘Haar’ mother wavelet is employed, and two decomposition levels are applied to the data. The lengths of deep features after and before DWT integration are shown in Table 3. The selection of the Haar wavelet was driven by its low complexity, orthogonality, and robust localisation capabilities, rendering it especially appropriate for biomedical image analysis and texture-based representation. Haar wavelets proficiently detect sudden intensity variations and edge-like formations, prevalent in cervical cytology images, particularly at nuclear borders and cytoplasmic transitions. Furthermore, Haar wavelets are computationally effective and have no overhead, consistent with the conceptual framework of Light-XAI, which emphasises efficiency and resilience rather than unnecessary parameterisation. This selection aligns with previous biological signal and image processing research, where Haar wavelets are commonly utilised for feature improvement when interpretation and computational efficiency are essential.

**Table 3:** Length of deep features mined from the two layers of self-attention CNNs before and after the DWT integration step

CNN Structure	Pooling Layer	Self-attention Layer	DWT Integration Level 1	DWT Integration Level 2
ResNet-18	512	512	512	256
MobileNet	1280	1280	1280	640
EfficientNetB0	1280	1280	1280	640

Standard DWT is chosen in this study instead of dual tree DWT (DT-DWT) as DWT provided the optimal balance of performance, efficiency, and interpretability when the goal of feature fusion and dimensionality reduction of CNN-derived representations was required. The additional directional redundancy and computational expense associated with DT-DWT provide little in the way of practical benefit.

#### Self-attention CNN feature fusion and selection

The integrated DWT deep features obtained in the previous step from each self-attention CNN are further fused in a concatenated manner. This fusion process results in a massive dimension of features; thus, feature selection is essential. Feature selection is a collection of techniques that aim to identify significant attributes while eliminating redundant or irrelevant ones, thus decreasing the degree of difficulty of classification tasks and avoiding overfitting. In this research, the ANOVA feature selection is adopted. ANOVA defines each feature’s discriminative power and ranks them according to their ratio of variance (between-class to within-class). By identifying the most influential features for class separability. Features with a high *p*-value and demonstrated negligible variations are removed, while variables with a low *p*-value and showed significant differences between classes are considered more useful for classification and are retained [89]. ANOVA provides a suitable method for multiclass cervical cytology classification, which requires very slight differences in the morphology of cells to be consistently identified across each class. ANOVA provides a statistical interpretation of the feature results, unlike heuristic dimensionality reduction techniques or end-to-end pruning strategies. Using ANOVA allows for the explicit measurement of class-discriminative significance and ease of transparent ranking of the deep features selected based on statistical evidence. This method directly supports the study’s focus on explainable and consistent results; there is a clear connection between the ANOVA method and its statistically confirmed class differences, instead of the more obscure mathematical dynamics involved with optimising features.

In addition, ANOVA is highly efficient from a computational standpoint and is classifier agnostic, which is ideal for integration into a lightweight CADx workflow. When used in conjunction with repeated cross-validation and subsequently the statistical validation of results, ANOVA allows for a more stable feature set with reduced variances related to random initializations and random partitions due to the limited sample sizes in medical imaging.

#### Classification

Seven classifiers are applied to categorise the two Pap smear datasets. Among such classification models are linear support vector machine (LSVM), quadratic SVM (QSVM), cubic SVM (CSVM), Gaussian SVM (GSVM), K-nearest neighbour with Euclidean distance metric (MKNN), K-nearest neighbour with Cosine distance metric (CKNN) and linear discriminant analysis (LDA). Four different settings are used throughout the classification process. End-to-end classification is accomplished for every CNN after the self-attention module. This is demonstrated in the first setting. The second scenario shows classification with deep features taken from each of the two layers of each CNN individually. The classification using every integrated DWT set of deep attributes forms the third setting. The combined attributes derived from both layers of all three CNNs are entered into the Feature Selection (FS) approach within the fourth setting. The classifiers are next fed with the selected features. Stratified five-fold cross-validation was utilized with MATLAB, which inherently maintains class distribution across folds for classification problems to assess the performance of Light-XAI in classification tasks and to deal with the class imbalance problem available in the Mendeley LBC dataset.

## Experiments adjustment

The following part provides the evaluation indicators employed for assessing the performance of Light-XAI, along with the optimised hyperparameter configurations. Every CNN has had multiple of its hyperparameter values changed. Table 4 lists the hyperparameters together with the related values. All other configurations are kept at their initial settings. All CNNs were trained with stochastic gradient descent with momentum (SGDM) to achieve stable optimisation, using a fixed learning rate of 0.001, a momentum of 0.9, and an L2 weight decay of 0.0001 to reduce the likelihood of overfitting. Each CNN model was trained for 50 epochs using mini-batches of size 5 and did not utilise any learning-rate scheduler to maintain the same optimisation dynamics for all models. The frequency of validation of the models was determined based on the size of the respective datasets, with validation occurring every 168 iterations for the SIPaKMeD dataset and every 708 iterations for the Mendeley LBC dataset. The self-attention setup remained constant across all CNN models to isolate the impact of the suggested feature fusion technique and prevent misleading architectural heterogeneity. Although comprehensive hyperparameter adjustment of attention heads is feasible, it was deliberately not undertaken in this work to maintain a lightweight design philosophy and guarantee equitable comparison among backbones.

**Table 4:** Hyper-parameters fine-tuning of the self-attention CNNs

Hyper-parameter	Value
Mini batch	5
Epochs	50
Learning rate	0.001
Learning rate scheduling	none
Optimizer	Stochastic gradient descent with momentum
Optimizer momentum	0.9
Weight decay (L2 Regularization)	0.0001
Validation frequency	168 708
Mendeley LBC dataset	
SIPaKMeD dataset	

In Settings II and III, all classifiers were developed and assessed using similar setups across datasets to guarantee an equitable and regulated comparison. Four kernel types were evaluated for the SVM classification algorithms: linear (LSVM), quadratic (QSVM), cubic (CSVM), and Gaussian (GSVM). In each scenario, kernel scaling and box constraint parameters were established using MATLAB’s default automated optimisation, which adjusts these values according to the data distribution. Feature standardisation was implemented to normalise the input space before classification and enhance numerical stability.

Both medium K-Nearest Neighbors (MKNN) and cosine K-Nearest Neighbors (CKNN) topologies were assessed for KNN categorization. Both KNN models utilised MATLAB’s default neighbor count (k = 10). The similarity measure employed was the Euclidean distance with uniform distance weighting, and the standardization was included for MKNN, while the cosine similarity measure was used for CKNN. A linear discriminant approach was employed for LDA with MATLAB’s default regularisation parameters. Feature standardisation was implemented to guarantee uniform scaling across dimensions. All machine learning classifiers’ hyperparameters were configured to the specified standard settings. Identical configurations were uniformly implemented throughout all tests to provide robustness, reproducibility, and equitable comparison among classifiers.

Although nested cross-validation is considered to be the most rigorous way of performing unbiased hyperparameter optimisation, the hyperparameters utilised in this study were predetermined based on previous studies of hyperparameter settings for CNN architectures and classifiers as reported in the literature and detailed in the manuscript tables. The aim was to compare the efficiency and explainability of different architectures using the same set of hyperparameters, not to determine which would have the best performance through exhaustive tuning.

To account for the variance introduced by randomisation in data partitioning, the repeated five-fold cross-validation was used in combination with means, variances and statistical significance tests to create an overall robust and fair evaluation structure.

The CNN models that utilized the self-attention module (i.e., EfficientNetB0, MobileNet, and ResNet-18) were trained using categorical cross-entropy as the loss function, which is the standard and most appropriate loss function for multiclass image classification tasks.

As an example, let $y = \left[ {{y_1},{y_2}, \ldots ,{y_C}} \right]$, where *y* reflects C classes in the form of an encoded vector, and let $\hat y = \left[ {{{\hat y}_1},{{\hat y}_2}, \ldots ,{{\hat y}_C}} \right]$, denote the predicted class probabilities obtained after the softmax layer. Categorical cross-entropy loss is calculated by using the following equation:

7 $${\mathcal{L}_{CE}} = - \mathop \sum \limits_{c = 1}^C {y_c}\log \left( {{{\hat y}_c}} \right)$$

According to this definition, this loss function will reward and thus reinforce the model’s prediction probability for the correct class, as well as providing a penalty for incorrect predictions. To determine the optimal value of the loss function, a stochastic gradient descent with momentum method was utilised

Light-XAI experiments were conducted using MATLAB 2023a. The deep learning, computer vision, statistics, and machine learning toolboxes were used to process images, construct the self-attention CNNs and Grad-CAM, and build the classification models. The system was equipped with an Intel(R) Core (TM) i7-10750 H processor, an NVIDIA GeForce GTX 1660 video controller with 6 GB of memory, and a 64-bit operating system.

The efficacy of Light-XAI is thoroughly assessed using a range of diagnostic metrics that offer an in-depth evaluation of its predictive abilities. This analysis encompasses standard classification performance metrics, including the F1-score, accuracy, specificity, precision, Matthews correlation coefficient (MCC), sensitivity, and receiver operating characteristic (ROC) analysis, along with the area under the ROC curve (AUC). A fundamental comprehension of essential classification indicators of the results is vital for accurately calculating these metrics. The essential indicators consist of four principal categories: true positive (TP), true negative (TN), false positive (FP), and false negative (FN) outcomes. TP denotes the quantity of positive instances correctly recognised by the model, whereas TN reflects the number of negative examples successfully identified. Inversely, false positives (FP) arise when the model incorrectly categorises negative data points as positive, while false negatives (FN) denote positive cases that are inaccurately classified as negative. These metrics are determined by utilising these equations:

8 $$Sensitivity = \frac{{TP}}{{TP + FN}}$$

9 $$Specificity = \frac{{TN}}{{TN + FP}}$$

10 $$Precision = \frac{{TP}}{{TP + FP}}$$

11 $$MCC = \frac{{TP \times TN - FP \times FN}}{{\sqrt {\left( {TP + FP} \right)\left( {TP + FN} \right)\left( {TN + FP} \right)\left( {TN + FN} \right)} }}$$

12 $$F1 - measure = \frac{{2 \times TP}}{{\left( {2 \times TP} \right) + FP + FN}}$$

13 $$Accuracy = \frac{{TP + TN}}{{TN + FP + FN + TP}}$$

## Experimental results

The classification process is carried out in four distinct settings. After inserting the self-attention module, end-to-end classification is achieved for each CNN. This is demonstrated in the initial setting. In the second setting, classification is demonstrated by extracting deep features specifically from each of the two layers of each CNN on an individual basis. The third setting corresponds to the classification that makes use of each integrated DWT deep feature set. In the fourth context, the combined features that have been collected from both layers of the triple CNNs are fed into the FS technique. After this, the selected features are used to feed the classifiers. This section illustrates the results of these four settings.

### Setting I classification results

This section describes the result of the end-to-end deep learning classification for the three CNNs before and after adding the self-attention module. The classification accuracies of the three lightweight CNNs before and after adding the self-attention module are shown in Table 5. The purpose of the self-attention module is to enhance the network’s capacity to concentrate on identifying significant areas of the input image for the purpose of classification. Following the incorporation of the self-attention module, each of the three CNNs exhibits substantial enhancements in accuracy when their performance is evaluated on the Mendeley LBC dataset. The greatest boost is demonstrated by EfficientNet, as its accuracy rises from 82.35% to 83.39%. Moving from 87.54% to 88.93%, MobileNet also demonstrates a significant increase in its performance. ResNet-18 shows a more moderate magnitude increase, from 84.43% to 85.81%. As a result of these findings, it appears that the self-attention module is successful in capturing long-range dependencies within the data. This resulted in a greater understanding of the image features and improved classification performance for the Mendeley LBC data set.

**Table 5:** Classification accuracy (%) of lightweight CNNs before and after the addition of the self-attention module

Model	Without the Self-attention Module	With the Self-attention Module
SIPaKMeD dataset		
EfficientNetB0	57.41	58.32
MobileNet	58.90	58.15
ResNet-18	57.08	60.21
Mendeley LBC dataset		
EfficientNetB0	82.35	83.39
MobileNet	87.54	88.93
ResNet-18	84.43	85.81

Nevertheless, the findings for the SIPaKMeD dataset depict an alternate scenario from the previous one. Compared to the Mendeley LBC dataset, the accuracy enhancements in this case are lower. The accuracy of EfficientNet increased from 57.41% to 58.32%, the accuracy of ResNet-18 increased from 57.08% to 58.08%, and the accuracy of MobileNet decreased slightly from 58.90% to 58.15%. These variations in accuracy for the SIPaKMeD dataset demonstrate that lightweight CNNs, even with the addition of the self-attention module, had difficulty learning discriminating attributes as a result of this dataset.

It can be seen in Table 5 that the introduction of the self-attention module greatly improved the classification performance of the three lightweight CNNs when evaluated on the Mendeley LBC dataset. This enhancement is remarkable for EfficientNetB0, ResNet-18, and MobileNet, which achieved significant improvements in accuracy. The accuracy enhancement can be attributed to the self-attention module’s capacity to recognise long-term dependencies in the data, which allowed the models to gain a deeper understanding of the intricate connections among image features. Nevertheless, the influence of the self-attention module on the SIPaKMeD database was rather muted. Although improvements were seen in EfficientNetB0 and ResNet-18, MobileNet showed a modest decline in accuracy. These differences can be ascribed to multiple contributing factors, including the fact that the SIPaKMeD dataset may have lower image quality compared to the Mendeley LBC dataset. The results emphasise the need to take into account the characteristics of the dataset while developing and evaluating deep learning models for the analysis of medical images. Although the self-attention module demonstrated efficacy on the Mendeley LBC dataset, its efficacy may differ on other datasets with distinct characteristics. The subsequent experiment investigates the use of transfer learning to enhance performance. This is done because of the limitations observed with the SIPaKMeD dataset. For this experiment, seven different machine learning classifiers are trained using features that have been extracted from a CNN that had already been trained. Training and validation loss plots for each of the end-to-end CNN models are provided in the Supplementary Materials (Figures S0.1 and S0.2, for the SIPaKMeD dataset and the Mendeley LBC dataset). These loss curves illustrate that there was consistent and stable convergence behaviour throughout the training epochs, without displaying any signs of an unstable optimisation process (instability) or overfitting. This information corroborates the meaningfulness of the results that are presented, confirming stable convergence and the absence of overfitting in Setting I.

### Setting II classification results

The classification results of machine learning classifiers trained with each deep layer feature set independently are shown in Tables 6 and 7 for the SIPaKMeD dataset and the LBC Mendely dataset, respectively. Table 6 indicates that the deep features obtained from both the self-attention and the pooling layers have comparable performance for the three self-attention CNNs. Table 6 reveals that the deep features obtained from the self-attention and pooling layers of EfficientNetB0, MobileNet, and ResNet-18 exhibit similar performance across different classifiers, including LSVM, QSVM, CSVM, MSVM, MKNN, CKNN, and LDA. The small disparities in classification accuracy observed between the self-attention and pooling layers, often falling within a range of 1%, indicate that both layers effectively capture comparable meaningful attributes for the classification of cervical cancer. The consistent performance observed in different network designs and classifiers demonstrates the resilience of the proposed methodology. Significantly, QSVM, CSVM, and MSVM classifiers consistently achieve slightly better accuracies compared to other classifiers, with ResNet-18 features that typically demonstrate the most optimal performance. These results indicate that self-attention mechanisms efficiently identify pertinent features for cervical cancer classification, achieving performance comparable to conventional pooling methods. Furthermore, the decision between self-attention and grouping layers does not have an important influence on the efficiency of the classification in this particular situation. Furthermore, more intricate SVM variants (QSVM, CSVM, MSVM) seem to be especially suitable for this task. Finally, the deep features of the three CNNs, when combined with these SVM classifiers, provide an improvement in classification quality. These results emphasise the adaptability of deep learning methods in extracting significant characteristics for the classification of cervical cancer and the possibility for further improvement by a thorough selection of feature extraction techniques and classifiers.

**Table 6:** Classification accuracy (%) achieved using the classifiers trained with features extracted for the two individual layers of self-attention CNNs using the SIPaKMeD dataset

Model	LSVM	QSVM	CSVM	MSVM	MKNN	CKNN	LDA
EfficientNetB0							
Self-attention Layer	94.5	95.2	95.0	95.1	94.4	95.0	93.1
Pooling Layer	94.4	95.1	95.4	94.9	93.2	94.5	93.0
MobileNet							
Self-attention Layer	93.8	94.5	94.6	94.7	93.5	93.7	93.7
Pooling Layer	94.3	94.8	94.9	94.3	93.4	94.0	93.7
ResNet-18							
Self-attention Layer	94.4	95.2	95.3	95.5	94.9	94.6	94.0
Pooling Layer	94.8	95.5	95.5	95.5	94.8	94.7	94.2

**Table 7:** Classification accuracy (%) achieved using the classifiers trained with features extracted for the two individual layers of the self-attention CNNs using the LBC Mendely dataset

Model	LSVM	QSVM	CSVM	GSVM	MKNN	CKNN	LDA
EfficientNetB0							
Self-attention Layer	97.4	97.7	97.8	97.2	94.6	94.5	95.6
Pooling Layer	98.6	98.9	99.0	96.9	90.7	97.2	96.8
MobileNet							
Self-attention Layer	98.5	98.6	98.6	98.8	95.8	97.3	96.6
Pooling Layer	98.0	98.1	97.9	97.3	92.9	95.0	96.4
ResNet-18							
Self-attention Layer	97.8	98.3	98.3	98.2	94.8	96.8	97.7
Pooling Layer	98.4	98.5	98.8	98.8	96.7	96.3	98.1

On the other hand, Table 7 demonstrates that the deep features of the two layers have different accuracy. For example, in the case of EfficientNetB0, the deep features of the pooling layer attained a higher accuracy for L-SVM, Q-SVM, C-KNN, and LDA compared to the deep features of the self-attention layer, and a lower accuracy using G-SVM and M-SVM compared to the deep features of the self-attention layer. Likewise, for ResNet-18 deep features, the pooling layer achieves greater accuracy than the self-attention layer for L-SVM, Q-SVM, C-SVM (98.8%,98.3%), G-SVM, M-KNN and LDA except for C-KNN. Conversely, for MobileNet deep features, the self-attention layer reaches superior classification accuracy than the pooling layer for all classifiers.

An investigation of deep features obtained from self-attention and pooling layers in several architectures (EfficientNetB0, ResNet-18, and MobileNet) shows notable patterns in classification precision, as noted in Table 7. The pooling layer features of EfficientNetB0 and ResNet-18 typically exhibit superior performance compared to the self-attention layer in most classifiers, with a few significant exceptions. This observation implies that the pooling layer may be more efficient in capturing distinguishing characteristics for the classification of cervical cancer in these particular architectures. Nevertheless, the exceptional efficacy of the characteristics of the self-attention layer when combined with specific classifiers (such as GSVM and MSVM for EfficientNetB0 and CKNN for ResNet-18) suggests that the self-attention mechanism can provide significant additional information. In particular, MobileNet demonstrates an opposite pattern, as self-attention layer features consistently surpass pooling layer features in performance across all classifiers. This finding emphasises how feature effectiveness varies depending on the CNN structure and raises the possibility that MobileNet’s self-attention mechanism is especially appropriate for identifying related patterns in pictures of cervical cancer. These results highlight the need to precisely choose both the feature extraction layer and the classifier during the development of deep learning models for medical image analysis. The ideal combination of these two components may differ based on the particular topology and characteristics of the dataset.

### Setting III classification results

**Fig. 6:**
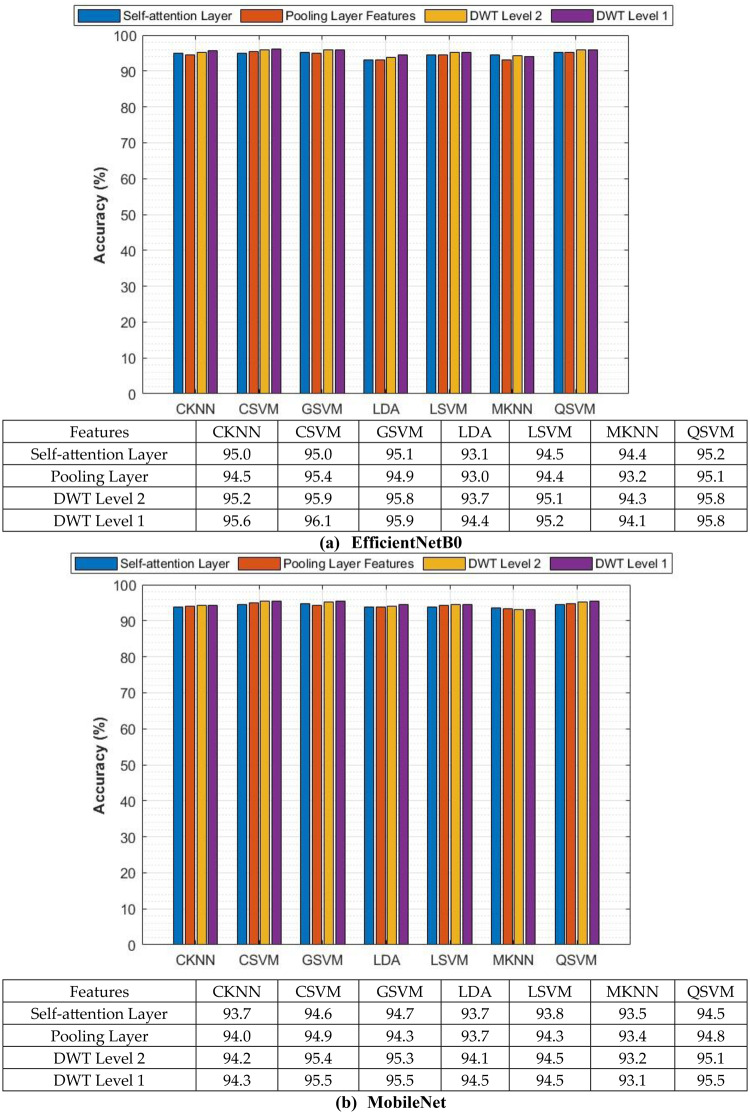

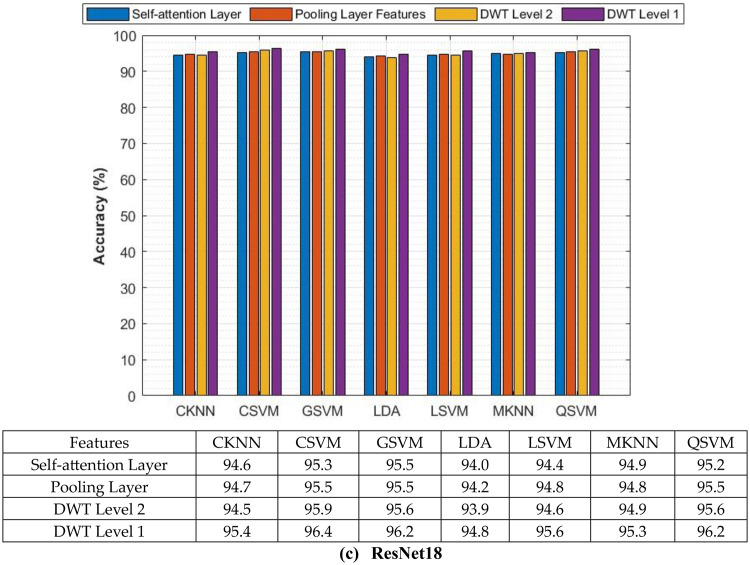
Classification accuracy (%) employing features extracted for individual and combined layers of the CNNs using two DWT decomposition levels of DWT for the SIPaKMeD dataset; (**a**) EfficientNetB0, (**b**) MobileNet, (**c**) ResNet18

**Fig. 7:**
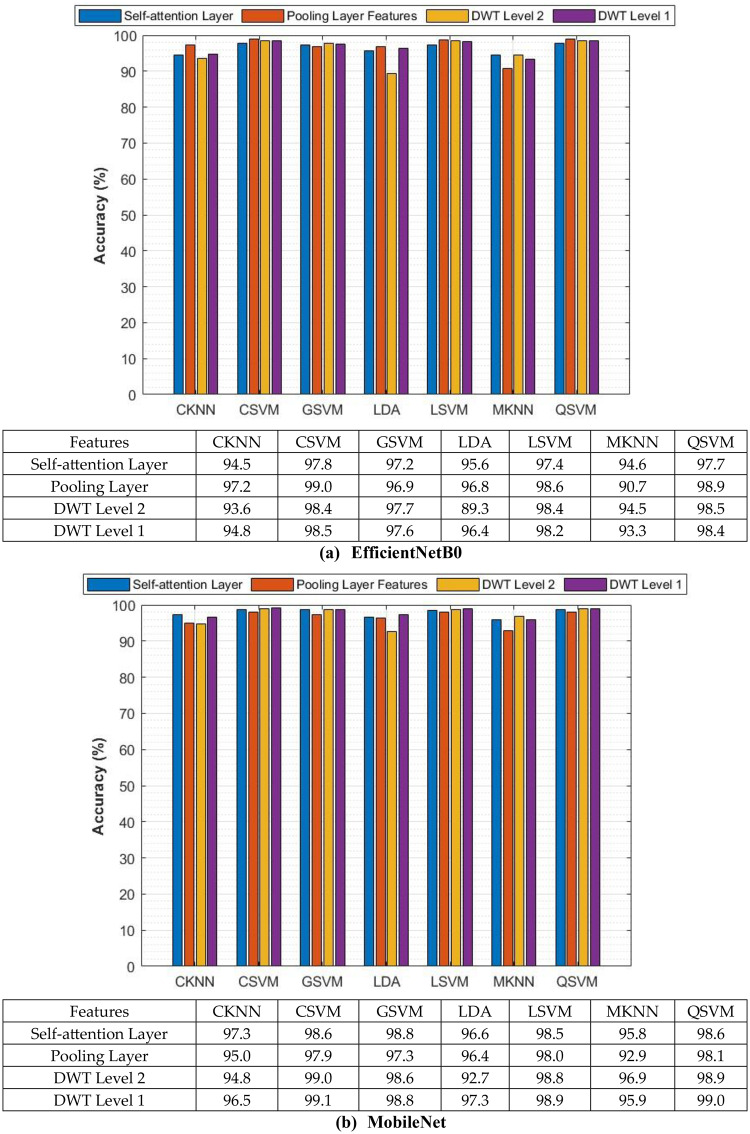

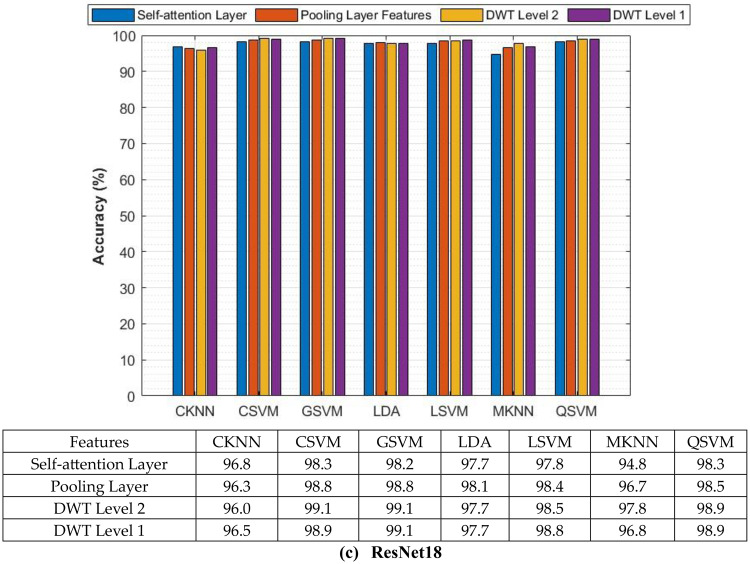
Classification accuracy (%) employing features extracted for individual and combined layers of the CNNs using two DWT decomposition levels of DWT for the Mendeley LBC dataset; (**a**) EfficientNetB0, (**b**) MobileNet, (**c**) ResNet18

The results after fusion of the two deep feature sets obtained from the pooling and self-attention layers of each CNN are displayed and discussed in this section. Figures 6 and 7 show a comparison of the accuracy achieved using the seven machine learning models constructed with each of the separate sets of deep attributes of the pooling and self-attention layers of each CNN, compared to the fused variables using the first and second decomposition levels of DWT using the SIPaKMeD dataset, and the LBC Mendely dataset, respectively. Although the use of features derived exclusively by CNNs (pooling or self-attention layers) does exhibit a few successes, the inclusion of the DWT method to fuse deep features of both layers appears to result in an overall increase in classification accuracy throughout the three CNN structures (EfficientNetB0, MobileNet, ResNet18). This is obviously demonstrated in Figs. 6 and 7. For example, Figure 6 demonstrates that the inclusion of DWT-fused features substantially improves the classification accuracy of each of the three CNN structures (EfficientNetB0, MobileNet and ResNet18) in comparison with employing features only from the pooling or self-attention layers. Specifically, the SIPaKMeD dataset reveals that the first and second levels of DWT greatly improve the performance of most classifiers, excluding MKNN when applied to EfficientNetB0 and MobileNet.These findings illustrate the efficacy of DWT in capturing additional details from deep features, resulting in enhanced classification performance.

Similarly, in Fig. 7, the Mendeley LBC dataset shows that the first and second levels of DWT have enhanced the classification accuracy for all classifiers. The GSVM classifier achieves the highest accuracies of 99.1% and 99.1% when trained with the first and second DWT features of ResNet18. The accuracy obtained surpasses those achieved by the self-attention and pooling layers. Comparable enhancements are noted for other classifiers. Furthermore, after DWT fusion, the accuracy range for LSVM in MobileNet improved from 98.5% to 98.9%, while for QSVM, it grew from 98.6% to 99.0%. Similar improvements are observed in other classifiers. Moreover, after DWT fusion, the accuracy limit for LSVM in ResNet-18 increased from 97.8% to 98.8%. Equivalent improvements are observed across different classifiers. The outcomes explicitly indicate that the features have a beneficial effect on the accuracy of the classification, emphasising the suitability of the suggested Light-XAI for the classification of cervical cancer. This indicates that DWT has the ability to assist in the extraction of useful knowledge from image input that CNNs may ignore. Furthermore, the combination of attributes from various CNN layers can provide some benefits over the use of single layers. This is due to the fact that merging features allows for the collection of understanding from various levels of abstraction within the CNN. This results in a broader comprehension of the content of the image and has the ability to improve the accuracy of the classification.

### Setting IV classification results

**Fig. 8:**
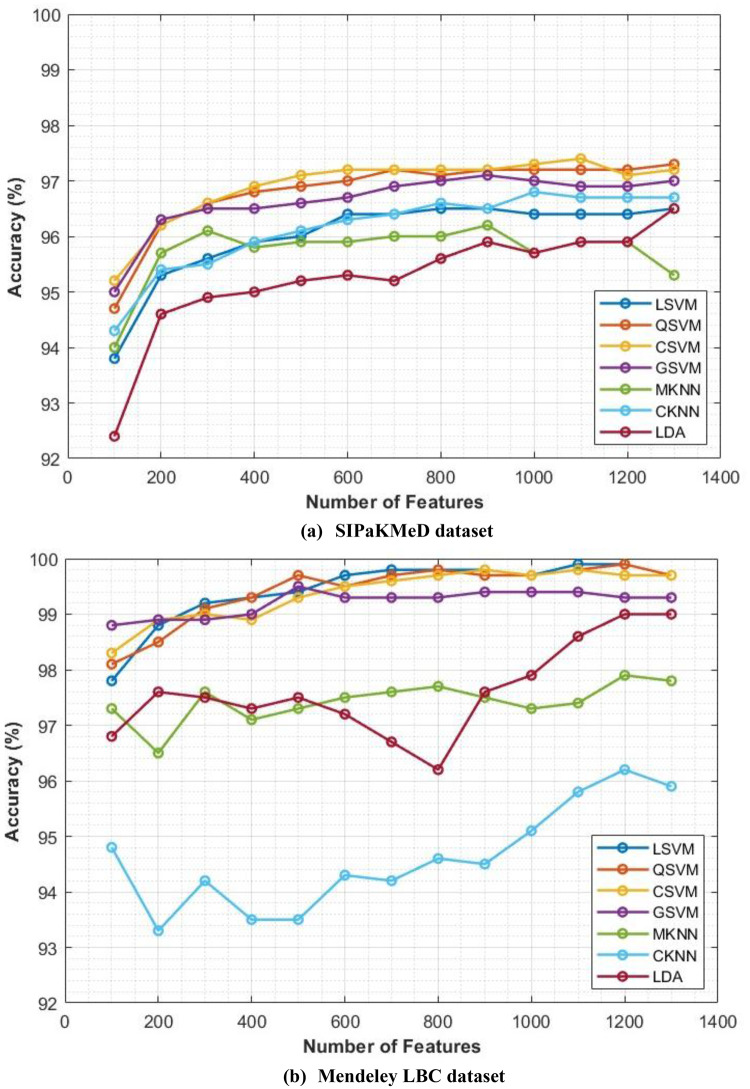
Classification accuracy (%) of the classifiers trained with the combined deep features of the three CNNs and applying ANOVA feature selection; (**a**) SIPaKMeD dataset, (**b**) Mendeley LBC dataset

This section represents the results of combining the deep features of the three CNNs and applying ANOVA feature selection. Figure 8 shows the results after the feature selection process. As can be seen in Fig. 8, the classification accuracy increases with increasing number of features until a specific point where the accuracy is either steady or decreasing. For the LSVM, QSVM, CSVM, GSVM, MKNN, CKNN, and LDA classifiers, the maximum accuracy of 96.5%,97.3%, 97.4%, 97.1%,96.2%, 96.8%, and 96.5% using 800, 1300, 1100, 900, 900, 1000, and 900 features. These accuracies are better than those achieved in setting III (Fig. 6). Similarly, the LSVM, QSVM, CSVM, GSVM, MKNN, CKNN, and LDA classifiers attain an accuracy of 99.9%, 99.9%, 99.8%, 99.5%, 97.9%, 96.2%, and 99.0% with features 11,001,200, 900, 500, 1200, 1200, and 1200, respectively. For the SIPaKMeD dataset, it can be noted from Figure 8 that the optimum number of attributes differs between the various classifiers. For instance, GSVM, MKNN, and LDA attain their maximum accuracies in the presence of 900 features, whereas CSVM and QSVM demonstrate optimal performance with 1300 and 1100 features. Of all the classifiers evaluated, CSVM, QSVM, and GSVM consistently achieve the highest overall accuracy in various feature subsets, underlining their efficacy in the diagnosis of cervical cancer. On the other hand, for the Mendeley LBC dataset, using 1100 features, the LSVM classifier achieves a peak accuracy of 99.9%, demonstrating continuous improvement. Similarly, QSVM and CSVM exhibit outstanding performance, with both models reaching their peak accuracies of 99.9% when using 1200 features. The MGSVM achieves a maximum accuracy of 99.5% with 500 features, with a lesser improvement observed with additional features. The MKNN classifier gradually gets better, reaching a maximum accuracy of 97.9% with 1200 features. The CKNN model demonstrates the most substantial enhancement compared to its initial performance, increasing from 94.8% with 100 features to 96.2% with 1200 features. Ultimately, LDA attains its peak performance of 99.0% when using the 1200 and 1300 features. Significantly, the majority of classifiers exhibit considerable enhancement up to 300–500 features, beyond which the improvements become more modest. The SVM-based classifiers, namely LSVM, QSVM, CSVM, and MGSVM, consistently demonstrate superior performance compared to the other classifiers across all feature counts. On the contrary, CKNN typically exhibits the lowest accuracy. The present study emphasises the significance of feature selection in optimising classifier performance, whereby the ideal number of features is subject to variation depending on the type of classifier.

The main conclusion drawn from Fig 8 is that the use of ANOVA feature selection in the aggregated deep features derived from the three CNNs (EfficientNetB0, MobileNet, and ResNet18) seems to enhance the accuracy of classification in comparison to utilising features from each CNN separately. This is evident from the increased accuracy scores observed in Fig. 8. This indicates that ANOVA feature selection efficiently eliminates irrelevant or redundant attributes within the dataset, enabling classifiers to concentrate on the most useful attributes for classification.

Other evaluation measures are computed to demonstrate the performance of Light-XAI, including sensitivity (Sens), specificity (Spec), F1 measure, precision (Prec), and MCC. Table 8 displays these calculated metric values. The table indicates that for the SIPaKMeD dataset, the highest average precision, the F1 score, the specificity, the MCC and the sensitivity are achieved using the CSVM classifier. These values are equal to 0.9727, 0.9727, 0.9928, 0.9638, and 0.9727, respectively. However, for the Mendeley LBC dataset, the maximum average precision, F1 score, specificity, MCC, and sensitivity are achieved using the QSVM classifier with values equal to 0.9982, 0.9982, 0.9984, 0.9936, and 0.9982.

**Table 8:** Performance metrics (variance) after the ANOVA feature selection process for the SIPaKMeD dataset and the LBC Mendely dataset

Classifier	Accuracy	Prec	F1-measure	Spec	MCC	Sens
SIPaKMeD dataset						
LSVM	0.9643 (9.2 × 10^− 7^)	0.9643 (9.2 × 10^− 7^)	0.9643 (9.2 × 10^− 7^)	0.9911 (5.5 × 10^− 8^)	0.9555 (1.5 × 10^− 6^)	0.9643 (9.2 × 10^− 7^)
QSVM	0.9727 (1 × 10^− 7^)	0.9727 (1 × 10^− 7^)	0.9727 (1 × 10^− 7^)	0.9928 (9.2 × 10^− 8^)	0.9638 (2.3 × 10^− 6^)	0.9727 (1 × 10^− 7^)
CSVM	0.9727 (1 × 10^− 6^)	0.9727 (1 × 10^− 6^)	0.9727 (1 × 10^− 6^)	0.9932 (5.7 × 10^− 8^)	0.9659 (1.6 × 10^− 6^)	0.9727 (1 × 10^− 6^)
GSVM	0.9699 (6.9 × 10^− 7^)	0.9699 (6.9 × 10^− 7^)	0.9699 (6.9 × 10^− 7^)	0.9925 (4.2 × 10^− 8^)	0.9624 (1 × 10^− 6^)	0.9699 (6.9 × 10^− 7^)
MKNN	0.9591 (6 × 10^− 6^)	0.9591 (6 × 10^− 6^)	0.9591 (6 × 10^− 6^)	0.9898 (3.5 × 10^− 7^)	0.9489 (9.7 × 10^− 6^)	0.9591 (6 × 10^− 6^)
CKNN	0.9660 (2.9 × 10^− 6^)	0.9660 (2.9 × 10^− 6^)	0.9660 (2.9 × 10^− 6^)	0.9915 (1.6 × 10^− 7^)	0.9574 (4.5 × 10^− 6^)	0.9660 (2.9 × 10^− 6^)
LDA	0.9572 (1.5 × 10^− 6^)	0.9572 (1.5 × 10^− 6^)	0.9572 (1.5 × 10^− 6^)	0.9878 (4.8 × 10^− 6^)	0.9470 (2.3 × 10^− 6^)	0.9572 (1.5 × 10^− 6^)
Mendeley LBC dataset						
LSVM	0.9966 (6.3 × 10^− 6^)	0.9966 (6.3 × 10^− 6^)	0.9966 (6.3 × 10^− 6^)	0.9978 (4.9 × 10^− 6^)	0.9915 (7.9 × 10^− 5^)	0.9966 (6.3 × 10^− 6^)
QSVM	0.9982 (7 × 10^− 7^)	0.9982 (7 × 10^− 7^)	0.9982 (7 × 10^− 7^)	0.9984 (5.6 × 10^− 6^)	0.9936 (8.9 × 10^− 5^)	0.9982 (7 × 10^− 7^)
CSVM	0.9968 (7 × 10^− 7^)	0.9968 (7 × 10^− 7^)	0.9968 (7 × 10^− 7^)	0.9969 (6.6 × 10^− 6^)	0.9879 (1.0 × 10^− 4^)	0.9968 (7 × 10^− 7^)
GSVM	0.9934 (1.3 × 10^− 6^)	0.9934 (1.3 × 10^− 6^)	0.9934 (1.3 × 10^− 6^)	0.9978 (1.1 × 10^− 7^)	0.9911 (1.6 × 10^− 6^)	0.9934 (1.3 × 10^− 6^)
MKNN	0.9792 (2.2 × 10^− 6^)	0.9792 (2.2 × 10^− 6^)	0.9792 (2.2 × 10^− 6^)	0.9932 (2.6 × 10^− 7^)	0.9725 (4.2 × 10^− 6^)	0.9792 (2.2 × 10^− 6^)
CKNN	0.9692 ((3.7 × 10^− 6^)	0.9692 ((3.7 × 10^− 6^)	0.9692 ((3.7 × 10^− 6^)	0.9875 (2.9 × 10^− 7^)	0.9501 (4.8 × 10^− 6^)	0.9692 ((3.7 × 10^− 6^)
LDA	0.9878 (6.7 × 10^− 6^)	0.9878 (6.7 × 10^− 6^)	0.9878 (6.7 × 10^− 6^)	0.9958 (4.8 × 10^− 7^)	0.9836 (7.9 × 10^− 6^)	0.9878 (6.7 × 10^− 6^)

In Table 8 it can be seen that all classifiers in the SIPaKMeD dataset exhibit excellent performance throughout each metric, with precision, F1 measure, and sensitivity consistently varying from 0.9582 to 0.9727. In particular, the SVM versions of the Mendeley LBC dataset exhibit even greater performance metrics. Remarkably, although SVM versions consistently achieve excellent performance on both datasets, the KNN and LDA classifiers exhibit greater variability between datasets, demonstrating significantly superior performance on the Mendeley LBC dataset.

**Fig. 9:**
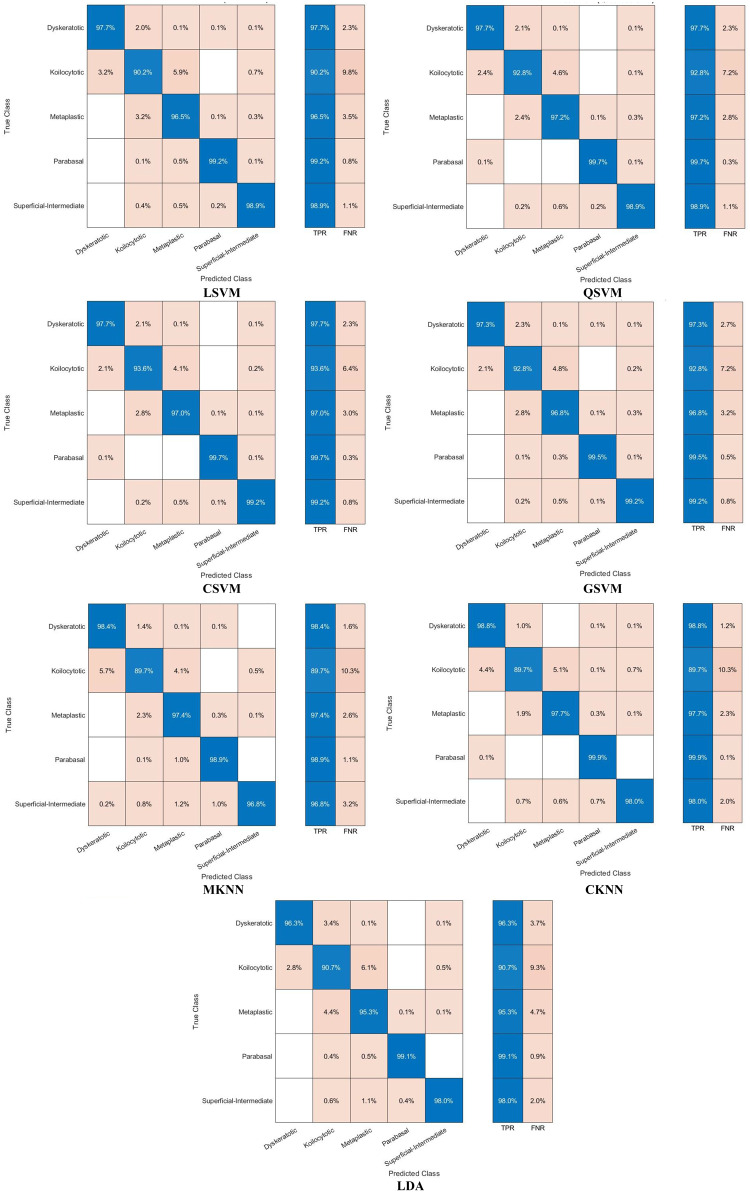
Confusion metrics for the seven classification models learnt with the picked attributes of ANOVA feature selection from the SIPaKMeD dataset

**Fig. 10:**
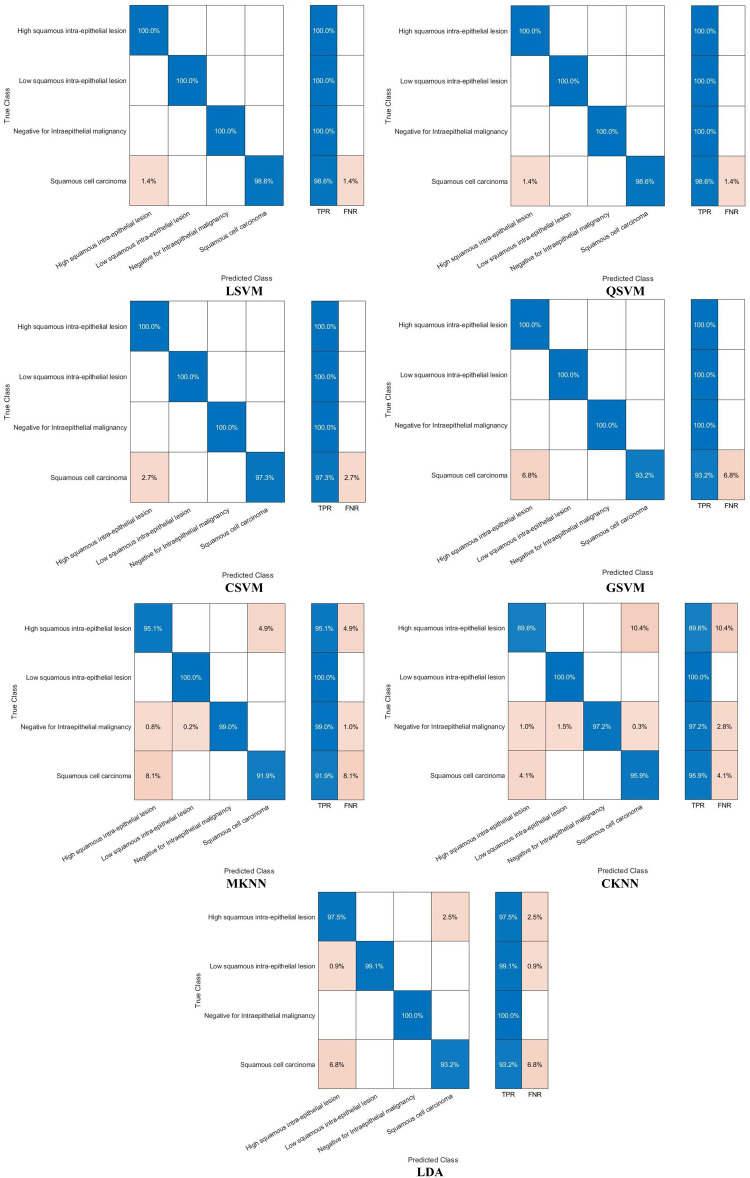
Confusion metrics for the seven machine learning classifiers learnt with selected features of ANOVA feature selection from the Mendeley LBC dataset

The confusion matrices for the seven machine learning classifiers, constructed with selected deep features using the ANOVA feature selection method, are displayed in Figs. 9 and 10. These matrices represent the classification tasks involved in differentiating between the subcategories of cervical cancer. Confusion matrices are only presented for the test data because they give an unbiased picture of how well the model works on samples it hasn’t seen before. This is very important for medical diagnosis applications.

Figure 9 demonstrates that each classifier of the Light-XAI model is capable of correctly recognising different categories of cervical cancer for the SIPaKMeD dataset. For instance, CSVM attains sensitivities for superficial intermediate, parabasal, koilocytes, dyskeratotic, and metaplastic cell classes equal to 99.2%, 99.7%, 93.6%, 99.7%, and 97.0%. In addition, the Light-XAI model classifiers effectively classify all categories of the Mendeley LBC dataset with a high level of sensitivity. For example, the LSVM and QSVM, which achieved the highest performance, have the following sensitivities for each class: intraepithelial malignancy, low-grade squamous intraepithelial lesion (LSIL), and high-grade squamous intraepithelial lesion are 100%, while for squamous cell carcinoma, the sensitivity is 98.6%.

The SVM models (LSVM, QSVM, CSVM, GSVM) routinely showed exceptional performance in all categories of cervical cancer, with high rates of correctly identifying positive cases and few instances of incorrectly classifying cases. CSVM and QSVM show almost flawless classification for most categories, with minimal confusion among closely associated abnormal cell types. LSVM and GSVM demonstrated outstanding performance, but with somewhat higher misclassifications compared to CSVM and QSVM. On the contrary, the MKNN classifier, although still attaining satisfactory overall performance, exhibited more significant misclassifications, especially among specific abnormal categories of cells. In contrast, CKNN exhibited the highest error rates compared to all classifiers, characterised by numerous incorrect classifications in different categories. The LDA classifier demonstrates strong performance, particularly in correctly identifying normal cells, but it showed some ambiguity in distinguishing between specific types of abnormal cells. The substantial disparity in performance between top-performing (SVM-based) and bottom-performing classifiers (CKNN) highlights the need for rigorous classifier selection in cervical cancer classification.

An examination of the confusion matrices of the Mendeley LBC dataset, as shown in Fig. 10, shows that SVM-based models (LSVM, QSVM, CSVM, GSVM) routinely perform better than other classifiers in all types of cervical cancer. Models exhibit robust true positive rates, low misclassifications, and almost flawless classification for most categories, with little ambiguity between similar abnormal cell types. Although the misclassifications of CSVM and GSVM are somewhat higher than those of LSVM and QSVM, they nevertheless maintain remarkably high accuracy. On the other hand, while the MKNN classifier overall achieves satisfactory results overall, exhibits more significant misclassifications compared to the SVM models, especially when discriminating between specific atypical cell types. The CKNN model exhibits the highest error rates, characterised by regular misclassifications in several categories. The LDA classifier displays some ambiguity when distinguishing between particular types of abnormal cells. This comprehensive examination of the confusion matrices offers critical insights into the capabilities and limitations of each classifier in differentiating various types of cervical cells, thus enhancing the overall effectiveness of the Light-XAI model in classifying cervical cancer.

**Fig. 11:**
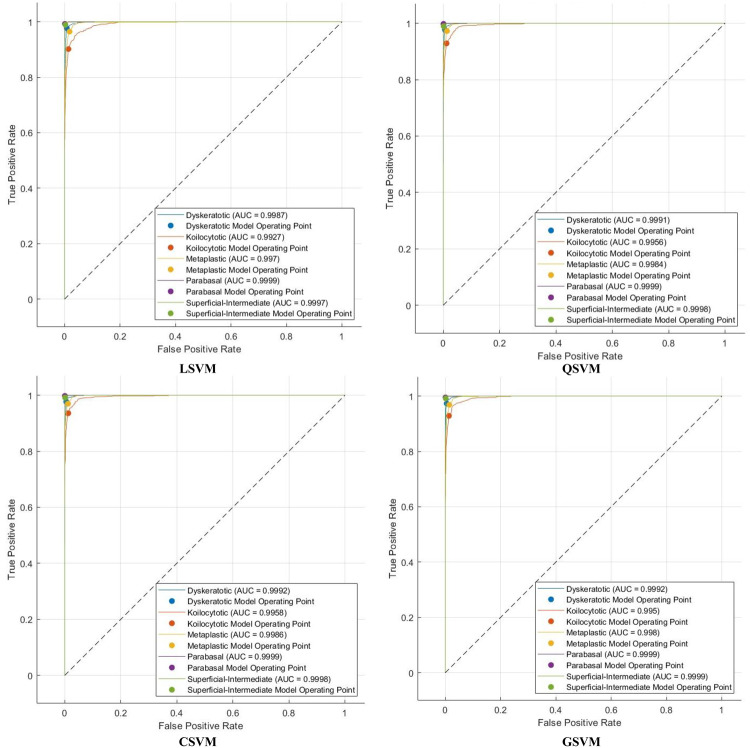

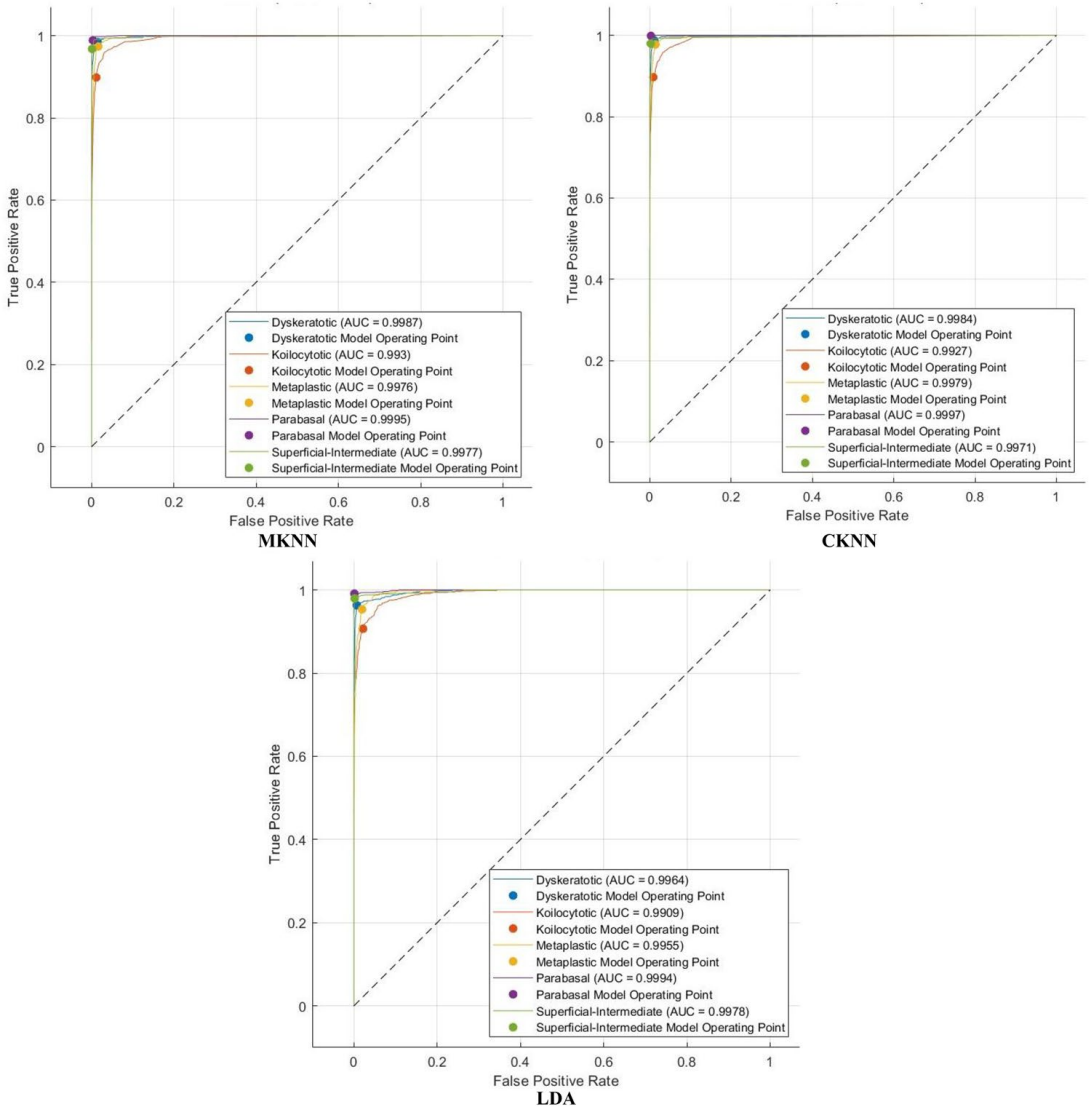
ROC curves for the seven machine learning classifiers learnt with selected features of ANOVA feature selection of the SIPaKMeD dataset

**Fig. 12:**
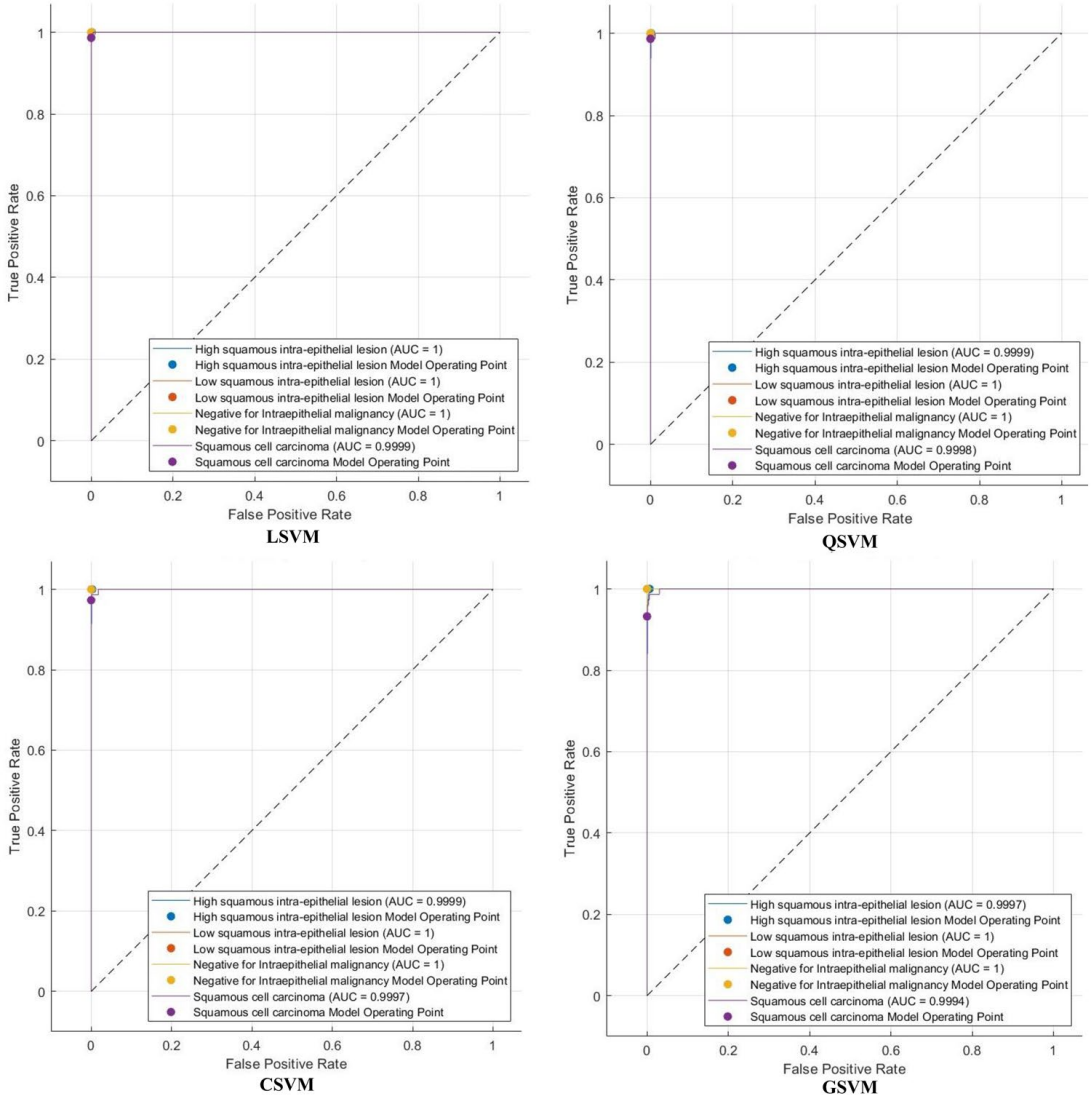

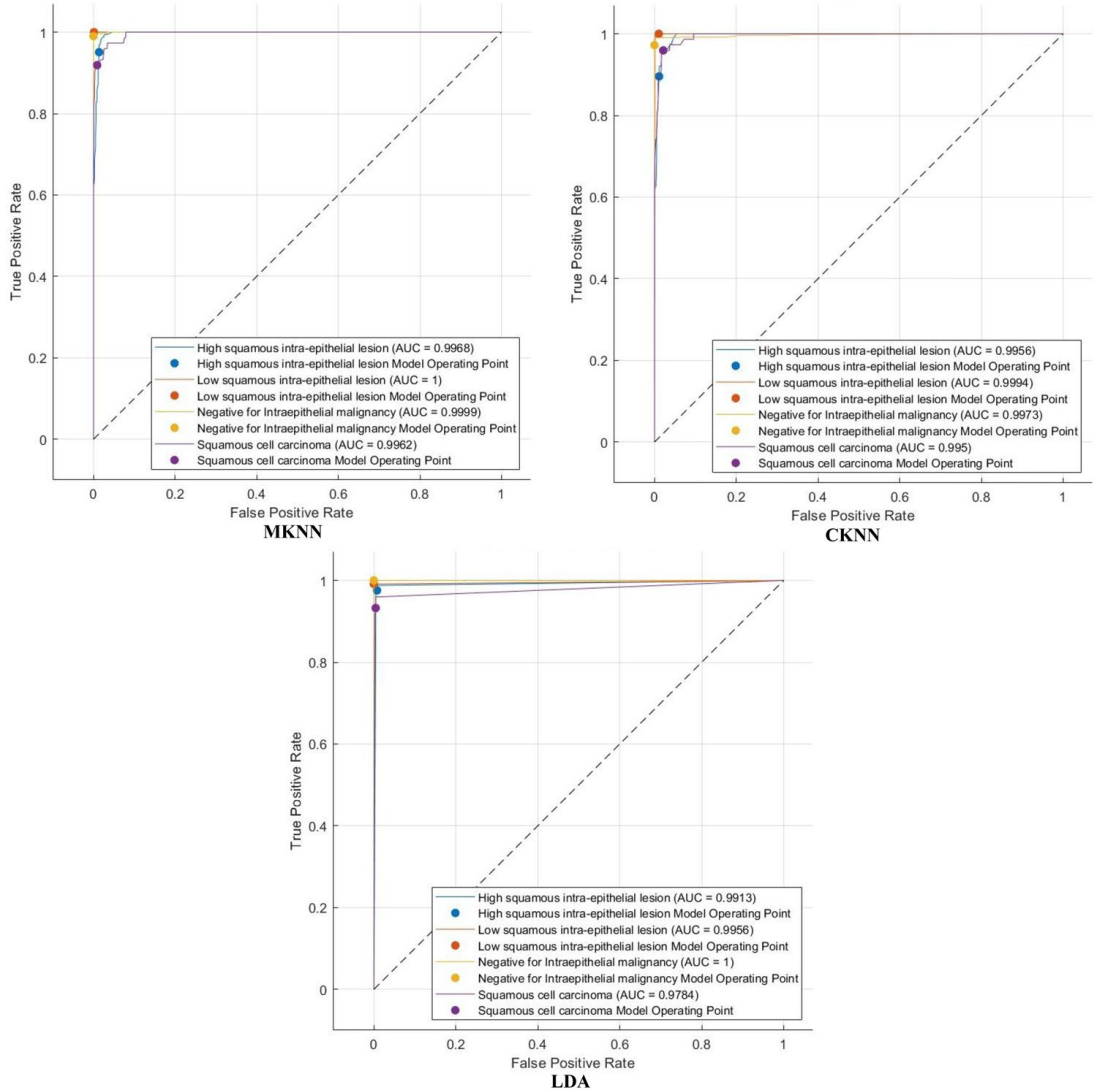
ROC curves for the seven machine learning classifiers learnt with selected features of ANOVA feature selection of the Mendeley LBC dataset

For the SIPaKMeD and Mendeley LBC datasets, the ROC curves that were achieved by applying machine learning classifiers following the feature selection stage are shown in Figs. 11 and 12, respectively. These types of curves are produced by the ANOVA feature selection technique, which takes into account all the variables that are selected. According to the figures, the score of the y-axis reflects the sensitivity, whilst the x-axis indicates the magnitude of the one-specificity measurement. All such ROC curves have been created while assuming that an individual type of cervical cancer has been determined to be the positive classification indicator. This assumption has been made for each of these graphs. With regard to the ROC curves, the Light-XAI model yields results that are consistent across classes of both datasets. All classifiers accomplish an area under the curves (AUCs) that is greater than 0.99 for both datasets, as shown by the ROC curves. These results illustrate the remarkable capacity to distinguish between different classifications of cervical cancer. The high AUC values suggest that Light-XAI is capable of accurately differentiating between positive and negative cases, while also achieving a consistently low false positive rate. Moreover, the ROC curves demonstrate that the performance of Light-XAI remains rather uniform across all categories considered in each dataset. This indicates that the model can achieve high accuracy in classifying various forms of cervical cancer. Based on this, it can be deduced that Light-XAI is equipped with the ability to accurately differentiate among the different categories that are contained within both datasets.

A one-way analysis of variance test was performed on the outcomes acquired from the repetitive five-fold cross-validation procedure to evaluate the statistical significance of the observed variations in classifier accuracies. ANOVA was chosen over nonparametric tests, given that the statistical analysis was performed on mean performance values derived from repeated stratified five-fold cross-validation runs, resulting in nearly symmetric distributions with low variance. Under these circumstances, ANOVA is frequently utilized in the literature to compare classifier performance across different setups and parameters, as it offers a clear and transparent evaluation of intergroup differences. The statistical analysis in this study aimed to ascertain the importance of observed performance disparities, rather than to mimic random distributional behavior.

The null hypothesis (H₀) predicts that there is no statistically significant variation in the average accuracy between the classification methods. First, the one-way analysis of variance test was used to analyse the accuracy outcomes of classifiers that were trained using the SIPaKMeD dataset. The results, shown in Table 9, indicate that the *p*-values achieved were dramatically below the predetermined alpha level of 0.05. The observed trend suggests a statistically significant disparity in the accuracy of the classifiers that were trained using this particular dataset. Furthermore, the identical test was applied to the accuracy outcomes of the classifiers that were trained using the Mendeley LBC dataset. Table 10 demonstrates that the *p*-values achieved for this dataset were less than 0.05, indicating a statistically significant disparity in the accuracies of classifiers trained on the Mendeley LBC dataset.

**Table 9:** One-way analysis of variance test results for the SIPaKMeD dataset

Source of variation	SS	df	MS	F	P value
Columns	0.00102	6	0.00017	80.33	<0.0001
Error	0.00006	28	~0		
Total	0.00108	34

**Table 10:** One-way analysis of variance test results for the Mendeley LBC dataset

Source of variation	SS	df	MS	F	P value
Columns	0.00498	6	0.00083	269.23	<0.0001
Error	0.00009	28	~0		
Total	0.00507	34			

## Discussions

This research introduced a CADx model named ‘Light-XAI’, which aims to classify Pap smear images and provide explanations for its results. The focal aims of Light-XAI are to incorporate a self-attention module into lightweight CNNs and investigate the impact of attention training on the performance of CNNs. Additionally, create a streamlined CNN-based diagnostic system that is both simple in design and highly accurate. In addition, generate comprehensive attributes from both deep layers of a deep learning model using a Pap-smear image. Moreover, the process involves combining deep features extracted from multiple layers of a deep neural network using a feature reduction technique. Furthermore, produce a compact set of meaningful deep attributes from the ensembles of CNNs that can effectively differentiate between various categories of Pap smear images. Ultimately, explore and interpret the discovery of the suggested system.

**Fig. 13:**
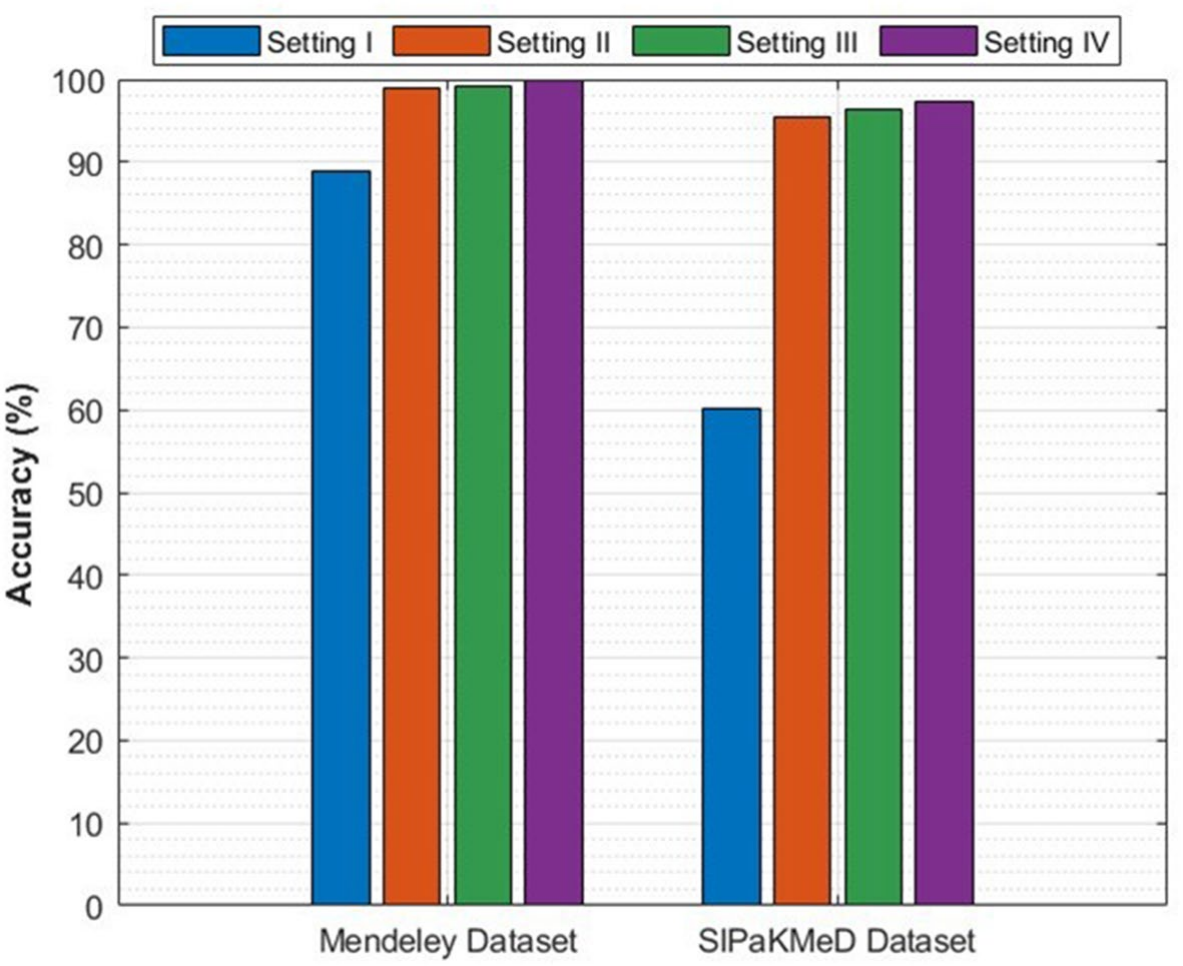
The highest accuracy attained for every classification setting for the SIPaKMeD dataset and the Mendeley LBC dataset

Of the four settings of Light-XAI, Setting IV attains the greatest accuracy rate of 97.41%. This indicates that employing feature selection on the merged features from two distinct layers throughout the three self-attention CNNs yields the most distinguishing information for precise classification of the SIPaKMeD dataset. It is worth mentioning that the feature size in Setting IV (1100) is lower than in Setting II (1280) and larger than in Setting III (512). However, this increase in feature dimension results in a notable enhancement in classification accuracy. This underscores the efficacy of feature selection in discerning the most relevant attributes for the classification operation. Figure 13 demonstrates a comparison of the greatest accuracy achieved in each setting for both datasets.

In the SIPaKMeD dataset, Setting I, which involves end-to-end classification, reaches a minimal accuracy of 60.21%. These findings indicate that the use of a CNN as a classifier may not yield optimal results for this particular dataset. Settings II, III, and IV exhibit markedly superior accuracy compared to Setting I. These findings demonstrate that the use of deep attributes acquired out of the intermediary layers of the CNN (Settings II and III) and the implementation of the feature selection approach (Setting IV) significantly enhances the accuracy of the SIPaKMeD dataset.

Like the SIPaKMeD dataset, Setting I obtains the least accuracy (88.93%) for the Mendeley LBC dataset. This further implies that end-to-end classification may not be the most optimal approach for these specific Settings II, III, and IV demonstrate significantly superior accuracy in comparison to Setting I, highlighting the importance of employing deep features or feature selection for classification. Out of the aforementioned settings, setting IV again attains the greatest degree of accuracy, reaching 99.90%. These findings indicate that selecting features from the aggregated attributes of two layers in three CNNs yields the most informative data for accurately classifying the Mendeley LBC dataset. Like the SIPAKMED dataset, the advantage of choosing the most relevant features outweighs the decrease in the length of the feature in Setting IV (1100) in comparison to Setting II (1280), and the increase in the number of features compared to Setting III (512) leads to a significant improvement in the accuracy of classification.

The accuracy disparity noted among Setting I (end-to-end CNN classification) and Setting II (CNN feature extraction followed by traditional classifiers) is scientifically anticipated and substantiated by existing medical image analysis literature, especially in cytology-based CADx tasks characterized by small numbers of samples and significant intra-class variability.In Setting I, lightweight CNNs are trained in an end-to-end manner, necessitating the network to concurrently acquire low-level representations of features and refine a softmax-based decision boundary. In these circumstances, the learning process is hindered by the restricted representational capabilities of compact structures and the challenges associated with simultaneously optimizing feature extraction and classification of nuanced cytological patterns. The models converge consistently but reach a plateau at comparatively low accuracy, signifying capacity-constrained learning (underfitting) instead of overfitting or experimental instability.

Conversely, Setting II intentionally separates representational learning from classification, an approach that has consistently demonstrated benefits in medical imaging when datasets are of moderate dimension and class boundaries are non-linear. In this context, CNNs function solely as static deep feature extractors, generating high-dimensional and conceptually rich representations. Conventional classifiers, especially margin-based models like cubic SVMs, are employed on these features, demonstrating proficiency in establishing resilient decision boundaries within intricate feature spaces without necessitating comprehensive gradient optimization.

This change in perspective clarifies the extent of the reported performance improvement. The enhancement arises not from the mere substitution of a softmax layer, but from alleviating the optimization constraints placed on compact CNNs during end-to-end training, thereby enabling classifiers more adept at handling limited data scenarios to leverage the discriminative characteristics of the extracted deep features. This phenomenon is extensively documented in cervical cytology and broader biomedical CADx research, where CNN+SVM frameworks frequently surpass fully end-to-end CNNs despite their seemingly straightforward nature.

Furthermore, in the suggested Light-XAI framework, the deep features utilized in Settings II and IV are not simply raw CNN outputs; rather, they are refined through multi-layer extraction, wavelet-based fusion, and feature selection, which augment inter-class separability and diminish intra-class variance prior to classification. Thus, traditional classifiers function within a more organized and discriminative feature space, rendering the observed enhancement in accuracy both credible and methodologically warranted.

This study developed the Light-XAI framework to be a computer-assisted screening tool. The proposed framework does not intend to replace a doctor’s clinical diagnosis or judgment with a computer-generated analysis. By accurately differentiating cytological cell types along with Grad-CAM based visual explanations that highlight both the nucleus and the cytoplasm, the Light-XAI Framework could help to reduce the volume of slides that cytopathologists must review when they screen for potential malignancies; this will also allow for better prioritisation of suspicious slides and ultimately, more consistent diagnoses among cytopathologists who utilise the Light-XAI framework to perform their job. The lightweight nature of the proposed framework enables it to be utilised in clinical settings that may not have access to high-level computer hardware and software (e.g., low resource settings)

To evaluate the proposed Light-XAI framework’s effectiveness, the study performed the experiment using two distinct public datasets. The SIPaKMeD dataset included isolated images of single cells, and there was a high level of consistency with respect to the environment and conditions used to collect these images compared to the more complex and heterogeneous Mendeley LBC dataset that contained multiple cell images from many different labs with varying colour, staining techniques, background structure, and images of both normal and abnormal cells; this shows that the Light-XAI framework performs consistently and robustly across both public datasets that contain the majority of the variability that is present in clinical cytology.

### Explainability analysis

**Fig. 14:**
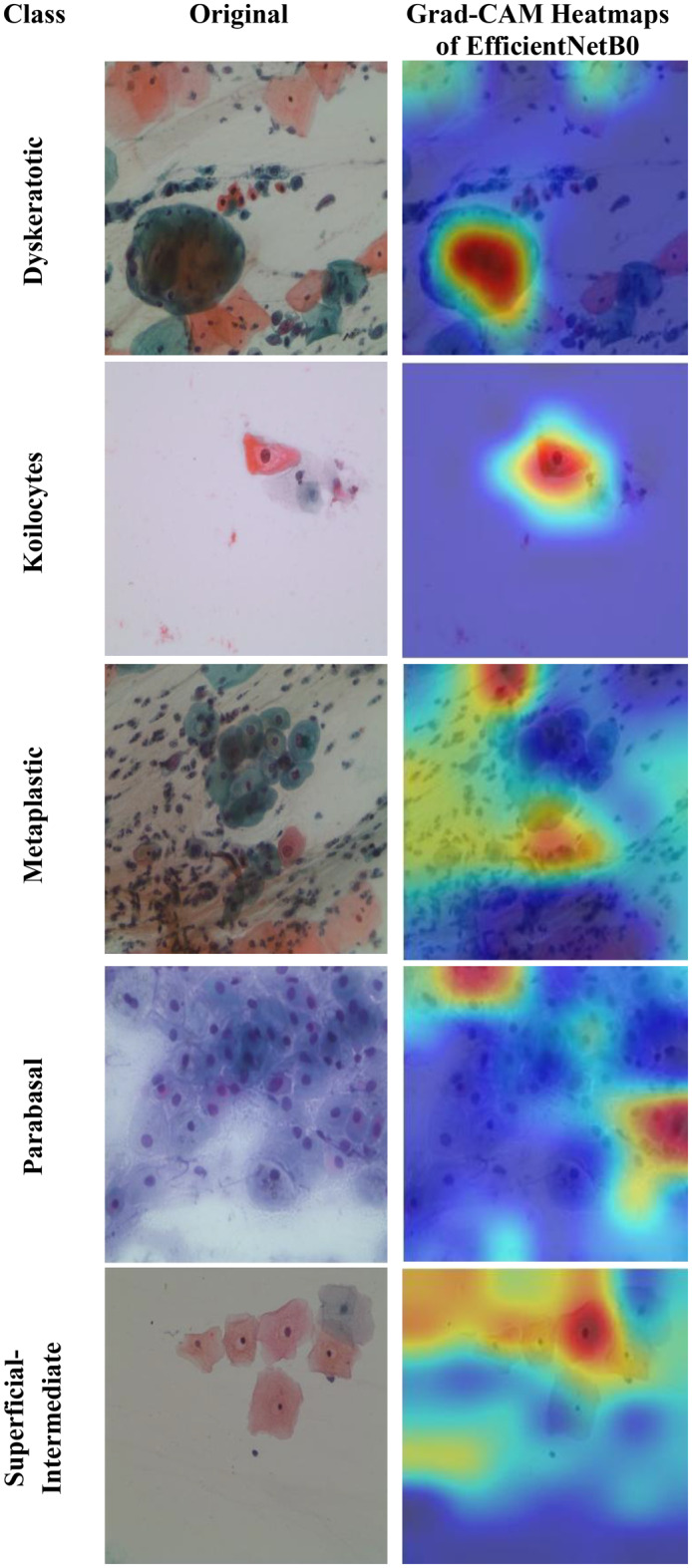
Instances of Grad-CAM analysis on the Pap smear images of the SIPaKMeD dataset for EffficientNetB0

**Fig. 15:**
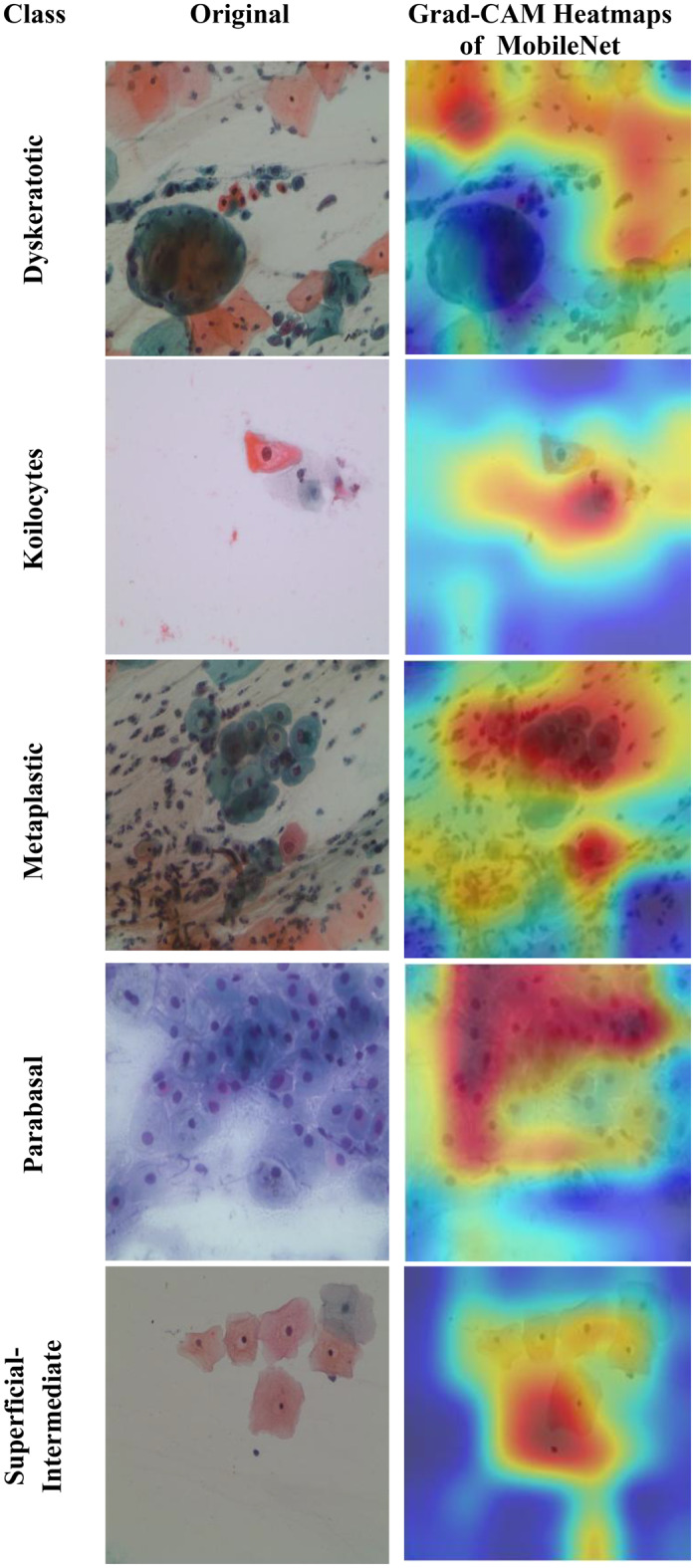
Instances of Grad-CAM analysis on the Pap smear images of the SIPaKMeD dataset for MobileNet

**Fig. 16:**
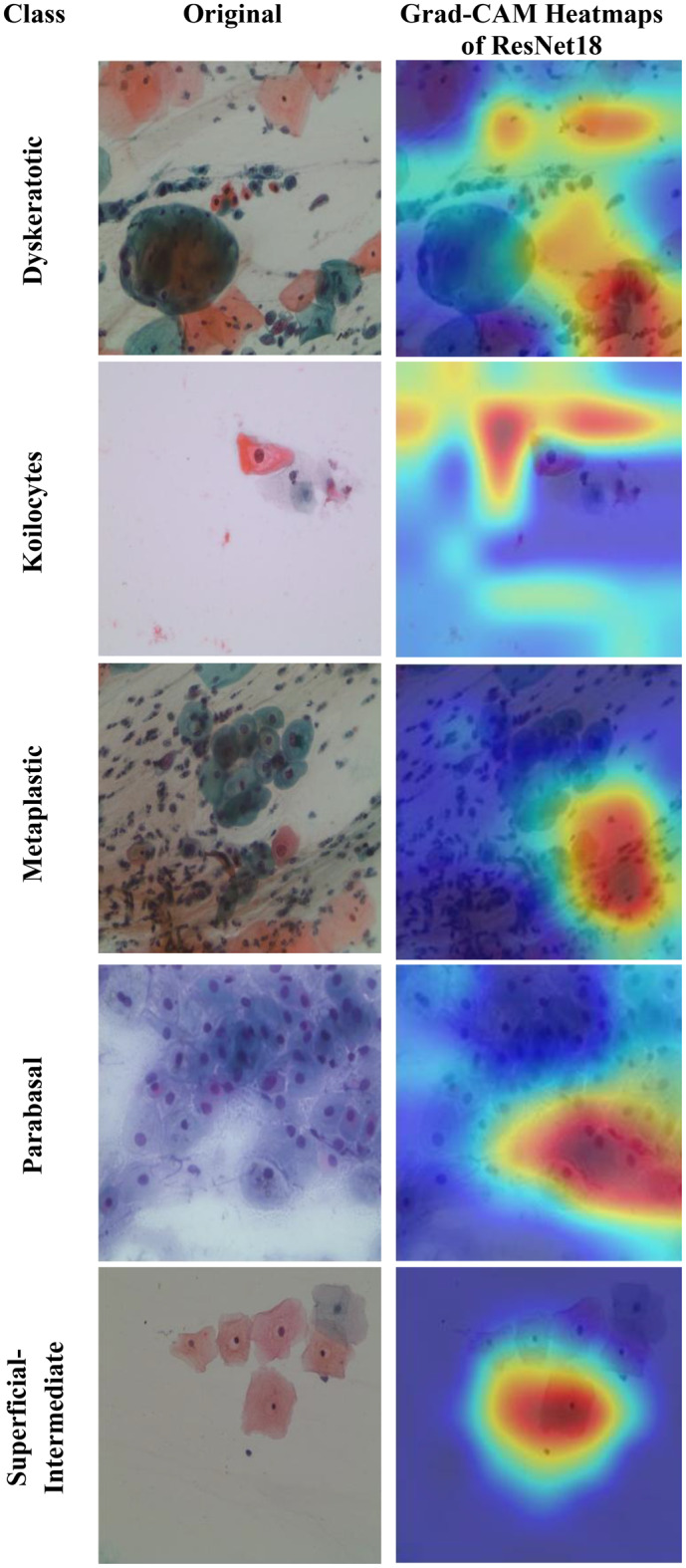
Instances of Grad-CAM analysis on the Pap smear images of the SIPaKMeD dataset for ResNet18

**Fig. 17:**
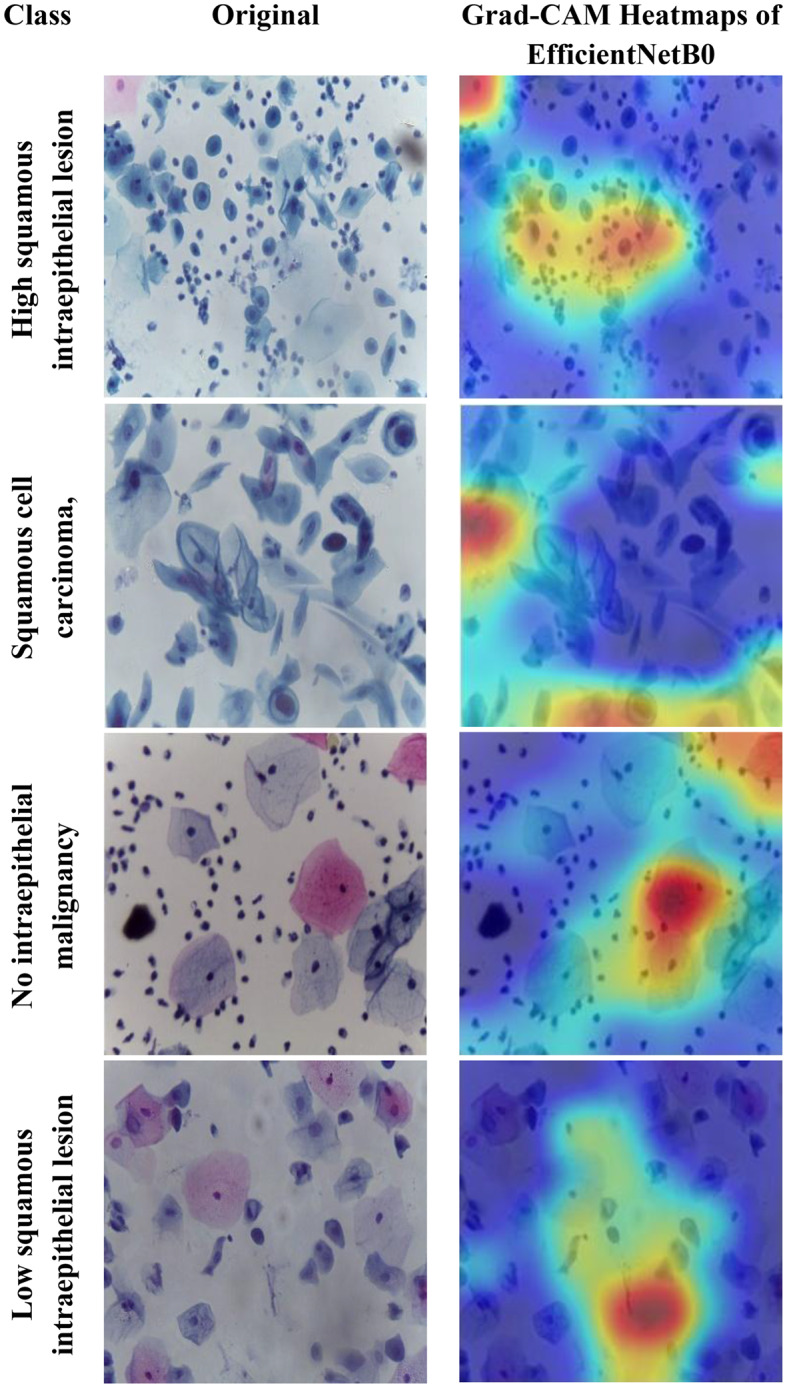
Instances of Grad-CAM analysis on the Pap smear images of the Mendeley LBC dataset for EfficientNetB0

**Fig. 18:**
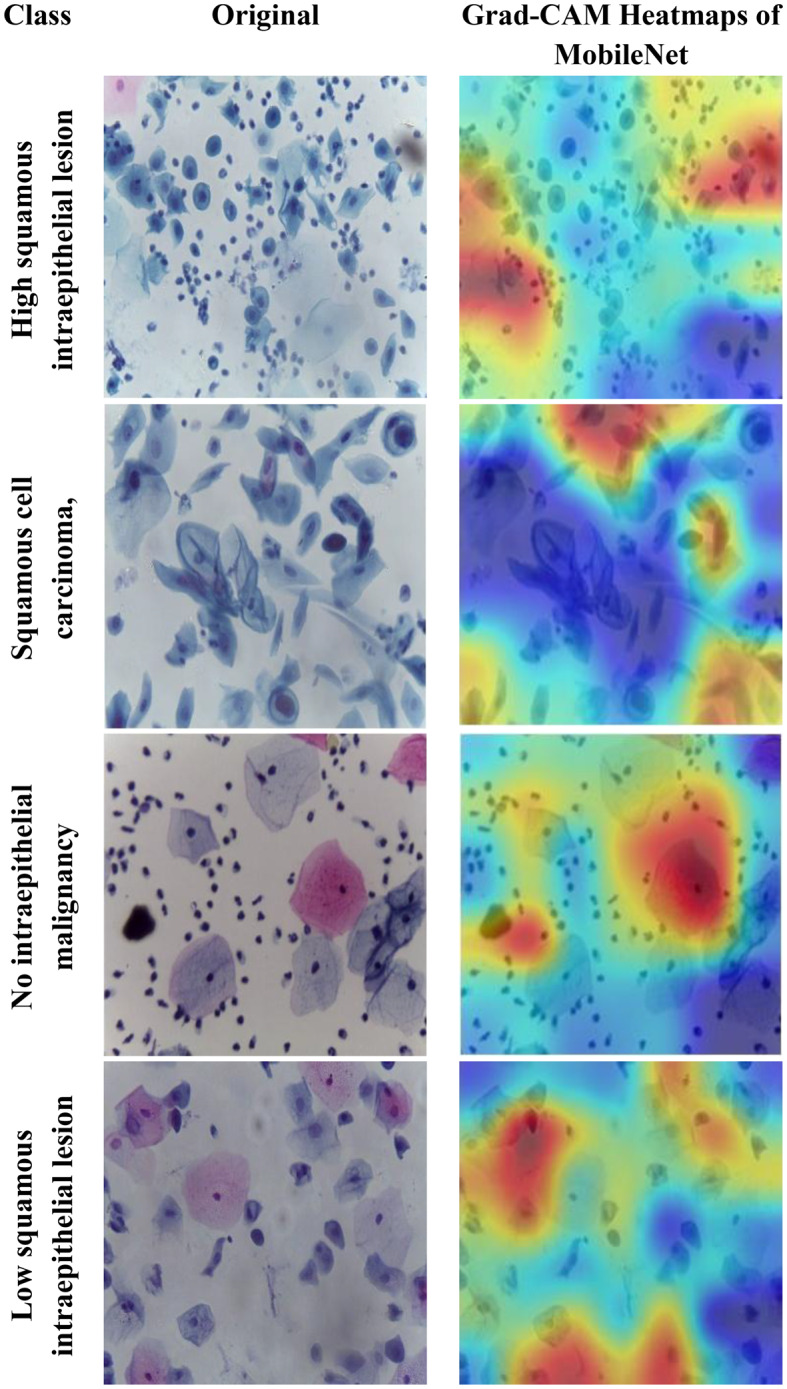
Instances of Grad-CAM analysis on the Pap smear images of the Mendeley LBC dataset for MobileNet

**Fig. 19:**
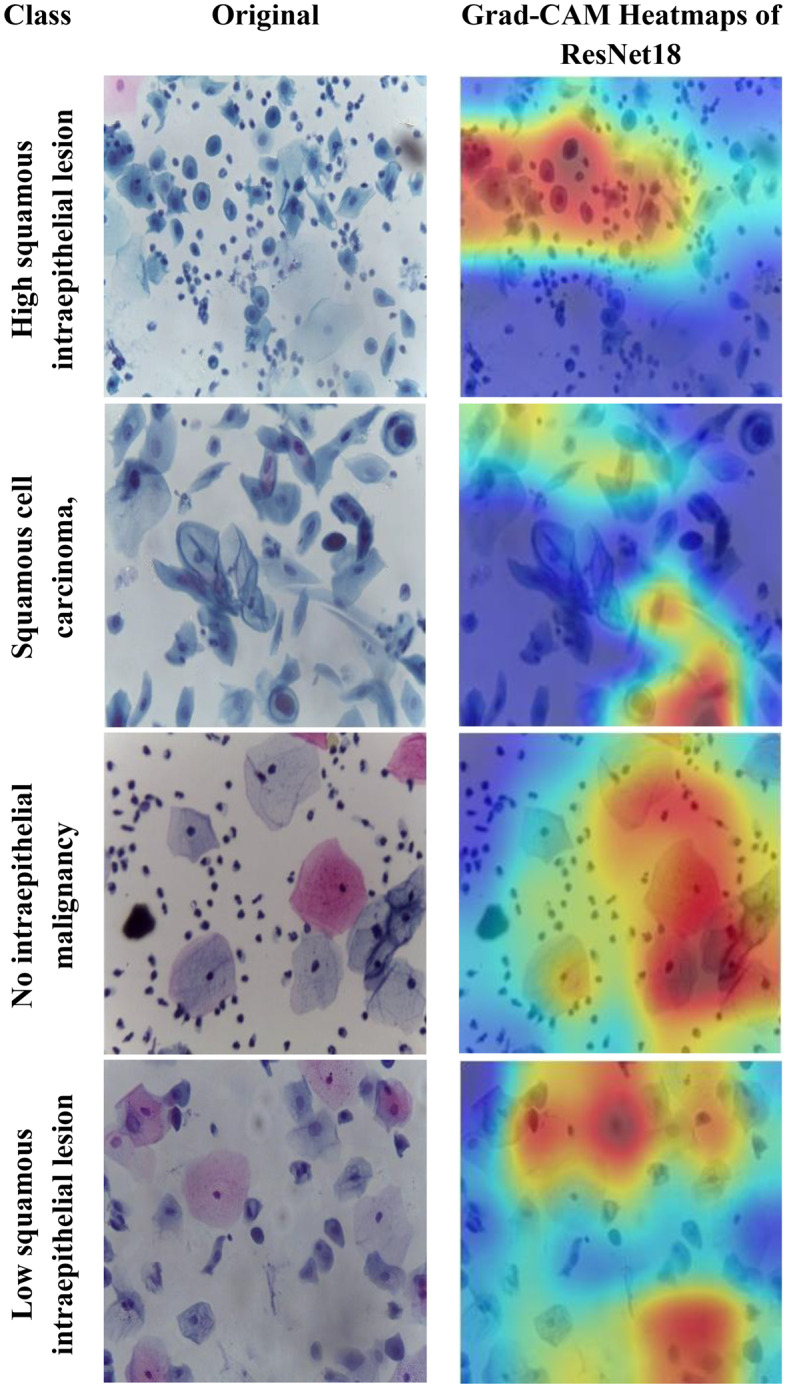
Instances of Grad-CAM analysis on the Pap smear images of the Mendeley LBC dataset for ResNet18

This research uses Grad-CAM in order to gain an understanding of the decision-making process of the CNN model that is used to categorise cervical cancer categories based on Pap smear photos. CNN uses this visualisation approach to bring attention to specific regions within the Pap smear image that are taken into account during the classification process. By analysing the Grad-CAM results, we are able to acquire beneficial insights into the reasoning that the model uses to classify a picture as belonging to a specific kind of cervical cancer. Understanding the model’s emphasis on particular cell attributes or morphological patterns that are essential for accurate classification can be of particular assistance. Although the interpretability that Grad-CAM provides cannot only increase confidence in the predictions made by the model, it also has the capacity to contribute to the discovery of unfamiliar image features that are pertinent to the diagnosis of cervical cancer. The impacted area of the photo is represented by a heat map that is generated by the Grad-CAM. A selection of the findings can be seen in Figs. 14, Figs. 15, 16 for the SIPaKMeD dataset and Figs. 17, Figs. 18, 19 for the Mendeley LBC dataset. Throughout this investigation, the jet colour pattern is used. Within this pattern, the lighter blue shades are characterised by smaller values, which indicates that no variables have been acquired for a specific category. Green and yellow shades, on the other hand, exhibit intermediary values, which indicate that significantly fewer features have been captured. Finally, the red and darker red shades contain higher values, indicating that the attributes found in the area are representative of the specific class [90].

The present study aims to perform a thorough analysis of the areas that are often highlighted by the proposed CADx for categorising various forms of cervical cancer in both the SIPaKMeD and Mendeley LBC datasets. The feature extraction procedure is the central component of any deep learning-driven classification task. The higher the level of information provided by a feature, the greater the possibility of achieving precise classification. One highly effective tool now used by academics to visualise the feature maps generated by CNN algorithms is Grad-CAM. The Grad-CAM algorithm identifies the areas of the pap smear photo that have the greatest impact on the classification decision of the model by computing the gradient of the class-specific score in relation to convolutional feature maps [91]. In this context, warmer hues (such as red and yellow) denote regions of greater significance, whereas cooler hues (such as blue) signify areas of lesser significance. This visualisation facilitates the identification of different cellular characteristics, such as irregular cell structure, nuclear atypia, and glandular structures, which serve as indicators of various types of cervical cancer [92]. The analysis of the Grad-CAM heatmaps produced for the SIPaKMeD and Mendeley LBC datasets provides valuable insights into the model’s capacity to effectively capture these crucial visual cues and achieve precise classifications. Grad-CAM is used to qualitatively evaluate explainability through clinically relevant activation of the nucleus and cytoplasm. This approach aligns with existing methodologies for cytological computer-aided diagnosis (CADx) research, in which there is no reliable way to provide pixel-level saliency ground truth.

The analysis demonstrates that the Grad-CAM heatmaps generally highlight distinct cellular characteristics linked to each type of cancer, which helps the CNN make a decision. Based on an analysis of Figs. 14–17, it can be concluded that the three self-attention CNN models take into account distinct regions to determine the image class. For example, in Fig. 14, the EfficientNetB0 model mostly takes into account the nucleus, whereas the MobileNet (Fig. 15) and ResNet18 (Fig. 16) models have distinct edges from the cervical cell regions for the Dyskeratotic and Koilocyte classes. In contrast, the ResNet18 (Fig. 1^16^) model takes into account the middle cytoplasmic area for prediction in the superficial intermediate, parabasal, and Metaplastic classes. Figures 17 to 19 illustrate that the EfficientNetB0 and ResNet18 models prioritise the central region between the round nucleus for the High squamous intraepithelial lesion, while the MobileNet model concentrates on the left and right sections of the cytoplasm. Regarding the categories Low squamous intraepithelial lesion and no intraepithelial malignancy, the EfficientNetB0 model focuses on the main area of the cytoplasm. On the contrary, the ResNet18 and MobileNet models exhibit modest to moderate activation in all regions, but a robust activation concentrated in the nucleus region. On the contrary, the three CNNs specifically target the lower regions of the class of cytoplasm for the Squamous cell carcinomas.

It can be noted from Figs. 14–19 that EfficientNetB0, MobileNet, and ResNet-18 have different activation patterns. This is because each backbone utilizes unique structural concepts—such as compound scaling in EfficientNetB0, depthwise separable convolutions in MobileNet, and residual learning in ResNet-18—resulting in variations in receptive fields and feature prioritization. Consequently, Grad-CAM emphasizes distinct yet complementary areas while maintaining attention on clinically pertinent structures. The variation in activation patterns justifies the impetus for multi-backbone feature fusion in the proposed Light-XAI framework.

It is evident from the aforementioned observations that each of these models employs a distinct feature extraction procedure. Therefore, the use of the ensemble CNN enhances the potential of the proposed Light-XAI to acquire additional details, as illustrated in the figures. The results achieved by the Light-XAI approach demonstrate this. These results highlight the efficacy of Light-XAI in detecting crucial visual indicators that help in the categorisation of cervical cancer. Through its emphasis on these particular areas, our model shows its capacity to recognise the nuanced morphological alterations that are distinctive of various types of cancer.

Early-stage cervical cancer examinations are intentionally extremely sensitive; nonetheless, the prevalence of excessive false-positive results continues to pose a significant clinical concern. Elevated false-positive rates may result in superfluous follow-up interventions (e.g., repeat cytology, colposcopy, or biopsy), augmented workload for cytopathologists, heightened patient concern, and elevated healthcare expenditures. Consequently, CADx systems designed for practical application must not only attain high accuracy but also exhibit strong specificity and precision to reduce false positives.

The Light-XAI framework demonstrates significantly elevated specificity, precision, and F1-scores on both the SIPaKMeD and Mendeley LBC datasets, as evidenced by the confusion matrices and numerical findings. These metrics demonstrate that the model effectively identifies negative cases and minimizes the incorrect identification of benign or low-risk cases as malignant. This feature is particularly advantageous in an operational screening workflow, as it facilitates triaging and prioritization instead of indiscriminately reporting all suspicious instances. Furthermore, Light-XAI functions as a computer-assisted second-opinion system, supporting rather than substituting cytopathologists. By identifying high-confidence anomalous instances while ensuring a minimal false-positive rate, the approach enables doctors to concentrate on genuinely high-risk samples, thus enhancing effectiveness and diagnostic consistency. The model’s lightweight design enhances its integration into standard screening processes without demanding significant computing or infrastructural resources.

The use of Grad-CAM-based explainability enhances medical trust and safety by facilitating visual examination of the model’s focus. The activation maps indicate that the model primarily concentrates on clinically significant areas, including nuclear borders, chromatin configurations, and cytoplasmic structures, rather than background artifacts. This interpretability enables doctors to confirm that decisions are based on recognized cytological criteria, hence diminishing the likelihood of erroneous predictions that could lead to false positives.

From a translational standpoint, the integration of low false-positive rates, interpretability, and computing efficiency renders Light-XAI a viable support tool for extensive cervical cancer screening, especially in environments with restricted expert access. Although prospective clinical validation is necessary before implementation, the existing findings indicate that Light-XAI may diminish superfluous follow-up procedures and enhance diagnostic workflows, hence improving patient care and resource efficiency.

### Comparative assessment

Table 11 presents a comparison between the Light-XAI CADx model and several previous CADx models that were evaluated using similar datasets. The results clearly establish the superior performance of Light-XAI in effectively categorising cervical cancer. The Light-XAI results are superior to those of current CADx models in terms of accuracy (ACC), F1 measure, sensitivity, and specificity in the two datasets (SIPaKMed and Mendeley LBC). For example, Light-XAI attains an accuracy of 97.41% in the SIPaKMed dataset, exceeding the accuracies obtained by previous research except for the study [67], which used a ViT, which requires a significant amount of computational power and time, and studies [52, 58], which require multiple preprocessing and processing steps, that adds to the cost of computation. Additionally, this substantial enhancement continues over additional metrics too, with Light-XAI displaying an F1 measure of 0.9828, sensitivity of 0.9780, and specificity of 0.9880, all of which are superior to the values that were earlier presented. Light-XAI yields an accuracy of 99.90% on the Mendeley LBC dataset, surpassing the previous best accuracy of existing CADs. Similar patterns can be noticed using the Mendeley LBC dataset. In terms of F1 measure (0.9952), sensitivity (0.9980), and specificity (0.9918), this strength situation is conclusively maintained.

**Table 11:** Comparing the results of the Light-XAI CADx model with earlier CADx methods that depended on Pap smear datasets

Study	Methodology	Database	Features Amount	Accuracy	F1-measure	Sensitivity	Precision
[53]	Bilinear CNNs + Random projection	Herlev	N/A	0.9530	0.9382	-	-
[56]	Dividing the photos into multiple subphotos. DarkNet-19 +NCA+SVM	SIPaKMeD Mendeley LBC	1000	0.9671 0.9927	0.972 0.9829	0.9673 0.9763	0.9670 0.9897
[55]	ResNet + Simple Logistic classifier	Herlev	N/A	0.9202	0.9210	0.9200	0.9210
[50]	Texture Features + GA+ MLP	Herlev	N/A	0.9630	-	-	-
[60]	Statistical + Texture Features + Cubic SVM	Mendeley LBC	68	0.9510	-	-	-
[93]	Morphological Features + Shape and Compactness Features + Orientation and Spatial Distribution Features + LBP + Histogram-based gradient boosting	Private	N/A	0.9592	-	0.9592	0.9640
[58]	Median Filter + Histogram Equalisation Cervical Net + ShuffleNet + CCA	SIPaKMeD	544	0.991	-	-	-
[68]	Xception + ViT	Combined (CRIC +SIPaKMeD)	2240	0.9172	0.9170	0.9160	0.9180
[66]	CNN + ViT	Herlev	N/A	0.9860	0.9840	0.9810	0.9750
[59]	Inception + ResNet-50 +DenseNet-121 + VGG-16 + GWO+ PCA	SIPaKMeD Mendeley LBC Herlev	796	0.9787 0.9947 0.9832	0.9889 0.9920 0.9812	0.9912 0.9927 0.9765	0.9856 0.9914 0.9866
[67]	MaxCerViT	SIPaKMeD Mendeley LBC	N/A	0.9902 0.9948	0.9902 0.9952	0.9904 0.9980	0.9903 0.9926
[64]	MobileNet+Inception + InceptionResNet	SIPaKMeD Mendeley LBC Herlev	N/A	0.9647 0.9968 0.9856	0.9645 0.9987 0.9858	0.9653 0.9934 0.9853	0.9651 0.9934 0.9865
[62]	DenseNet121 + MobileNet + LSTM	SIPaKMeD	2048	0.9580	0.9795	0.9580	1
[65]	Inception+Xception+DenseNet-169 + Fuzzy rank	SIPaKMeD Mendeley LBC	N/A	0.9543 0.9923	0.9536 0.9918	0.9538 0.9923	0.9534 0.9913
[62]	ViT+MobileNet	SIPaKMeD	N/A	0.9760	0.9858	0.9765	0.9954
[52]	Segmentation via the SURF and the Otsu threshold Compact VGG	SIPaKMeD Herlev	N/A	0.9780 0.9481	0.9828 0.9646	0.9780 0.9552	0.9877 0.9742
[57]	ResNet-18+GoogleNet + O-bHSA + SVM	SIPaKMeD Mendeley LBC	748 706	0.9704 0.9958	0.9700 1	0.9700 1	0.9700 1
[63]	Xception + VGG16 + Inception + Product Voting	SIPaKMeD Mendeley LBC	N/A	0.9721 0.9979	0.9714 0.9974	0.9715 0.9992	0.9716 0.9956
[23]	Customised XAI CNN	Mendeley LBC	N/A	0.9750	0.9750	0.9750	0.9750
[51]	DenseNet201	Herlev	N/A	0.8702	0.6165	0.6341	0.6226
[75]	improved MobileNetv3 + stacked extreme learning machine	Herlev	N/A	0.9896	0.9571	0.9627	0.9571
[94]	Squeeze-and-Excitation Inceptionv4 + ResNet-152 + DenseNet169 + Fuzzy Rank-Based Fusion	SIPaKMeD Mendeley LBC	N/A	0.9718 0.9922	0.9716 0.9919	0.9714 0.9917	0.9719 0.9922
[95]	MobileNet, DenseNet, EfficientNet, Xception, RegNet, and ResNet-50 + PCA	SIPaKMeD	676	0.9700	0.9700	0.9700	0.9700
[96]	ConvLSTM layers and Squeeze-and-Excitation + Differential Evolution	SIPaKMeD Mendeley LBC	N/A	0.9568 0.9896	0.9574 0.9850	0.9567 0.9895	0.9582 0.9897
Light-XAI	EfficientNetB0+MobileNet+ResNet18+DWT+ANOVA + SVM	SIPaKMeD Mendeley LBC	1100 1100	0.9727 (1 × 10^− 6^) 0.9982 (7 × 10^−7^	0.9727 (1 × 10^− 7^) 0.9982 (7 × 10^− 7^)	0.9727 (1 × 10^− 7^) 0.9982 (7 × 10^− 7^)	0.9727 (1 × 10^− 7^) 0.9982 (7 × 10^− 7^)

N/A signifies that a feature selection method has not been utilized in this study, while (-) shows metrics that were not published in the original studies; direct statistical comparisons across previous research are impractical due to the lack of raw experimental data.

The results presented in Table 11 collectively indicate that Light-XAI represents noteworthy progress in the diagnosis of cervical cancer. Due to its outstanding results across a wide range of metrics, it is a promising tool for improving accuracy in medical imaging evaluation operations, which could eventually contribute to improved patient outcomes. The superiority of Light-XAI over current CADx systems for cervical cancer classification lies in its ability to overcome several limitations of traditional cervical cancer classification models. In contrast to prior research that depends on individual CNN architectures or extracting features from a single layer, Light-XAI uses a multi-CNN ensemble supported by self-attention mechanisms. This novel methodology allows the system to extract both local and global characteristics from pap smear photos, resulting in enhanced classification accuracy. Moreover, the integration of feature selection and fusion techniques in Light-XAI sets it apart from conventional CADx systems. By aggregating features from several layers and choosing the most informative ones, Light-XAI efficiently decreases the number of dimensions in the feature space, reducing the likelihood of overfitting and improving the efficiency of classification. The incorporation of explainability using the Grad-CAM approach is another significant contribution of this work. In contrast to other opaque deep learning models, Light-XAI offers clinicians a significant understanding of its decision-making process, boosting confidence and promoting the integration of AI-assisted diagnosis in clinical settings.

Conventional (non-deep learning) cervical cancer CAD methodologies have been clearly incorporated into Table 11 to facilitate a more thorough comparison. These encompass techniques utilizing handcrafted texture features in conjunction with genetic algorithms and multilayer perceptrons [50], statistical and texture descriptors paired with cubic SVM classifiers on the Mendeley LBC dataset [60], and morphological, shape, compactness, and spatial distribution features classified with gradient boosting [93]. Table 11 highlights that different non DL methodologies yield accuracies between 95 and 96%, demonstrating the efficiency of carefully engineered features for cervical cytology analysis.

Nevertheless, these conventional methods rely heavily on manual feature engineering, which can restrict resilience when faced with differences in staining, imaging settings, and cellular morphology typically observed in real-world clinical operations. Furthermore, handcrafted features may fail to accurately represent sophisticated and high-dimensional cytological structures linked to early-stage abnormalities, potentially leading to elevated false-positive rates when models are utilized outside the original data distribution for which they were developed.

Conversely, the proposed Light-XAI system, as shown in Table 11, utilizes automatically acquired deep representations using lightweight CNN architectures while ensuring computational effectiveness and interpretability. The constantly elevated precision and specificity demonstrated by Light-XAI on public datasets indicate a greater potential for minimizing false-positive predictions relative to conventional feature-based methods. This is especially critical in cervical cancer screening, as an abundance of false positives might result in unwarranted follow-up procedures, heightened burden for cellular pathologists, and greater patient concern.

### Computational efficiency comparison

Table 12 provides a detailed comparison of the computational efficiency of the proposed Light-XAI framework when compared to various CNN-based, hybrid CNN-Transformer, and Transformer CADx models for Pap smear image analysis. While previous comparisons have mainly focused on diagnostic accuracy, the table also lists several key metrics that are vital to the successful implementation of a CADx solution into clinical practice, including architectural depth, number of parameters, and computational cost. While the absolute values for training and inference timeframe can differ greatly based on hardware and software optimization, the FLOPs, number of deep layers, and total model size parameters provide a common point of comparison that can be replicated across many platforms, thus enhancing the credibility and impact of the results presented in this study. Thus, Table 12 shows these metrics for comparison.

**Table 12:** Computational efficiency comparison of the Light-XAI CADx model with earlier CADx methods that depended on Pap smear datasets

Study	Model Category	Backbone/Architecture	Params (Approx.)	FLOPs	# Deep Layers (Approx.)
[57]	CNN	ResNet-18+GoogleNet + O-bHSA + SVM	≈18.5 M	1.81 G +1.50 G = 3.31 G	≈18 + 22 ≈ 40
[63]	CNN Ensemble	Xception + VGG16 + Inception	22.9 M + 138.4 M + 23.8 M ≈ 185 M	4.8 G + 15.5 G + 5.7 G ≈26 G	≈36 + 16 + 48 ≈ 100
[59]	Multi-CNN + Optimization	Inception + ResNet-50 + DenseNet-121 + VGG-16	8 M + 25.6 M + 23.8 M + 138.4 M	2.9 G + 3.9 G + 5.7 G + 15.5 G ≈ 28 G	≈48 + 50 + 121 + 16 ≈ 235
[64]	Multi-CNN Fusion	MobileNet + Inception + Inception-ResNet	3.4 M+23.8 M + 55.8 M ≈ 83 M	0.3 G + 5.7 G + 13.2 G ≈ 19 G	53 + 48 + 164 ≈ 265
[65]	CNN Ensemble + Fuzzy Rank	Inception + Xception + DenseNet-169	23.8 M + 22.9 M + 14.3 M ≈ 61 M	5.7 G + 4.8 G + 3.4 G ≈ 14 G	≈48 + 36 + 169 ≈ 253
[67]	Transformer	MaxCerViT	16.4 M	3.39 G	>100 layers (stages + blocks)
[68]	Hybrid CNN–Transformer	Xception + tiny DeiT	22.9 M + 5.7 M ≈ 29 M	4.8 G + 1.25 G ≈ 6 G	≈36 + 12 blocks ≈48
[62]	CNN + RNN	DenseNet-121 + MobileNet + LSTM	7.9 M + 3.4 M ≈ 11.5 M	2.9 G + 0.3 G ≈ 3 G	≈121 + 53 ≈ 174
[62]	CNN + Transformer	MobileNet + ViT	3.4 M +86.6 M ≈ 100 M	17.6 G + 0.3 G ≈ 18 G	53 + 12 Transformer blocks
[94]	Heavy CNN Ensemble	SE-Inceptionv4 + ResNet-152 + DenseNet-169	43 M + 60.2 M + 14.2 M ≈ 117 M	12.5 G +11.3 G +3.4 G ≈ 27 G	≈164 + 152 + 169 ≈ 485
[97]	Transformer	ViT-B/16	86.6 M	≈17.6 G	12 Transformer blocks
[31]	CNN Ensemble	ResNet-18 + DarkNet-19 + MobileNet	11.7 M + 20.8 M + 3.4 M ≈ 36 M	1.81 G + 2.6 G + 0.3 G ≈ 4.71 G	18 + 19 + 53 ≈ 90
Light-XAI (Proposed)	Lightweight CNN + XAI	EfficientNet-B0 + MobileNet + ResNet-18 + DWT + ANOVA	≈5.3 + 3.5 + ~11.8 ≈ 20.6 M	≈0.39 G + ~0.31 G + 1.82 G ≈ ~3.0 G	18 + 53 + 18 ≈ 89

The results from the table indicate that the majority of successful CADx systems are designed as either deep networks (e.g., CNNs, Transformers) or as ensembles of multiple models (e.g., Multi-CNN). As shown in [50–52], and [81], Multi-CNN ensemble configurations consist of 80–200 million parameters, operate at GFLOPs of 19–28, and contain > 200 layers deep, indicating that these systems are highly architecturally and computationally complex. Although the classification performance produced by these models is impressive, the extremely high inference costs will severely limit the practicality of these systems in resource-constrained screening settings and for formal clinical use (i.e., real-time).

While Transformer-based approaches and hybrid CNN-Transformers models provide robust means of modeling global dependencies, they do so at the expense of additional complexity, memory use, and deep layers. For example, ViT-B/16 [97] has around 86.6 million parameters, 17.6 GFLOPS, and 12 transformer blocks; MaxCerViT [67] has over 16 million parameters, 3.39 GFLOPS, and more than 100 hierarchical transformer layers, though its design is more compact than ViT-B/16‘s. Additionally, hybrid CNN-Transformer models such as Xception + Tiny DeiT [68] use approximately 29 million parameters, 6 GFLOPS, and nearly 50 deep layers; these models require significantly more hardware and memory resources when compared to lightweight, CNN-based architectures. Additionally, as noted in [47], a hybrid CNN–Transformer model that combines MobileNet and ViT has demonstrated how much additional computation is required when using ViT as components in a system. Using a lightweight CNN (MobileNet, ~3.4 million params) still adds a large portion to the total size of the framework (around 100 million params) due to the Giant ViT component added to the framework, with 18 GFLOPS (total Computational Cost) added to it also. This framework has an architecture made up of 53 Convolutional Layers and 12 Transformer Blocks. Due to the added effect of the ViT component, creating extra impact on the overall complexity.

Conversely, Light-XAI has a significantly more balanced trade-off between accuracy and efficiency compared with other methods. By combining lightweight CNN backbones (EfficientNet-B0, MobileNet, and ResNet-18) with feature-level fusion techniques, DWT-based integration, and statistical selection of features, the total parameters of Light-XAI are limited to approximately 20.9 M, the total computation cost to about 3.0 GFLOPS, and the total architectural depth to only 64 layers. Thus, relative to most ensemble and transformer methods, Light-XAI has considerably less complexity while achieving state-of-the-art diagnostic capability.

Table 12 also indicates that improvements in marginal accuracy have been demonstrated by heavier models like those in Attallah et al. [31], with a corresponding increase in the amount of computing power required to train, test, and operate these models. Therefore, in many clinical screening situations, particularly in lower-resource environments, it is not a good trade-off. Consequently, Light-XAI has chosen to focus on improving the efficiency, robustness, and explainability of its models, which are consistent with the practical requirements for larger-scale cervical cancer screening systems.

### Light-XAI limitations and future directions

Although this study presents a thorough assessment across two benchmark datasets with various staining procedures and imaging workflows, proving resilience in a range of cytological settings, a number of limitations should be noted in order to appropriately contextualize the contributions and specify precise avenues for further research. For example, the classification procedure may be significantly affected by unbalanced data, as it may lack sufficient training data for certain subcategories, possibly decreasing its efficacy. To enhance the reliability and applicability of the model in clinical settings with various individual demographics, the collaborative efforts of pathologists and expert validation are indispensable. The effectiveness of Light-XAI is greatly dependent on the amount and diversity of the data used to learn its fundamental CNN models. Learning biases can lead to inaccurate categorisations, particularly for lesions with atypical characteristics or for cervical cancer subtypes that are not well represented in the dataset.

The SIPaKMeD collection comprises isolated single-cell images, with every image depicting a distinct cytological case rather than a segment derived from a collective whole-slide image. Consequently, slide- or patient-level leaking is naturally prevented for this collection. On the other hand, the Mendeley LBC dataset comprises distinct cytology pictures that may feature several cells; nevertheless, it is freely accessible without slide detail or patient identification, hence inhibiting the implementation of explicit patient-wise separation.

Light-XAI is a tool that helps in decision-making and should not be relied upon as the main source of diagnosis. The expertise and proficiency of pathologists in using Pap smear imaging and medical evaluation are essential to guarantee reliable diagnosis and interpretation of the system’s results. One possible drawback of Light-XAI is that its findings may not effectively reflect the demographic characteristics of individuals from different racial backgrounds due to the lack of diversity in training data. The publicly accessible datasets utilized in this research (SIPaKMeD and Mendeley LBC), although commonly employed benchmarks, may not adequately reflect the demographic, ethnic, and geographic diversity that exists in actual cervical cancer detection operations. Age distribution, ethnicity, socioeconomic status, and regional screening techniques can impact cytological characteristics and disease incidence, thereby impacting model efficacy when applied to varied populations.

This study elucidates that the suggested Light-XAI framework was assessed by stratified cross-validation and other performance indicators, which aids in alleviating class-level bias; yet, this does not comprehensively resolve wider demographic fairness issues. The lack of demographic metadata in the utilized public datasets restricts the capacity to perform subgroup-specific fairness evaluations, a constraint prevalent in numerous existing cervical cytology investigations and now explicitly recognized. Furthermore, the study highlights that the ethical use of CADx systems necessitates meticulous external validation throughout demographically varied populations, in addition to prospective clinical investigations with experienced cytopathologists. Future research will concentrate on broadening the assessment of Light-XAI to multi-center datasets with demographic annotations, integrating fairness-aware evaluation processes, and evaluating possible biases among population subgroups, in accordance with evolving ethical AI standards.

The study used Grad-CAM XAI for visualising and clarifying the areas that the model prioritises during classification, providing interpretability. Future research will examine further XAI techniques, like Local Interpretable Model-Agnostic Explanations (LIME) or Shapley additive explanations (SHAP), in recognition of the significance of broader interpretability approaches in order to gain a deeper understanding of the model’s decision-making process. Furthermore, while Grad-CAM can provide clinically interpretable, intuitive visual explanations, this study is focused on qualitative explainability analysis due to the lack of expert annotations (pixel-level/region-level) from public datasets used. Quantitative methods for evaluating explainability, such as the accuracy of localization with respect to expert-annotated regions of interest and saliency consistency metrics across models and folds, would provide an important area for future research. Furthermore, this study recognizes that with the incorporation of expert-annotated regions, explainability can be evaluated rigorously and objectively. Future work will include the evaluation of clinically annotated regions and the development of quantitative XAI evaluation frameworks, once available, particularly for clinical Pap smear datasets or large-scale datasets.

In this current research study, generalizability was assessed using two independent publicly available Pap smear datasets: The SIPaKMeD dataset and the Mendeley LBC dataset. The two datasets differ from each other in both the staining protocol used to create images and the imaging environment and pipeline by which those images were created. Therefore, consistent performance across both datasets suggests a high degree of generalizability. Furthermore, the Herlev dataset was not employed in this study as it differs from SIPaKMeD and Mendeley LBC in terms of class taxonomy and diagnostic framework. The Herlev dataset contains seven classes that classify cells by degree of abnormality and malignancy progression and have been manually selected as single-cell images, while SIPaKMeD and Mendeley LBC are both liquid-based cytology images, each having its own separate class definitions and preparation protocols. The SIPaKMeD dataset employs a morphology-driven classification scheme with five categories that classify cells based on their morphological attributes instead of the degree of severity of disease. Likewise, the Mendeley LBC dataset utilizes liquid-based cytology with distinct class descriptions and procedures for preparation that diverge from traditional Pap smear classifications. The straightforward incorporation of Herlev necessitates class remapping, label aggregation, or the removal of specific categories, thereby leading to ambiguity and diminishing the credibility of compared findings. Consequently, numerous recent cervical cancer CADx investigations limit their evaluation to datasets with congruent class definitions, as implemented in this study.

Furthermore, the incorporation of noisy clinical samples or private hospital datasets, although significantly beneficial, generally necessitates ethical permissions, regulated datasets-sharing protocols, as well as accessibility to annotated clinical data, which were outside the focus of the present research. This limitation is prevalent in the cervical cytology literature, as preliminary validation is frequently performed on selected public datasets prior to clinical use. Subsequent efforts will concentrate on broadening validation to encompass additional public datasets.

One more limitation is that the study did not incorporate resampling-based imbalance handling techniques (including SMOTE and class-weighted losses) into the analysis of the Mendeley LBC dataset. The aim of this was to prevent the introduction of synthetic samples or distorted class prior probabilities, which may distort subtle patterns within Pap smear cytology images, and ultimately impede clinical realism. This concern has recently been reported in other studies within the realm of biomedical imaging. This effect of imbalance is now well-documented, and future investigations will take a thorough look at how to address this issue by using techniques such as SMOTE, class-weighted losses, and cost-sensitive learning on larger and more diverse datasets. The addition of this clarification to the text provides greater transparency regarding the methods used and will make the methods used more complete than they were previously.

Future research will also investigate the use of deeper deep learning models, such as ViT and Capsule Networks. In addition, future research will explore additional ensemble and fusion methodologies. In addition, other methods for reducing features, such as stacked autoencoders and variational autoencoders, will be examined. Furthermore, future research will explore the use of sampling approaches or generative adversarial networks (GANs) to address issues related to class imbalance. Furthermore, it is imperative to gather additional data from a larger number of patients in different scan centers in order to enhance generalisability and optimise the performance. While Light-XAI’s main emphasis is on categorising cervical cancer types, there is the possibility of adapting its basic framework to identify other prevalent cancers or diseases. An effective approach is to boost the training dataset with additional data that are specifically relevant to those illnesses and subsequently fine-tune the model to enhance its accuracy. In future research, Light-XAI will be evaluated in clinical environments by qualified pathologists.

The absence of a consistent pattern in the accuracy results among the seven classifiers with various configurations can be attributed to multiple contributing factors. To begin with, different hyperparameters and data features can affect how well machine learning models function. The interaction of such elements can result in intricate patterns of nonmonotonic behaviour. Furthermore, the exact construction and training methods used in each classification model can lead to differences in precision. Ultimately, the nature of the dataset itself can play a role. The complexity, noise level, and class distribution of the data can introduce additional elements that impact the performance of the classification models. In order to get a more comprehensive understanding of the fundamental causes for the observed nonmonotonic trends, future studies should further investigate these aspects.

The Light-XAI system that has been suggested has considerable promise for practical implementation in the field of cervical cancer screening. The remarkable precision and comprehensibility of this tool can increase the diagnostic capabilities of pathologists, resulting in more precise and prompt diagnoses. However, a number of factors need to be taken into account for implementation to be successful. First, it is necessary to assess the applicability of the model to different patient populations and specific clinical environments. Furthermore, effective incorporation of current clinical processes and electronic health records platforms would be crucial to guarantee smooth implementation. Furthermore, it is imperative to address privacy and ethical issues associated with the use of patient data. Lastly, continuous tracking and assessment of the model’s performance in real-world applications would be required to detect possible constraints and guide future enhancements. The importance of expert opinion in validating the medical significance of the findings of Light-XAI is highly acknowledged. In future investigations, the author will explore collaborating with medical professionals to enhance the clinical validity of our proposed Light-XAI system.

Although partial ablation impacts are analyzed in controlled experimental environments and through DWT-level assessments, upcoming studies will investigate more detailed ablation and runtime benchmarking to better delineate the contributions of specific modules.

## Conclusions

This paper introduced Light-XAI, a comprehensive CADx system that classifies cervical cancer by systematically integrating self-attention mechanisms with lightweight CNN, multi-layered feature fusion, and XAI techniques. Light-XAI efficiently captured both local and global characteristics from pap smear photographs, surpassing conventional CNNs that depend on local characteristics. In the experiments conducted on two benchmark datasets, the results showed that Light-XAI exhibited exceptional classification performance, especially when used in conjunction with feature selection and fusion methods. Therefore, the importance of feature extraction, fusion, and selection was emphasised to achieve the best possible classification outcomes. In addition, the transparency of Light-XAI, facilitated by the Grad-CAM approach, provides clinicians with a significant understanding of the decision-making procedure implemented by the model. Enhanced trust and ultimately improved patient outcomes were facilitated by the produced heatmaps, which graphically depict the most significant areas of the photo that impact classification. Light-XAI is a notable breakthrough in the domain of cervical cancer identification, providing a superb blend of high precision and comprehensibility. Potential future research could investigate the incorporation of Light-XAI into practical clinical environments to evaluate its influence on patient care. Furthermore, exploring the applicability of Light-XAI to other categories of cervical anomalies would be a vital avenue for future study. Furthermore, in future studies, we will focus on validating ethical considerations by assessing the proposed framework using cohorts that exhibit a variety of demographic characteristics, as well as through the use of fairness-aware evaluation protocols, to verify that the framework provides equitable results when applied in an actual cervical cancer screening program.

## Electronic supplementary material

Below is the link to the electronic supplementary material.

Supplementary material 1

Supplementary material 2

Supplementary material 3

Supplementary material 4

## Data Availability

Link to SipakMed dataset: https://www.kaggle.com/datasets/prahladmehandiratta/cervical-cancer-largest-dataset-sipakmed Link to Mendeley LBC dataset: https://data.mendeley.com/datasets/zddtpgzv63/4. The framework was developed using MATLAB 2023a. The code is available at the following GitHub link: https://github.com/omneya83/cervical-cancer-classification.git.
