# Supplementary material for: Light-XAI: a CADx for explainable cervical cancer detection via attention-based lightweight convolutional neural networks and layer-wise feature fusion
Source: BioData Min. 2026 Apr 10;19:26. doi: 10.1186/s13040-026-00540-6 (PMC13085275; doi:10.1186/s13040-026-00540-6)
Supplement: Supplementary file 1 — Supplementary material 1 [file 13040_2026_540_MOESM1_ESM.docx]

| 1. **Effiicient-B0** | 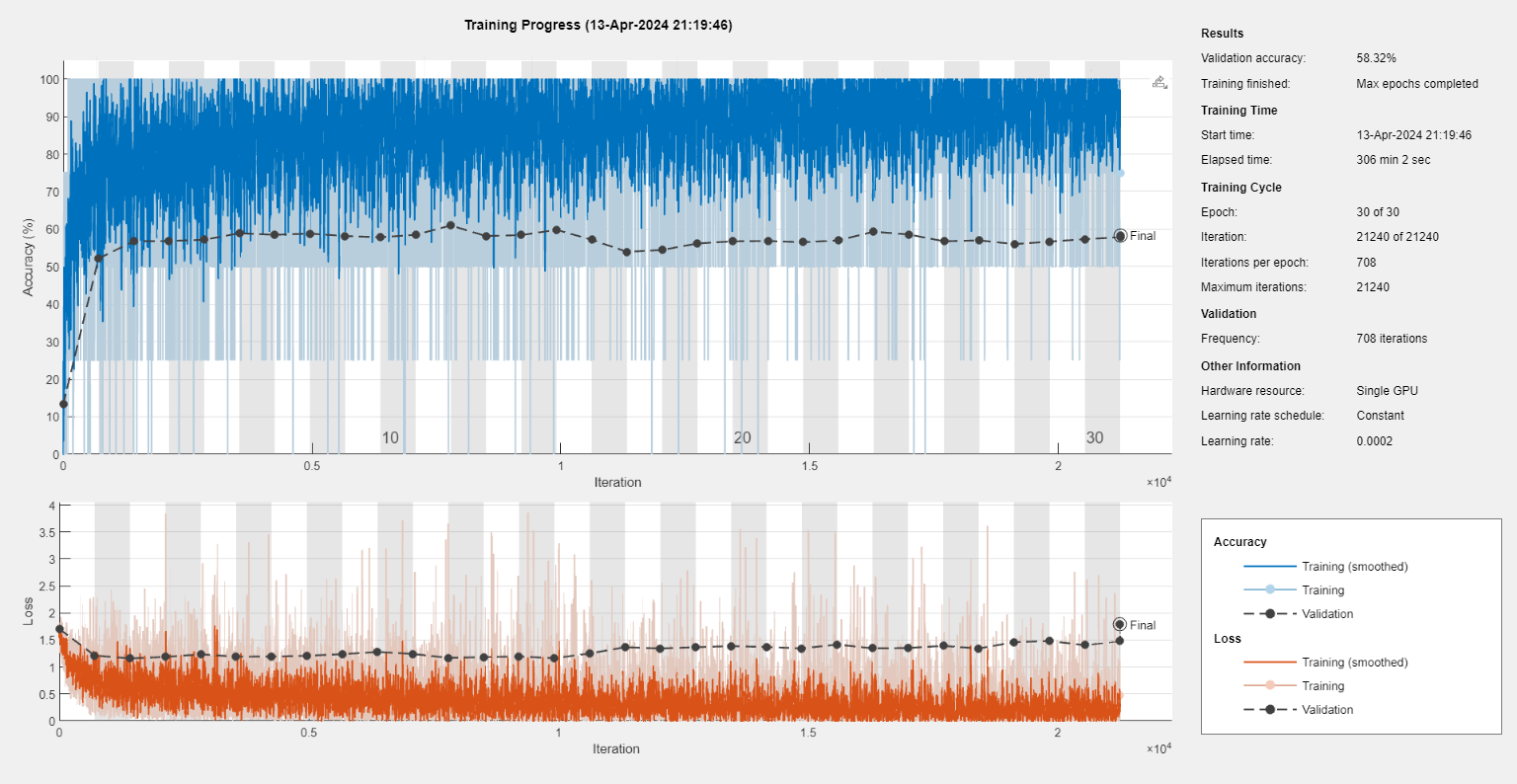 |
| --- | --- |
| 1. **MobileNet** | 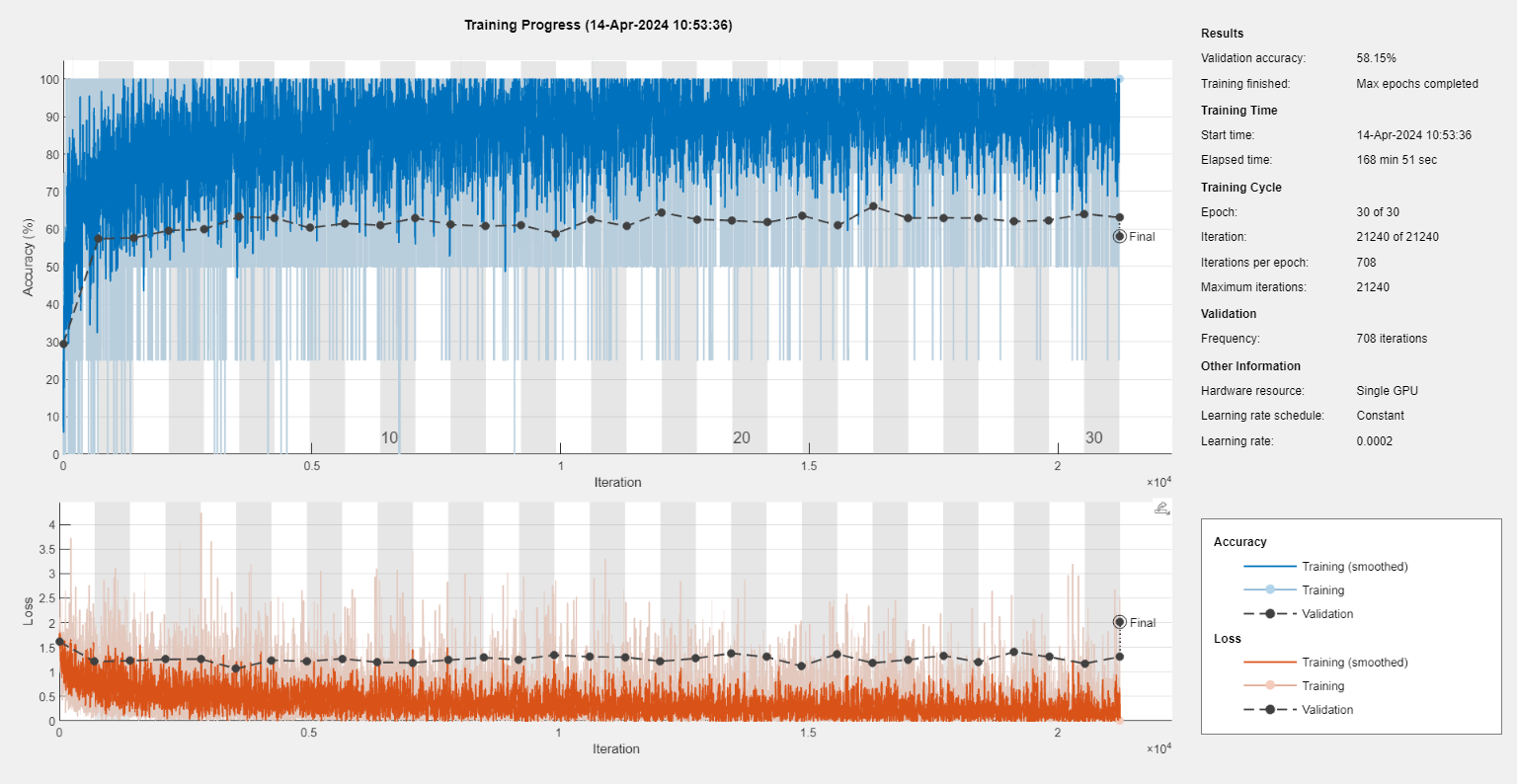 |
| 1. **ResNEt-18** | 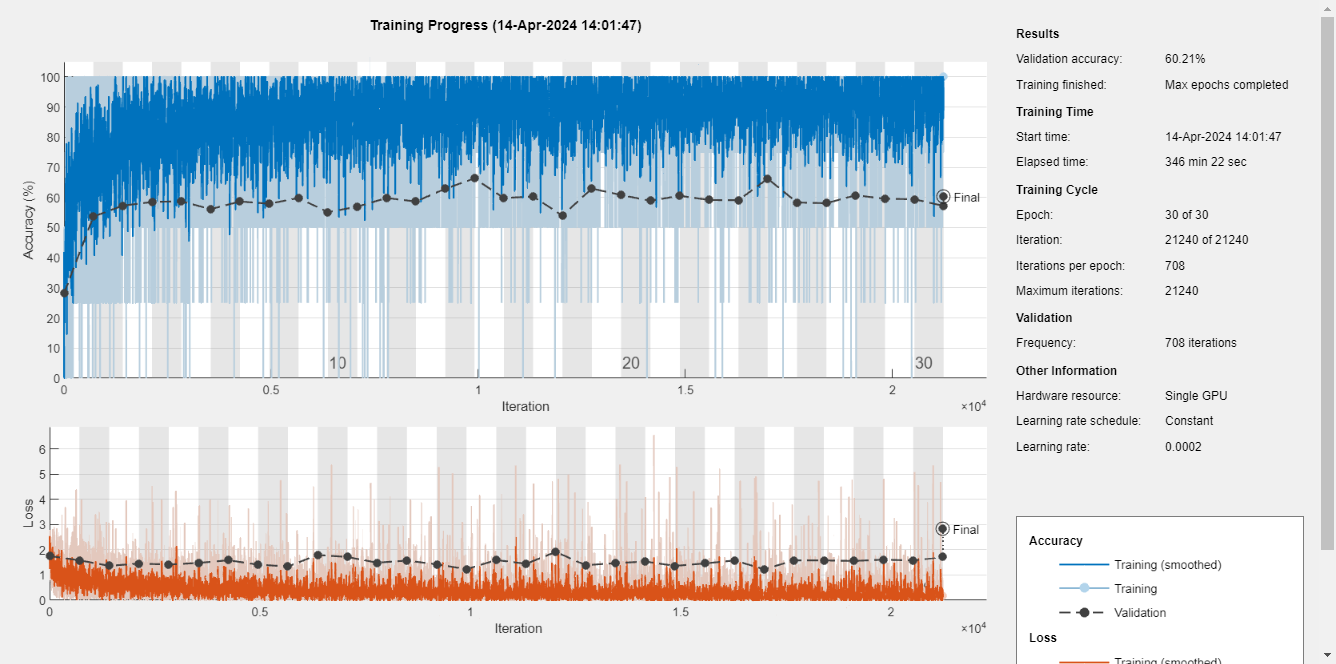 |

**Figure S.1** Training Validation Curves for (a) Efficient-B0, (b) MobileNet, (c) ResNet-18 trained on the SIPaKMeD dataset
