# Supplementary material for: Light-XAI: a CADx for explainable cervical cancer detection via attention-based lightweight convolutional neural networks and layer-wise feature fusion
Source: BioData Min. 2026 Apr 10;19:26. doi: 10.1186/s13040-026-00540-6 (PMC13085275; doi:10.1186/s13040-026-00540-6)
Supplement: Supplementary file 3 — Supplementary material 3 [file 13040_2026_540_MOESM3_ESM.docx]

| 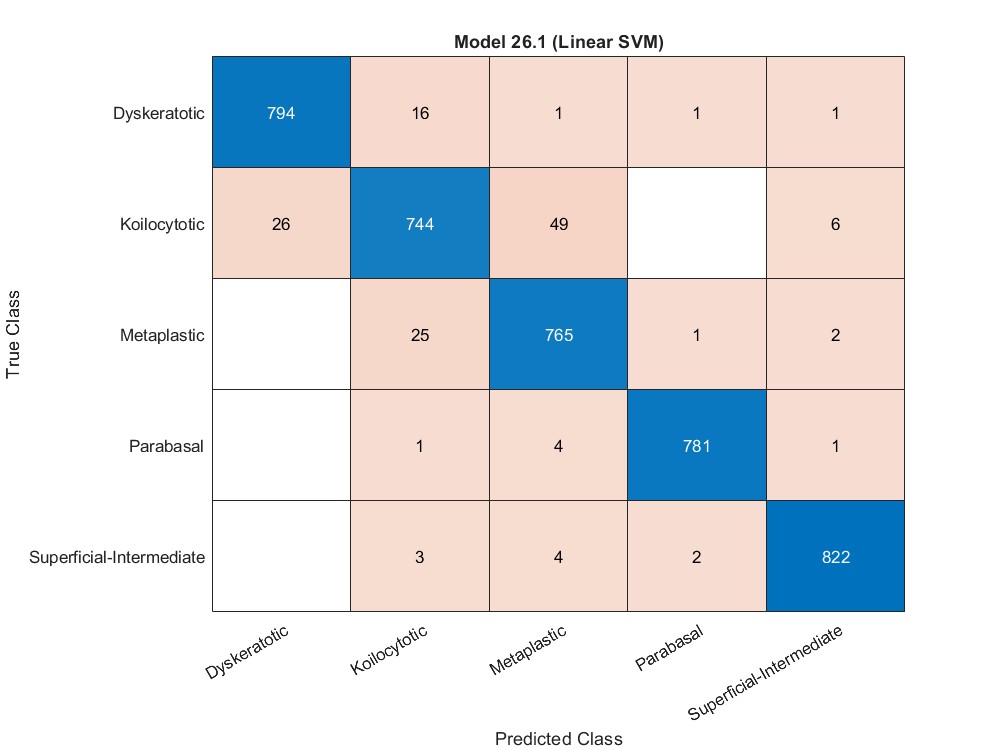 | 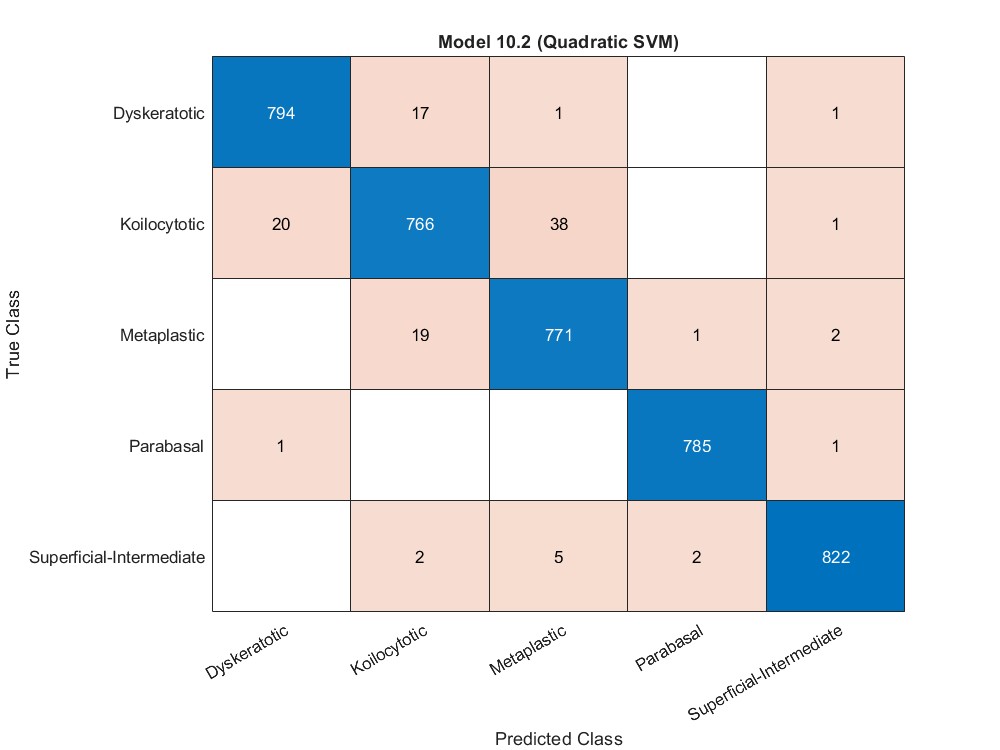 |
| --- | --- |
| **LSVM** | **QSVM** |
| 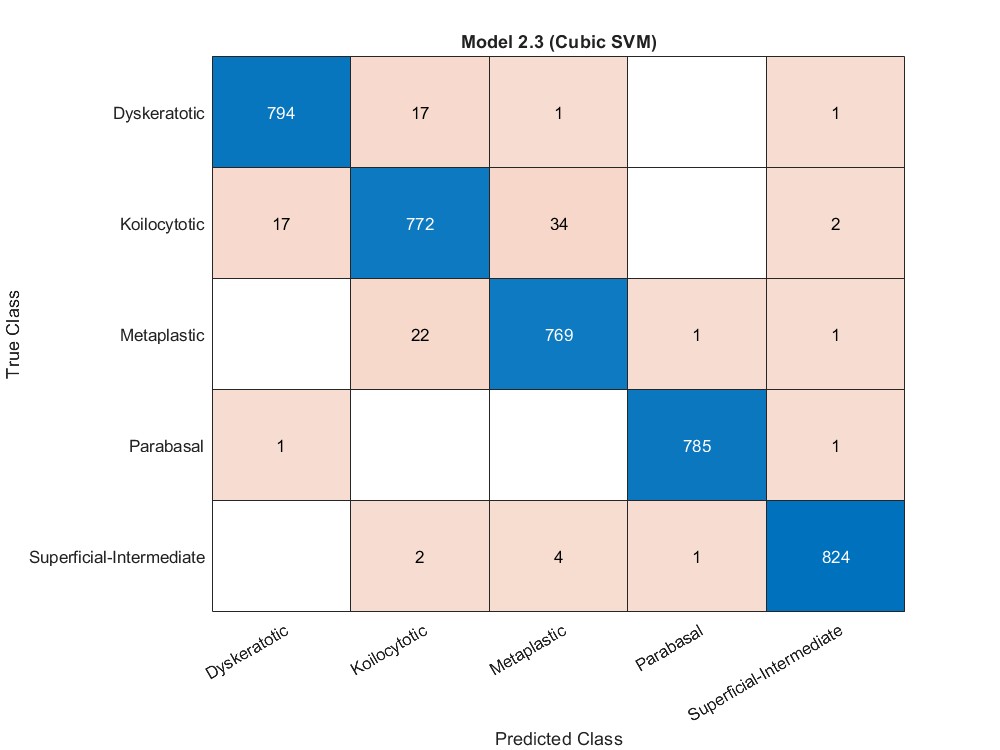 | 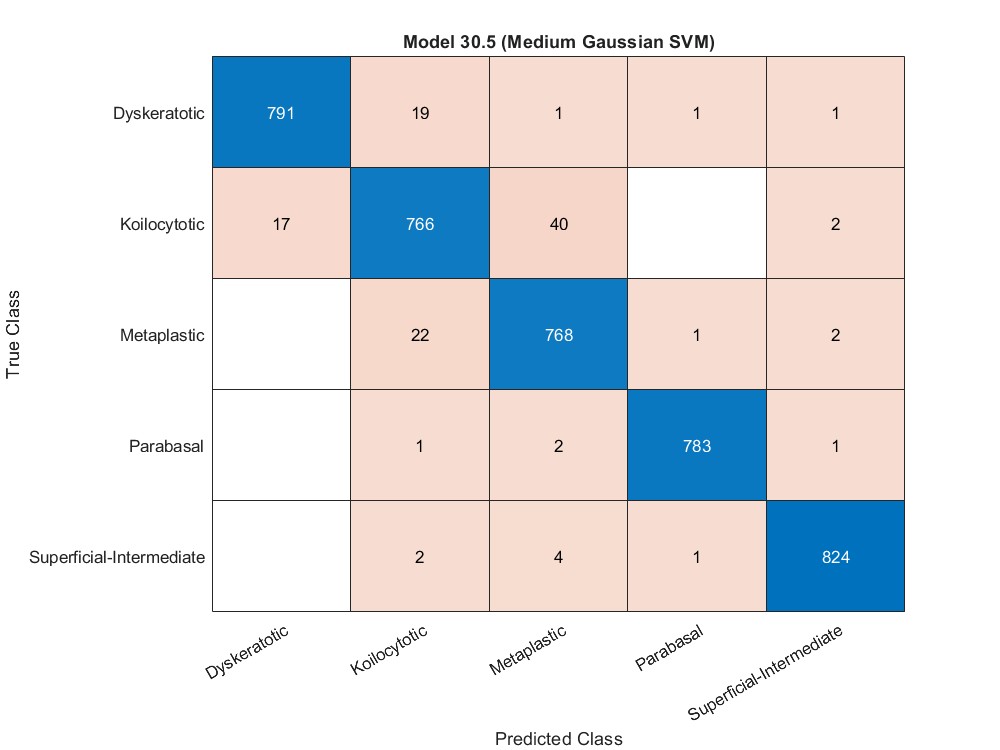 |
| **CSVM** | **GSVM** |
| 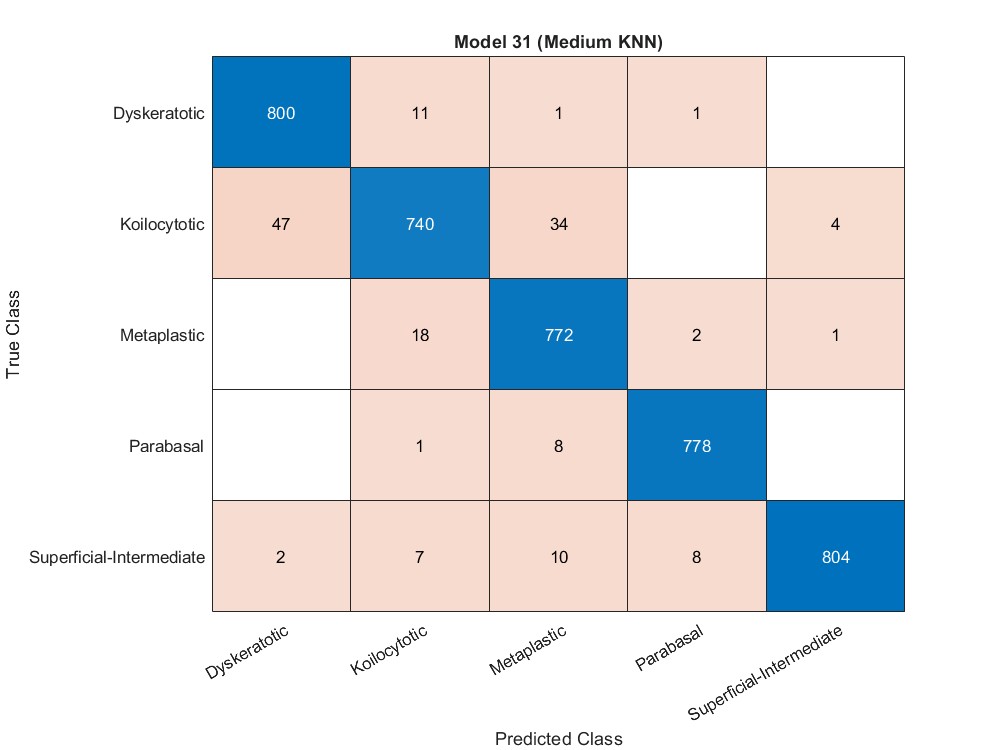 | 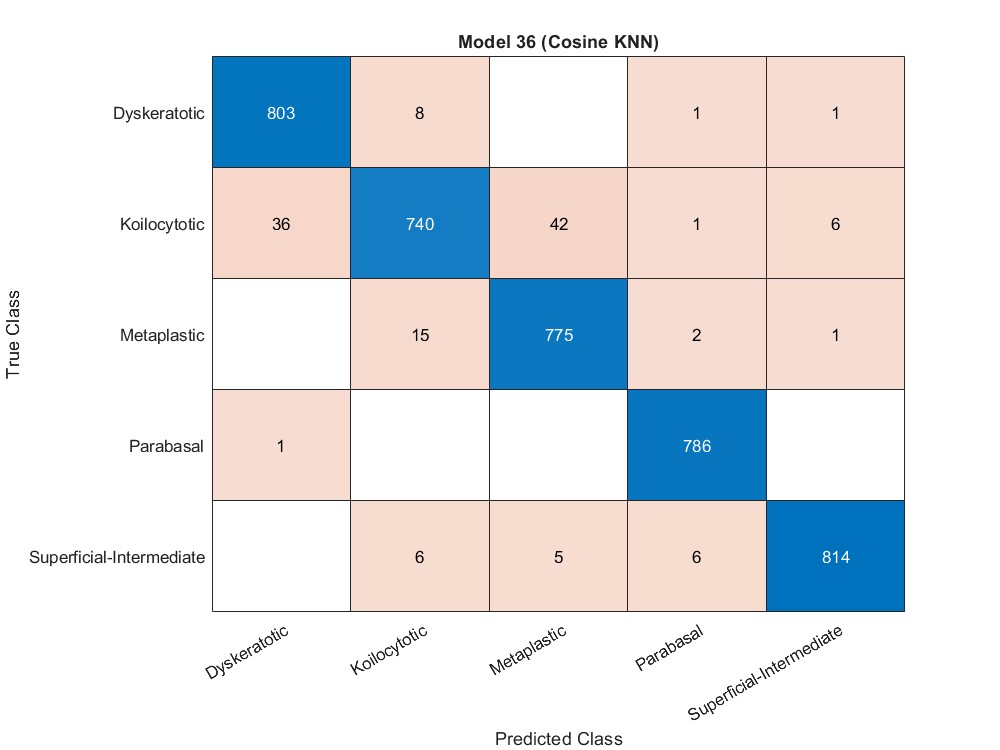 |
| **MKNN** | **CKNN** |
| 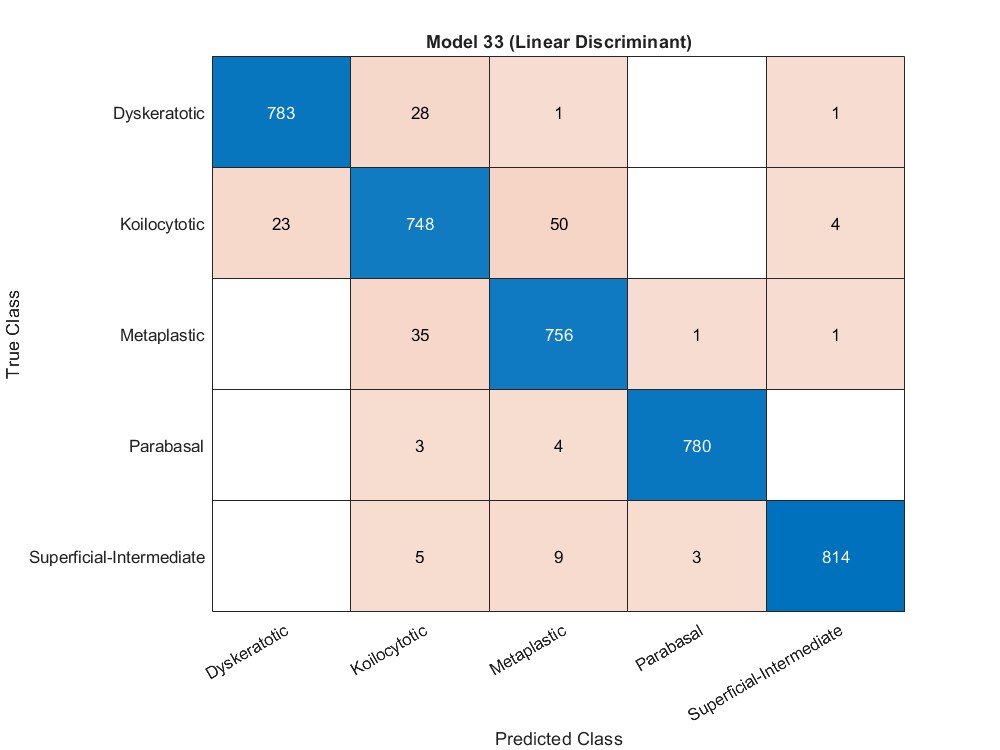 |  |
| **LDA** |  |

**Figure** S3 Confusion metrics (TP and TN vs. FP and FN) for the seven classification models learnt with the picked attributes of ANOVA feature selection from the SIPaKMeD dataset.
